# Effects of physical therapy modalities for motor function, functional recovery, and post-stroke complications in patients with severe stroke: a systematic review update

**DOI:** 10.1186/s13643-024-02676-0

**Published:** 2024-10-28

**Authors:** Katrin Roesner, Bettina Scheffler, Martina Kaehler, Bianca Schmidt-Maciejewski, Tabea Boettger, Susanne Saal

**Affiliations:** 1 Department of Physiotherapy, Pain and Exercise Research Luebeck (P.E.R.L.), Institute of Health Sciences, Universität zu Lübeck, Ratzeburger Allee 160, 23562 Lübeck, Germany; 2 International Graduate Academy (InGrA), Institute of Health and Nursing Sciences, Medical Faculty of Martin Luther University Halle-Wittenberg, University Medicine Halle, Magdeburger Straße 8, 06112 Halle (Saale), Germany; 3 Department of Therapy Science I, Brandenburg University of Technology Cottbus–Senftenberg, Universitätsplatz 1, 01968 Senftenberg, Germany; 4 Schön Klinik Hamburg Eilbek, Dehnhaide 120, 22081 Hamburg, Germany; 5 Executive Department for Nursing Competencies, Wilhelmsburger Krankenhaus Hamburg Großsand, Groß-Sand 3, 21107 Hamburg, Germany; 6 Department of Occupational Therapy, Institute of Health Sciences, Universität zu Lübeck, Ratzeburger Allee 160, 23562 Lübeck, Germany; 7 Ernst-Abbe-Hochschule Jena-University of Applied Science, Carl-Zeiß-Promenade 2, 07745 Jena, Germany; 8 Institute of Health and Nursing Science, Medical Faculty of Martin Luther University Halle-Wittenberg, University Medicine Halle, Magdeburger Straße 8, 06112 Halle (Saale), Germany

**Keywords:** Physical therapy, Effects, Dose, Severe stroke, Systematic review update

## Abstract

**Background:**

Physical therapy interventions play a crucial role in the daily care of patients recovering from severe stroke. However, the efficacy of these interventions and associated modalities, including duration, intensity, and frequency, have not been fully elucidated. In 2020, a systematic review reported the beneficial effects of physical therapy for patients with severe stroke but did not assess therapeutic modalities. We aim to update the current evidence on the effects of physical therapy interventions and their modalities in relation to the recovery phase in people with severe stroke in a hospital or inpatient rehabilitation facility.

**Methods:**

We searched CENTRAL, MEDLINE, Web of Science, and three other relevant databases between December 2018 and March 2021 and updated the search between April 2021 and March 2023. ClinicalTrials.gov and ICTRP for searching trial registries helped to identify ongoing RCTs since 2023. We included individual and cluster randomized controlled trials in the English and German languages that compared physical therapy interventions to similar or other interventions, usual care, or no intervention in a hospital or rehabilitation inpatient setting. We screened the studies from this recent review for eligibility criteria, especially according to the setting. Critical appraisal was performed according to the Cochrane risk-of-bias tool 2.0. The data were synthesized narratively.

**Results:**

The update identified 15 new studies, cumulating in a total of 30 studies (*n* = 2545 participants) meeting the eligibility criteria. These studies reported 54 outcomes and 20 physical therapy interventions. Two studies included participants during the hyperacute phase, 4 during the acute phase,18 during the early subacute phase, and 3 in the late subacute phase. Three studies started in the chronic phase. Summarised evidence has revealed an uncertain effect of physical therapy on patient outcomes (with moderate to low-quality evidence). Most studies showed a high risk of bias and did not reach the optimal sample size. Little was stated about the standard care and their therapy modalities.

**Discussion:**

There is conflicting evidence for the effectiveness of physical therapy interventions in patients with severe stroke. There is a need for additional high-quality studies that also systematically report therapeutic modalities from a multidimensional perspective in motor stroke recovery. Due to the high risk of bias and the generally small sample size of the included studies, the generalizability of the findings to large and heterogeneous volumes of outcome data is limited.

**Systematic review registration:**

PROSPERO CRD42021244285.

**Supplementary Information:**

The online version contains supplementary material available at 10.1186/s13643-024-02676-0.

## Introduction

Stroke is the second leading cause of death and the third leading cause of death and disability worldwide according to the Global Burden of Diseases Study Group [1]. An increase in stroke deaths and disability-adjusted life years (DALYs) will accompany future population growth and increased life expectancy in many countries [2, 3]. Simultaneously, stroke care has been optimized through specialized acute facilities (e.g., stroke units), and the advancement of recanalization therapies (i.e., thrombectomies and thrombolysis) has shown positive effects [4].

In 2017, the Stroke Recovery and Rehabilitation Roundtable Taskforce agreed on a standard definition of stroke phases [5]. This decision is based on current knowledge and understanding of the biological repair processes of the brain [6–8]. In animal models, a time dependency of recovery has already been observed [6]. It is hypothesized that the biological repair process begins early after the stroke and then slowly subsides. A similar course has also been observed in humans [6, 9].

However, this concept of stroke phases does not imply that functional recovery occurs linearly; rather, it is meant to ensure better comparability between research data sets. The proportion recovery rule (PRR) follows the hypothetical assumption that within the first 3–6 months, patients can improve on average by 70% (± 15%), and thus, the extent of recovery is highly predictable, regardless of the dose of therapy provided [10–12]. Some patients do not follow this rule, especially those who show severe deficits at baseline [13]. Recent approaches indicate that most people with stroke tend to experience a combination of constant recovery and proportional-to-spared function [14]. It was found that there are two different patterns of recovery for patients with stroke and different severities of initial motor impairment [15]. Severely and non-severely impaired patients exhibit individual recovery trajectories [15].

Reports on patients with severe stroke indicate that even after intensive and prolonged therapy, some patients may show little to no improvement [16]. According to a systematic review, people with severe stroke often progress more slowly and with less functional improvement during inpatient rehabilitation than people with mild impairment [17]. This could be due to the combination of significant sensory-motor and cognitive impairments induced by severe stroke [18]. Stroke severity is an important determinant of the length of hospital stay and is one of the most important indices for measuring the use of hospital care [19]. Several predictors of stroke recovery have been analyzed thus far, but therapy modalities, including duration, frequency, and intensity, have not been considered.

An important factor in understanding study outcomes can be the therapeutic modality of an intervention [20]. In the literature, there is a lack of definitions and heterogeneous use of the dose of intervention. In 2021, the group around Hayward [21] proposed a framework for dose articulation in stroke recovery: duration can measure the length of intervention in days, sessions per day, and session length with active or inactive episodes; an episode can be defined by the length of a task, its difficulty, and its intensity. For this reason, among others, studies have been conducted to examine the influence of duration and intensity of therapy and have shown positive treatment effects for a longer therapy duration. For example, depending on the author, the therapy time varied between upper extremity interventions post-stroke of 10 h/week, as measured by the Motor Activity Log (MAL) [22], and 30 h/week, as measured by the Fugl-Meyer-Assessment Upper Limb (FMA-UL) [23]. Little information is available about studies of people with severe stroke and associated therapy modalities, such as duration and episodes.

McGlinchey et al. (2020) [24] reported the beneficial effects of rehabilitation interventions for severely affected patients with stroke according to their stroke recovery phase and outcome measures according to the International Classification of Functioning, Disability and Health (ICF). His review did not address therapy modalities and included all kinds of settings. This review builds on and expands upon McGlinchey’s systematic review [24] by additionally evaluating the duration and episodes of interventions in people after a severe stroke in a hospital or inpatient rehabilitative setting.

## Methods

This systematic review was conducted following the recommendations of the Cochrane Handbook for Systematic Reviews of Interventions, version 6.3 [25] and was reported according to the Preferred Reporting Items for Systematic Reviews and Meta-Analysis Statement 2020 [26]. The protocol was registered prospectively at the Prospective Register of Systematic Reviews platform (CRD42021244285). There were no deviations from the protocol.

### Eligibility criteria

Studies were included when.

1. they were a randomized or non-randomized controlled trials. A randomized controlled trial can be an individually randomized trial (RCT), a cluster randomized controlled trial (cRCT) randomized crossover trial, or a multicenter randomized study. A non-randomized controlled trial can be mono- or multicentric, with a prospective study design. RCTs are considered the gold standard for demonstrating efficacy.
2. Study participants were aged ≥ 18 years and diagnosed with severe stroke (ischemic or hemorrhagic). For the purpose of this review, severe stroke was defined as Functional Independence Measure (FIM) score < 54 or early FIM < 40 or National Institute of Health Stroke Scale (NIHSS) ≤ 16 or modified Rankin Scale (mRS) ≤ 4 or Barthel Index (BI) ≤ 35 or Fugl-Meyer-Assessment (FMA) ≤ 55 or Functional Ambulatory Categories (FAC) ≤ 2 [27–32].
3. The placement of the intervention was within a hospital or inpatient rehabilitation facility.
4. Any kind of physical rehabilitation intervention was applied with the intention of managing physical problems following a medical or surgical condition.
5. A comparison intervention was reported, including any other type of physical rehabilitation or usual care.
6. At least one of the following outcomes was reported:
7. Functional recovery was defined as a partial or complete return to normal or proper physiologic activity of an organ or body part following disease or trauma and was assessed using a recognized outcome measure of functional ability or activities of daily living (ADLs) [33].
8. Motor function was defined as any activity of muscles due to stimulation by a motor neuron, movement, or activation and was assessed using a recognized measure of motor function [34].
9. Post-stroke complications, adverse events, and medical or neurological problems necessitate a physician’s order and require monitoring by medical staff [35, 36].
10. The study was published in English or German to ensure feasibility.

Studies were excluded if.

1. patients suffered a mild to moderate stroke.
2. Pharmacological, surgical, or complementary (e.g., acupuncture or non-invasive brain stimulation) interventions were used.
3. Interventions were delivered in an outpatient setting (e.g., home or ambulatory).

### Search strategy and selection criteria

Since the review builds up on the systematic review of McGlinchey et al. [24] a search was conducted for studies published between January 1987 and November 2018), and the search strategy was based on their report. For this reason, we have not consulted any other experts. Electronic searches were also conducted in the Medical Literature Analysis and Retrieval System Online (MEDLINE), Excerpta Medica Database (EMBASE), Cumulative Index to Nursing and Allied Health Literature (CINHAL), Physiotherapy Evidence Database (PEDro), Web of Science, Database of Research in Stroke (DORIS) and the Cochrane Central Register of Controlled Trials (CENTRAL) from December 2018 to March 2021 and an update from April 2021 to March 2023. The search strategy included identifying the terms related to severe stroke, physical therapy modalities, stroke rehabilitation, motor recovery, and motor function. Information specialists were consulted. An example of the search strategy can be found in Supplementary material S1. The search was restricted to human studies. The International Trials Registry Platform (ICTRP) and ClinicalTrials.gov helped to identify ongoing trials from January 2019 to March 2021 and were updated from April 2022 to March 2023. To identify further studies, reference lists were searched, and a forward citation search was carried out on the Web of Science, Google Scholar, and Scopus.

## Data collection and analysis

### Study selection

All eligible studies were uploaded to a reference management program. After duplicate removal, the remaining studies were uploaded to Rayyan [37]. Using the prespecified criteria for eligibility, two authors (KR, BSM) independently screened the studies for inclusion based on their titles and abstracts. Two independent reviewers (KR, MK) screened the full texts of the studies identified as eligible for inclusion during title and abstract screening. The reasons for the excluded studies are listed in a table (Supplementary material S2). Reports from the same study population were linked to ensure that data from the same population were only included once in the review and analysis. Discrepancies were discussed and resolved by consensus with a third reviewer (SuS).

### Data extraction and management

Two review authors (KR, TB) independently extracted the data from all the included studies. A prespecified data extraction form was developed based on the Cochrane Handbook for Systematic Review of Interventions (version 6.3), the CONSORT statement for reporting randomized trials and extensions, and the Template for Intervention Description and Replication (TiDieR) [25, 38, 39]. The data extraction form was pilot-tested during two online face-to-face training sessions. The review by McGlinchey et al. [24] did not report the therapeutic modalities; therefore, we screened all the studies according to the setting, stroke severity cut-off points, and interventions. The following information was extracted: aim and focus of the studies, study design, details about the intervention according to the TiDieR Checklist [39], number and characteristics of participants, time post-stroke (converted in days), outcomes, and outcome measures. The individuals in each study were assigned to the following post-stroke recovery phases: hyperacute (≤ 24 h post-stroke), acute (> 24 h but ≤ 7 days post-stroke), early subacute (> 7 days but ≤ 3 months (≤ 90 days) post-stroke) and late subacute (> 3 months but ≤ 6 months (≤ 180 days) post-stroke) [4]. Funding sources for the studies were collected in tabular form (Supplementary material S3).

### Risk of bias

Two authors (BS, KR) independently rated the risk of bias using the Cochrane Risk of Bias 2 (RoB2) tool for individually randomized, parallel-group trials [40] and recommended according to the “SHORT VERSION (CRIBSHEET)” [41]. An overall judgment of a high risk of bias was given when the study was judged to be at high risk of bias in one domain or if there was some concern for multiple, in this review, two domains. Any differences in opinion were discussed between the two authors and were recorded in writing. There was no need to consult a third reviewer. McGlinchey [24] stated in his review that a high quality meant a low risk of bias, a moderate quality a serious risk and a low to very low quality of evidence was based on a high risk of bias.

### Reporting on intervention and dose

Extraction according to the TiDieR checklist [39] of the goal of the interventions included who and what was provided, whether it was tailored or modified, how well it was planned, and whether there was economic information available. Item eight of the TiDieR instrument evaluates the number of times the intervention was delivered and the duration, intensity, or dose during which the intervention was delivered. In addition, further dose dimensions such as session density and episodes were extracted [42].

### Data analysis and synthesis

Demographics and study results are reported as medians (IQRs), minimum to maximum ranges, or numbers of studies (percentages) as appropriate. Due to the heterogeneity of outcome measures, recovery phases, therapy modality outcomes, and the high proportion of studies where concerns regarding bias were present, pooled analyses were not performed. The extracted data are summarised in tables as narrative descriptions of the intervention and therapy modalities by recovery phase. If various outcomes were reported, the means and standard deviations, including participant characteristics and test results, were combined into one group using the free accessible Statistics Toolkit (STATTOOLS, Palisade, Ithaca, NY) [43]. This procedure of combining means (SDs) complies with the recommendations of the *Cochrane* Handbook for Systematic Reviews of Interventions (version 6.3) [25]. To judge the quality of evidence narratively, the Grading of Recommendations, Assessment, Development and Evaluation (GRADE) approach was used [44, 45]. 

## Results

### Study selection

**Fig. 1:**
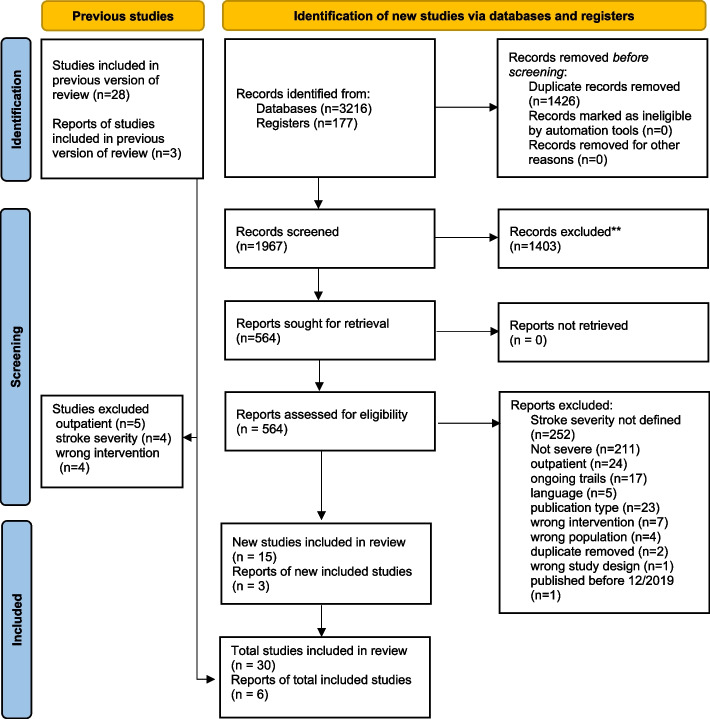
Preferred Reporting Items for Systematic Reviews and Meta-Analysis 2020 study selection flow diagram

Out of 3216 identified records, 564 full-title articles were screened. The search was conducted between the 11th and 23rd of April 2021 and the 1st and 10th of April 2023. This review included 30 studies, 15 of which were also included in McGlinchey’s analysis [46–69]. However, 13 studies included by McGlinchey were excluded in this current review due to an incorrect setting, intervention, or different cut-offs for the severity of stroke. Two reports [47, 48] were subanalyses from the AVERT study [49], and two [50, 51] were follow-up studies to Kwakkel et al. (1999 [52]). No further results were found through searching reference lists or forward citations in the Web of Science, Google Scholar, or Scopus databases. Figure 1 shows the results of the screening procedure.

### Study characteristics

Study characteristics, including the interventions used, are provided in Table 1. All the studies were published between 1999 [52, 53] and 2023 [54] and were mostly conducted in Italy [55–58], Korea [46, 59–64], and China [54, 65–69]. A total of 2545 participants were included across all the RCTs (range: *n* = 20 [70] to *n* = 294 [49]). The overall study duration ranged between one [71] and 20 weeks [52].

**Table 1:** Result

Reference	Study design, country	sample, age in years, percent female (♀%)	Stroke severity measure	Intervention and severity characteristics	Delivered by	Outcome	Results
**Global early mobilisation within 24 h**							
AVERT Trail Collaboration Group, 2015 [49]; Bernhardt et al., 2021 [5], Cain et al., 2022 [48, 47]	Parallel-group multicentre RCT Australia, New Zealand, Malaysia, Singapore, and the UK	n= 294 No information for severe stroke patients	NIHSS	**Intervention** very early mobilisation NIHSS >16 (*n*=147) **Control** usual care NIHSS >16 (*n*=147)	PTs and nurses	Favourable outcome (mRS 0-2) and mortality at 3 months	Subgroup analysis for severe stroke patients concerning mRS favours usual care over early mobilisation with an Odds Ratio of 0.35 (0.11–1.18). 88 (8%) patients died in the very early mobilisation group and 72 (7%) patients in the usual care group.
Di Lauro et al., 2003 [57]	Individual RCT Italy	*n*= 60 **Intervention** 69,30 (±8,0) ♀ 62% **Control** 67,6 (±9,3) ♀ 55%	BI*	**Intervention** Intensive rehab group Mean BI 1.4 (±1.4) (*n*=29) **Control** Ordinary rehab group (*n*=31) Mean BI 1.5 (±1.5)	Therapists and nursing staff	BI, mNIHSS	No differences between groups in BI at 180 days (IG: 8.0±2.8; CG: 7.7±3.0; p>0.7) or NIHSS at 14 days (IG: 8.1±3.0; CG: 8.4±2.6; p>0.6) and at 180 days (IG: 6.2±2.8; CG: 6.5±2.7; p>0.7).
**Electrical Stimulation**							
Guo and Kang, 2018 [65]	Individual RCT China	n= 82 **Intervention** 64.3 (±11.8) ♀ 46% **Control** 62.5 (±12.2) ♀ 39%	BI	**Intervention** NMES (*n*=38) Mean BI 10,5 (±2,2) **Control** sham NMES (*n*=36) Mean BI 10,8 (±2,6)	not described	BI, ICIQ-SF, OBASS, Urodynamic outcome	The results of the intervention group are more promising than the control group results in all outcomes: Urodynamic value (*p*<0,01), OABSS: IG: 8.1±3.4; CG: 12.3±3.0; p<0.01, c: IG 7.8±3.3; CG: 10.5±3.1; *p*<0.01, BI: IG:15.7±3.1; CG: 11.1±3.4; *p*<0.01.
Rosewilliam et al., 2012 [72]	Individual RCT USA	n=90 entire sample 74,6 (±11,0) ♀ 56%	BI^	**Intervention** NMES group (*n*=31) Mean BI 4.4 (±3.9) Control Usual care group (*n*=36) Mean BI 2.5 (±2.9)	NMES- staff group not reported, patients and carers, Usual care- PTs	ARAT, BI, wrist AROM, wrist strength, grip strength	No differences in ARAT, BI or wrist AROM between groups. Improvements in wrist extensor and grip strength in the NMES group post-intervention but not maintained at follow-up.
Zheng et al., 2018 [66]	Individual RCT	n= 60 **Intervention** 59 (±11) ♀ 50% **Control 1** 60 (±9) ♀ 40% **Control 2** 59 (±9) ♀ 40%	mBI	**Intervention** four-channel FES group (*n*=18) Mean mBI 22 (±9) **Control 1** dual-channel FES group (*n*=15) Mean mBI 23 (±13) **Control 2** placebo group (*n*=15) Mean mBI 24 (±13)	PTs	mBI, FMA, PASS, BBS, BBA, mBI, fMRI	In favour of four-channel FES group showed fractional anisotropy and increased fibre bundles. No significance between-group differences.
**Mirror therapy**							
Cui et al., 2022 [69]	Individual RCT China	n=32 **Intervention** 61.5±9.93 ♀ 44% **Control** 58.5±11.15 ♀ 50%	mBI	**Intervention** mirror therapy on lower leg (*n*=16), range mBI 21.50 (20.00, 25.75) **Control** routine rehabilitation (*n*=16), range mBI 22.50 (10.00, 27.75)	Therapists	FMA-LE, BBS, mBI, mRMI, rs-fMRI	A better effect for the mirror therapy group concerning FMA-LE (Z= -4,526,*p*<0,01), BBS (F = 36.985, *p *< 0.01), mMRI (F = 27.171, *p *< 0.01), mBI (F = 9.830, *p *= 0.004).
Lee et al., 2020 [60]	Individual RCT Korea	*n*=21 **Intervention** 50.91 (±8.73) ♀ 38% Control 61.5 (±9.93) ♀ 28%	K-mBI	**Intervention** multi-joint-based mirror therapy (*n*=11), Mean K-mBI 23,73(± 7,70) **Control** single-joint based mirror therapy (*n*=10), Mean K-mBI 18,80 (± 7,22)	OTs	K-mBI, FMA, MAL	A favourable outcome for improving upper limb function (FMA-UE) and ADL in IG compared tcontrolrl. FMA-UE: IG:26.36±11.75; CG: 16.00 ±8.7; *p*= 0.034 MAL-QoM: IG: 20.91±12.8; CG: 12 ±6.2; *p*=0.034 MAL-AOU: IG: 17.64±72.8; CG: 11.4±6; *p*=0.048 mBI: IG: 26.55±5.71; CG: 19,4±7.18; *p*= 0.031
Sim and Kwon, 2022 [62]	Individual RCT Korea	*n*=30 **Intervention** 69.29 (±8.02) ♀ 33% **Control** 69.14 (±6.92) ♀ 36%	K-mBI	**Intervention** bimanual mirror therapy (n=14), Mean K-mBI 35.64 (±16.08) **Control** unimanual mirror therapy (n=14), Mean K-mBI 30.92 (±11.57)	OTs	MVPT, K-MMSE, BIT, K-CBS, K-mBI, SCT, LBT	In favour of the intervention group was found for SCT (p<0,05), PST (p<0,05), for LBT (p<0,05) and for K-CBS (p<0,05). No differences were found for K-mBI.
**Neurodevelopmental techniques**							
Bai et al., 2014 [67]	Individual RCT China	n=165 **Intervention** 67,63 (±9,52) ♀ 38% **Control** 66.04 (±10,13) ♀ 38%	mBI	**Intervention** 1.-3. month of staged rehabilitation group (n=83) Mean BI 28 (range 24-31) **Control** Routine care group (n=82) Mean BI 23 (range 19-27)	PTs and OTs	mBI, mAS	IG demonstrated higher mBI scores than the routine care group at 1, 3- and 6 months post-stroke. IG: (M1 vs. M0, M3 vs. M1, M6 vs. M3, p< 0.01); CG: (M1 vs. M0, p <0.01; M3 vs. M1, p = 0.026) 42.9% of patients in the CG demonstrated spasticity in at least one body part compared to 36.4% of patients in the staged rehab group.
Rahayu et al., 2020 [71]	Individual RCT Indonesia	n=64 **Intervention** 58.84 (±8.68) ♀ 38% **Control** 59.93 (±10.65) ♀ 50%	BI	**Intervention** Neurorestoration intervention (n=32) Mean BI 25.81 (±15.77) **Control** standard procedure (n=32) Mean BI 19,00 (± 10.29)	Research Assistant	BI, BNDF-Biomarker, BBS	In favour of intervention group for functional performance (BI: IG 67. 47 (58.99-75.94; CG: 46.41 (37.77-55.04); *p*=0.008) and balance (BBS: IG: 28.38 (21.74-35.01; CG:17.16 (12.62-21.69; p=0.016) in between group difference. No differences in neuroplasticity regeneration (p=0.07).
Tang et al., 2014 [68]	Individual RCT China	n=48 **Intervention** 68,2 (±4,1) ♀ 29% **Control** 66,9 (±4,1) ♀ 33%	STREAM, BBS	**Intervention** Early contemporary Bobath group (n=24) Mean STREAM 1.4 (± 1.0), Mean BBS 0 (± 0) **Control** Contemporary group (n=24) Mean STREAM 1.3 (± 0.9), Mean BBS 0 (± 0)	PTs	STREAM, BBS	Improvements in STREAM (F (1, 46) = 11.7, η2 = 0.203, p < .01) and BBS (F (1, 46) = 35.4, ŋ^2^ = 0.435, p < .001) in the contemporary Bobath approach with early mobilisation group.
**Interventions for verticalization**							
Bagley et al., 2005 [73]	Individual RCT UK	n= 140 **Intervention** 75,8 (±11,5) ♀ 29% **Control** 75,1 (±9,4) ♀ 31%	BI	**Intervention** Oswestry group (*n*=71) Median BI 1 (IQR 0-3) **Control** Control group (*n*=69) Median BI 2 (IQR 1-3)	PTs and nurses	RMI, BI, HADS NEADL, RMA, MAS (balance, sit-to-stand sections), TCT, CSI, GHQ-28	No differences between groups for all outcome measures. No difference in the number of treatment sessions or stuff required for treatment.
Calabró et al., 2015 [56]	Individual RCT Italy	*n*=32 **Intervention** 71 (±3) ♀ 60% **Control** 70 (±5) ♀ 50%	PASS, FMA-LL	**Intervention** Robotic verticalization group (*n*=10) Mean PASS 3 (±1), Mean LL FMA 13 (±3) **Control** Physiotherapy group (*n*=10) Mean PASS 3 (±3), Mean LL FMA 12 (±6)	PTs	PASS, FMA-LL, RCPM, MRC, vertical posture tolerance	Both interventions were well, tolerated. The robotic group demonstrated greater improvements compared to the physiotherapy group in: MRC (IG: 2±1; CG: 1±1; p=0,03); FMA (IG: 92±10; CG: 58±7; *p*=0.008) and PASS (IG: 166±30; CG: 66±2; *p*=0.008). Between-group differences for cognition were measured with RCPM of *p*=0.03.
Logan et al., 2022 [74]	Individual RCT UK	*n*= 45 **Intervention** 81.7 (±11.7) ♀ 27% **Control** 78.9 (±10.5) ♀ 31%	mRS	**Intervention** Functional standing frame programme (*n*=22) mRS 4= 17; mRS 5= 5 **Control** usual physiotherapy (*n*=23) mRS 4= 19; mRS 5= 4	PTs	Edmans ADL, BI, Goniometer, muscle strength, MAS, TCT, VAS for fatigue, PHQ-9, SADQ-10, EQ-5D 5L, Stroke and Aphasia QoL Scale-39	It was a feasibility trial. It is not feasible in its current design. The intervention group showed some promising results on the BI for example at the 55^th^ week with a Mean difference (95% CI) of 0,86 [-4.76, 6.49], and showed over time a ≥ 1.85 point minimal clinically important difference.
**Gait training interventions without electrical support**							
Brunelli et al., 2019 [55]	Individual RCT Italy	*n*= 37 **Intervention** 69.64 (±10.88) **Control** 72.05 (±10.08) Overall ♀ 52%	FAC	**Intervention** BWS overground gait training (*n*=16), Mean BI 14.35 (±14.62) **Control** Gait training without BWS (*n*=21), Mean BI 14.42 (±15.72)	PTs	FAC, RMI, BI 6MWT	Patients in both groups improved continuously. No difference between groups (*p*>0.05) in independence in walking (FAC), or any secondary outcome (*p*>0.05).
Kim et al., 2020 [46]	Individual RCT Korea	*n*= 22 **Intervention** 65.2 (11.9) ♀ 45% **Control** 61.4 (10.9) ♀ 18%	FAC	**Intervention** underwater gait training (*n*=10) FAC<3 **Control** overground gait training (*n*=11) FAC <3	PTs	FAC, PASS, Balancia 2.0 program, GAITRite system	No favourable outcome for PASS, Postural control between the groups (*p*>0.05). The step length difference varied between groups, increased in IG and decreased in CG (IG: 4.55±6.68; CG: -1.25 ±3.56; *p*<0.05).
**Robotic-assisted gait training**							
Chang et al., 2012 [61]	Individual RCT Korea	*n*= 48 **Intervention** 55,5 (±12) ♀ 29% **Control** 59,7 (±12.1) ♀ 29%	FAC, FMA-LL	**Intervention** Robot-assisted group (n=20) Mean FAC 0.5 (±0.5) **Control** Conventional group (*n*=17) Mean FAC 0.4 (±0.5)	PTs	FAC, LL MI, FMA-LL, Peak chang	Between-group differences for intervention group in FMA-LL (IG: 22.7 ±5.7; CG 19.6±5.6; *p*=0.037) and peak VO2 (l/min, IG:1.23±0.44; CG: 1.11±0.46; *p*=0.025). No improvements in LL MI (IG: 56.2±11.0; CG: 53.5±12; *p*= 0.200) and FAC (IG 0.5±0.5; CG: 1.4±0.8; *p*=0.232).
Francesschini et al., 2009 [58]	Individual multicentre RCT Italy	*n*= 97 **Intervention** 65,5 (±12,2) ♀ 46% **Control** 70,9 (±11,8) ♀ 51%	BI*	**Intervention** Treadmill training group (*n*=52) Median BI 6 (IQR 3-9), Median FAC 0 (IQR 0-0) **Control** Conventional group (*n*=45) Median BI 5 (IQR 3-7), Median FAC 0 (IQR 0-0)	PTs	MI, TCT, mRS, BI, FAC, AS, LL proprioception, 6MWT, 10MWT, BS, WHS	No differences between groups.
Louie et al., 2021 [75]	Individual multicentre RCT Canada	n= 36 **Intervention** 59.6 (15.8) ♀ 16% **Control** 55.3 (10.6) ♀ 41%	FAC	**Intervention** Exoskelet group (*n*=19), Median FAC 0 (0-1) **Control** usual physiotherapy group (*n*=17), Median FAC 0 (0-1)	PTs	FAC, 5MWT, 6MWT, FMA-LL, BBS, MoCA, SF-36	No significant between-group differences for FAC. But significant effects for the intervention group on FMA-LL(as-treated adjusted group difference: 3.9, 95% CI 1.3–6.6, F(1,33) = 9.33, *p *= 0.004; per-protocol adjusted group differenci: 3.7, 95% CI 0.9–6.5, F(1,28) = 7.29, *p *= 0.01) and MoCA (as-treated adjusted group difference: 2.1, 95% CI 0.6–3.7, F(1,29) = 7.96, *p *= 0.009; per-protocol adjusted group difference: 2.0, 95% CI 0.4–3.6, F(1,25) = 6.62, *p *= 0.02). No further significant between-group differences in secondary outcomes.
Ochi et al., 2015 [76]	Individual RCT Japan	*n*= 26 **Intervention** 61,8 (±7,5) ♀ 15% **Control** 65,5 (±12,1) ♀ 31%	FIM mobility, FAC	**Intervention** Robot-assisted treadmill gait training group (*n*=13) Median FAC 0 (IQR 0-1), Median FIM mobility 7 (IQR 6-10) **Control** Conventional group (*n*=13) Median FAC 1 (IQR 0-1), Median FIM mobility 7 (IQR 7-9)	Robot-assisted gait training not reported, conventional gait training PTS	FAC, FMA, LL muscle torque, 10MWT, FIM(mobility scores)	Robot-assisted gait training group demonstrated greater improvements in FAC (IG: 3 (3-4); CG: 3 (3-3); p=0.02) and peak LL muscle torque (IG: 0.37 (0.2-0.52); CG: 0.18 (0.09-0.23; p=0.05compared to the conventional group.
Rodrigues et al., 2017 [70]	Individual RCT USA	*n*=20 **Intervention** 59,3 (±13,8) ♀ 50% **Control** 50,6 (±14,4) ♀ 40%	FMA-LL, FAC	**Intervention** Robot-assisted BWS treadmill gait training with increasing speed fast group (*n*=10) Median FAC 1.5 (1–2), Mean FMA-LL 19.5 (±4.6) **Control** Slower speed group (*n*=10) Median FAC 1 (1–2), Mean FMA-LL 17.5 (±2.8)	Not reported	FAC, TUG, 6MWT, 10MWT, BBS, FMA-LL	Improvements in FAC, FMA-LL, TUG and 6MWT in the slow group compared to the fast group. The fast group led to better outcomes on BBS. No between group difference is recorded.
Thimabut et al., 2022 [77]	Individual RCT Japan	n=26 **Intervention** 52.8 (±12.6) ♀ 23% **Control** 62.8 (±8.5) ♀ 40%	BI	**Intervention** Robotic-assisted gait device (*n*=13), Mean BI 10 (±2.61) **Control** Control group (*n*=13), Mean BI 11.23 (±2.31)	PTs	FIM walking, 6MWT, mBI, gait parameters (Xsens)	In favour of the intervention group for FIM walk score in between-group comparison at the end of the 15^th^ session (5.00±1.29 vs 3.46±1.76, P=.012), but no differences were detected at the second half. No differences for the 6MWT. But between-group differences for ADLs (BI: (7.31±1.89 vs 4.62±0.96, *P*<.001).
**Diverse interventions**							
An et al., 2021 [64]	Individual RCT Korea	n=30 **Intervention** 60,5 (±6,0) ♀ 27% **Control** 64.7 (±6,9) ♀ 30%	k-mBI	**Intervention** whole-body tilting postural training (*n*=15), Mean K-mBI 23,4 (±8,0) **Control** General postural training group (*n*=15), Mean K-mBI 17,8 (±11,1)	PT	BLS, PASS,K-mBI, BBS, FMA-LL	All outcomes showed a significant between-group difference in favour for the intervention group.
Chen et al., 2011 [78]	Individual RCT Taiwan	*n*=35 **Intervention** 58,0 (±11,5) ♀ 23% **Control** 62,3 (±11,35) ♀ 43%	FAC, FMA-LL	**Intervention** Thermal stimulation group (n=17) Median FAC 0 (IQR 0-1), Median LL FMA 7 (4-11.5) **Control** Standard rehab group (*n*=16) Median FAC 0 (IQR 0-1), Median LL FMA 6 (4.3-12.0)	Thermal-stimulation- PTs	FMA-LL, MRC-LL, mMAS, PASS (trunk control items), BBS, FAC	Thermal stimulation group demonstrated greater recovery gains compared to standard care in all outcomes except PASS. FMA-LL: IG: 14(10.5-15.5); CG:6.0(3-9.8); *p* <0.001 MRC-LL: IG: 6(4-7);CG: 3(1.3-4); *p*<0.001 mMAS: IG 16(12.5-18.5); CG: 10.5(5.3-14); *p*=0.01 BBS: IG: 28(20.5-33.5); CG: 15.5(9.3-23.5); *p*=0.007 FAC: IG: 2(2—2); CG: 1(1-1); p<0.001
Choi et al., 2021 [59]	Individual RCT Korea	*n*=24 **Intervention** 63.00 (±10.02) ♀ 58% **Control** 61.58 (±9.99) ♀ 50%	mBI	**Intervention** Digital Practice group (*n*=12) Mean mBI 37.42 (±8.73) **Control** Control group (*n*=12) Mean mBI 38.08 (±9.80)	Therapists	LBT, CBS, MVPT-V, head-tracking sensor data, mBI	Digital practice with VR rehabilitation system led to greater recovery of self-awareness of behavioural neglect, cognitive and visual perception. Between-group differences in LBT-score (IG: 11.75±5.83; CG: 9.67±6.61; *p*=0.02). No differences in mBI (*p*=0.52) and CBS (*p*=0.143).
Katz-Leurer et al., 20003 [79]	Individual RCT Israel	*n*=92 **Intervention** 65,5 (±12,2) ♀ 48% **Control** 70,9 (±11,8) ♀ 46%	SSS	Leg cycle ergometer and regular rehabilitation groups- actual number of patients with severe stroke (SSS <30) not reported	Leg cycle ergometer- PTs	FAI	No differences in FAI between groups.
Kim et al., 2022 [63]	Individual RCT Korea	n=41 **Intervention** 64.76 (±12.80) ♀ 38% **Control** 63.60 (±14.46) ♀ 50%	K-mBI	**Intervention** Elastic dynamic shoulder sling group (*n*=21) Mean K-mBI 35.00 (±17.85) **Control** Bobath sling group (*n*=20) Mean K-mBI 30.90 (±20.50)	Not further defined	X-Ray, FMA, K-mBI, VAS pain, MAD, MMT	There was a significant between-group difference (-0,80±3,11 vs. 2,28±3,66, p=0,006) in favour of the intervention group. No further differences had been detected.
Kwakkel et al., 1999 [52], 2002a [51], 2002b [50]	Individual RCT Netherlands	n=101 **Intervention 2** 64,1 (±15,0) ♀ 62% **Intervention 2** 69 (±9,8) ♀ 52% **Control** 64,5 (±9,7) ♀ 58%	BI	**Intervention 1 (IG-A)** UL training group (*n*=33) Median BI 5 (IQR 3-7) **Intervention 2 (IG-L)** LL training (*n*=31), Median BI 6 (IQR 3-8) **Control** Splint control group (*n*=37), Median BI 5.5 (IQR 3-7)	PTs and OTs	BI, FAC, ARAT, 10MWT, SIP, NHP, FAI	UL training group had significantly higher ARAT than the splint control group post-intervention. LL training group had significantly higher BI, FAC, walking speed and ARAT than the splint control group post-intervention. No significant differences in all outcomes were seen between groups from 6 months onwards up until the 12-month follow-up. ADL 6 weeks: IG-L: 13(8.75-19); IG-A: 10(5-13); *p*< 0.05) FAC: IG-L: 3(2-4); IG-A: 2(1-3); *p*<0.05 ARAT: IG-A: 3 (0-34), CG: 0 (0-1); *p*<0.05
Lincoln et al., 1999 [53]	Individual RCT UK	*n*=282 **Intervention** 73(64-80) ♀ 53% **Control 1** 73 (65-91) ♀ 46% **Control 2** 73 (66-80) ♀ 48%	BI^	**Intervention** Qualified PT group (*n*=94) Median BI 6 (IQR 3-9) **Control 1** PTA group (*n*=93) Median BI 6 (IQR 4-8) **Control 2** Standard PT group (*n*=95) Median BI 7 (IQR 3-9)	PTs/ PTAs	RMA- arm scale, ARAT, THPT, grip strength, mAS, BI, MCA	No differences between the groups across all outcomes.
Shao et al., 2023 [54]	Individual RCT China	*n*=139 **Intervention** 64.56 (±7.08) ♀ 23% **Control** 65.72 (±5.95) ♀ 40%	NIHSS	**Intervention** Strength training group (*n*=69) Median NIHSS 16.25 (±3.69) **Control** Usual physiotherapy group (*n*=70) Median NIHSS 15.97 (±3.30)	Not further defined	BBS, 6MWT, mBI, max. muscle strength	There are significant between-group differences for BBS (adjusted: 40.30±0.75 vs. 33.47±0.74; mean difference (95% CI) 6.83 (4.71 8.94); ɳ^2^= 0.24; *p* <0.001) and 6MWT (adjusted: 196.82±3.48 vs. 146.45±3.45, mean difference (95% CI) 50.32 (40.58 60.05); ɳ^2=^ 0.45; *p* <0.001). The comparison of the gain of muscle strength of hemiplegic limbs was in favour of the intervention group (*p*=0,01).

*Abbreviations:* *BBA* Brunel Balance Assessment, *BLS* Burke Lateropulsion Scale, *ARAT* Action research Arm Test, *aROM* Active Range of motion *BBA*, Brunel Balance Assessment, *BBS* Berg Balance Scale, *BDNF* Biomarker Brain-derived neurotrophic factor – Biomarker, *BI* Barthel Index, *BWS* Body weight supported, *CBS* Catherine Borgego Scale, *CG* Control group, *CI* Confidence Interval, *CSI* Caregiver Strain Index, *EQ-5D 5*L EuroQol – 5 Dimension – 5 Level, *FAC* Functional Ambulation Classification, *FAI* Frenchay activities Index, *FES* Functional electrical stimulation, *FIM* Functional independence measure, *FMA* Fugl-Meyer Assessment, *FMA LL* Fugl-Meyer Assessment lower extremity, *FMA UE* Fugl-Meyer Assessment upper extremity, *fMRI* Functional magnetic resonance images, *GHQ-28* General Health Questionnaire 28, *HADS* Hospital anxiety and depression scale, *HTSD* head-tracking sensor data, *ICIQ-SF* International Consultation on Incontinence Questionnaire-Short Form, *IG* Intervention group, *K-mBi* Korean Modified Barthel Index, *K-MMSE* Korean Minimental State Exam, *LBT* Line bisection Test, *mBI* Modified Barthel Index, *MAL* Motor Activity Log, *MAS* Modified Ashworth scale, *mBI* Modified Barthel Index, *MI* Motricity Index, *MAS* Motor-Assessment-Scale, *MMT* Manual muscle testing, *MoCA* Montreal Cognitive Asessment, *MRCS* Medical Research Council Scale, *mRS* Modified Rankin Scale, *MVPT-V* Motor-Free Visual Perception Test Vertical, *NHP* Nine-hole peg test, *NEADL* Nottingham extended Activities of daily living scale, *NIHSS* National Institute of Health Stroke Scale, *NMES* neuromuscular electrostimulation, *PASS* Postural Assessment Scale for Stroke, *OBASS* Overactive Bladder Symptom Score, *OT* Occupational Therapist, *PHQ-9* Patient Health Questionnaire, *PT* Physiotherapist, *PTA* Physiotherapy Assistend, *RCPM* Raven´s Coloured Progressive Matrices, *RMI* Rivermead Mobility Index, *rs fMRI* Resting state functional magnetic resonance imaging, *SADQ-10* Stroke Aphasia Depression Questionnaire-10, *SF-36* Medical Outcomes Short-Form 36, *SCT* Star cancelation Test, *SSS* Scandinavian Stroke Scale, *TCT* Trunk Control Test, *VAS* Visual analogue scale, *5MWT* 5 meter walking test, *6MWT* 6-minute Walk Test

### Participant characteristics

The participants’ characteristics are summarised in Table 2. Thirty studies reported on 2545 randomized patients with an average age of 67.25 years (SD ± 13.50). Two studies [49, 57] included participants in the hyperacute phase, four studies [52, 67, 71, 79] in the acute phase, 18 studies [53–56, 58, 60, 61, 64, 66, 68–70, 72–78] in the early subacute phase and three studies in the late subacute phase [59, 63, 65]. Three studies started in the chronic phase [46, 62, 70].

**Table 2:** Study demographics

	*n* = 30
Total number randomized	2545
Age, years °	67.25 (± 13.5)
Sex, *n* (%)	
Female	928 (36%)
Male	1617 (64%)
Stroke type, *n* (%) (*n* = 2500; 29 studies)	
Ischemic	974 (39%)
Hemorrhagic	1526 (61%)
Side of stroke, *n* (%) (*n* = 2459; 28 studies)	
Left	740 (30%)
Right	1719 (70%)
Time since stroke, *	
Hyperacute (≤ 24 h post-stroke)	2
Acute (> 24 h but ≤ 7days post-stroke)	4
Early subacute (> 7 days but ≤ 3 months post-stroke)	18
Late subacute (> 3 months but ≤ 6 months post-stroke)	3
Chronical (> 6 months)	3

⁰Source of calculated mean age [43]

*1month = 30 days

*Abbreviations:* h-hour

### Risk of bias

**Fig. 2:**
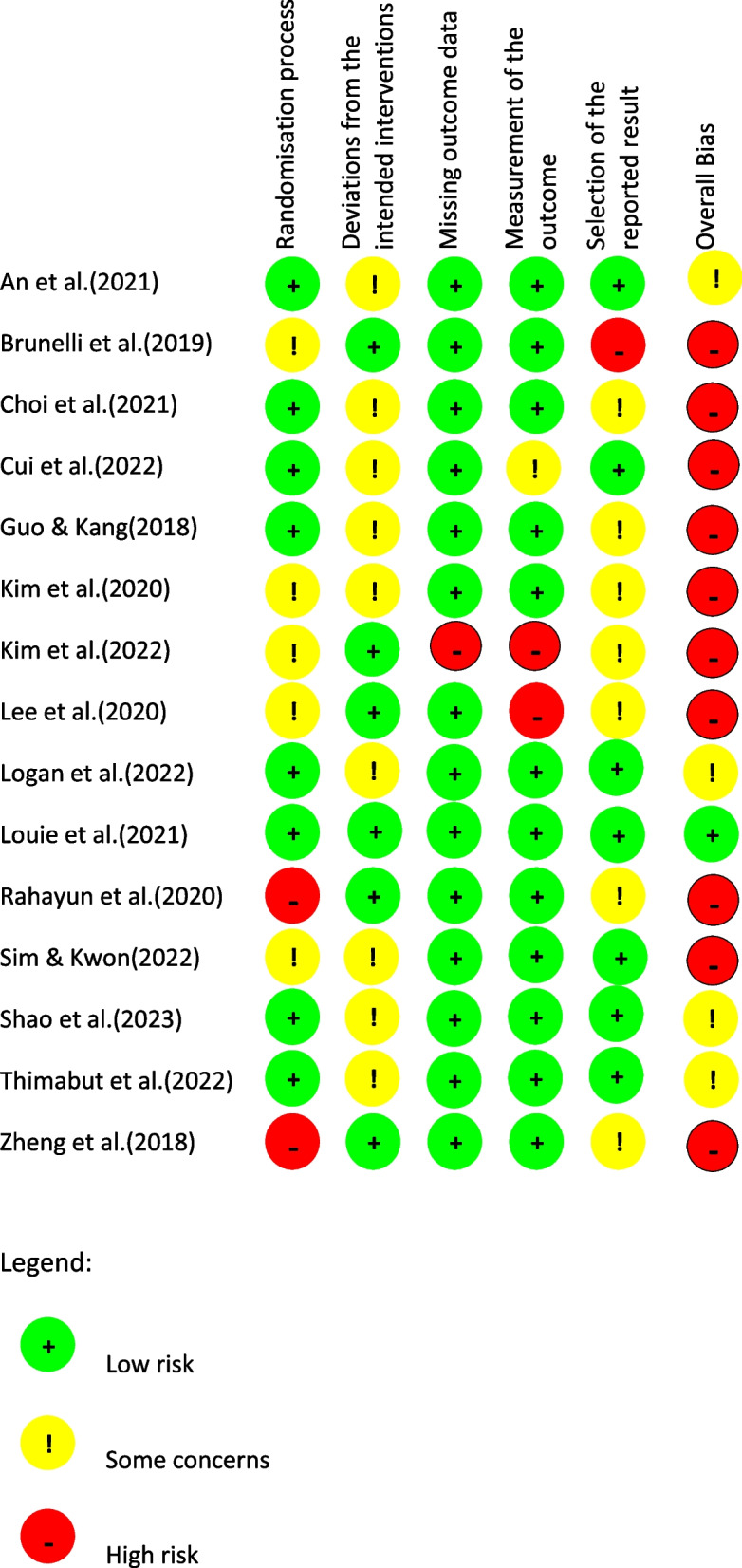
Risk of bias of individual domains for the updated studies. Randomisation process Deviations from the intended interventions Missing outcome data Measurement of the outcome Selection of the reported result

The results of the risk of bias assessment are shown in Figure 2. The individually randomised, parallel-group studies[46, 55, 60, 62, 63, 65, 66, 69, 71, 80], showed an overall high risk of bias. All ten studies raised some concerns or were rated as having a high risk of bias in the domains of deviation from intended interventions [46, 62, 65, 69, 80], randomisation process [62, 71] or missing or retrospective study registration [55]. According to the quality assessments of McGlinchey’s 15 studies, one had a low risk of bias [49], four studies had a serious risk [50–52, 72, 76, 79], and ten had a high risk of bias [53, 56–58, 61, 67, 68, 70, 73, 78].

### Types of interventions

The included studies used various types of interventions. Studies have used active rehabilitation interventions without technical support or devices, such as mirror therapy [60, 62, 69], additional upper or lower limb therapy [52], very early mobilization [49, 57], and interventions with specialized therapists in the Bobath or Carr and Shepard approaches [67, 68, 71]. Robotic-assisted body weight supported (BWS) treadmill gait training [61, 70, 76, 77], underwater gait training [46], BWS-supported overground gait training [55, 75], BWS-supported treadmill [58], or gait training and leg cycle ergometry [79] were used.

Three studies used electrical stimulation, such as functional electrical stimulation (FES) [66] and neuromuscular electrical stimulation (NMES) [65, 66]. Supportive devices for verticalization, such as a standing frame [73, 74] or robotic verticalization with the help of an Erigo [56], were used in 2 studies. One study involved digital practice with virtual reality [59], an elastic sling for the upper extremity [63], strength training [54], a whole-body tilt apparatus for postural training [64], differently qualified therapists [53], and thermal stimulation [78] as interventions. An overview of the interventions can be found in Table 1.

### Types of comparators

Interventions were compared to standard care [49, 57, 61, 66], delivered by physiotherapists (PTs) [53, 55, 56, 66, 70, 71, 74, 76–79], occupational therapists (OTs) [52, 60, 62, 78] or speech-language therapists (SLTs) [76, 79], as well as by nursing staff [57]. Only one study [67] used routine medication as a control intervention.

Standard care was often not described in detail. Information on the control intervention was incomplete in five studies [59, 60, 66, 71, 73] and was missing in two studies [24, 55].

### Therapy modalities

The study duration ranged from  7 days [71] to 20 weeks [50–52]. Therapy sessions were offered twice a day [55, 67, 72], daily [71], and most often five times per week [50–53, 58, 60, 61, 65, 66, 68, 70, 73, 76, 78, 81]. Session length differs per day from 10 min [79] to 120 min [57] up to statements that refer to the time spent in therapy alone [46, 55, 58, 68, 70, 72, 73, 76, 78]. The intensity of the interventions and what the control group performed in terms of content were inconsistently reported. An overview can be found in Table 2.

### Reporting on quality of evidence and dose

The 30 studies used 54 outcome measures. Those outcome measures were categorized by the ICF into body function (*n* = 26), activity (*n* = 23), and participation (*n* = 5). Supplementary material S4 provides an overview. Details on the GRADE criteria are reported in Supplementary material S5.

### Functional impairment in global early mobilization without electrical supportive devices within 24 h

Two studies [49, 57] compared early mobilization within 24 h to usual care referred to the outcome of independence. The quality of evidence was judged to be moderate due to concerns about inconsistency and imprecision. Inconsistency and imprecision in the results and their direction had been found and the threshold of 400 participants was not reached.

### Basic ADLs for neurodevelopmental interventions without electrical supportive devices

Neurodevelopmental interventions without electrical supportive devices compared to usual care for basic ADLs in severe stroke patients. The quality was judged with low quality of evidence due to concerns of inconsistency and imprecision [53, 71]. Wide confidence intervals (CG CI 95% 46.41 (37.77–55.04)); IG CI 95% (67 (58.99–75.94)) and appreciable benefits, and no difference between groups were found. Further on there were different directions of effects.

### Basic ADL in interventions with NMES

Two studies [65, 72] compared NMES to standardized upper limb therapy and sham NMES in basic ADL. The quality of evidence was judged to be low due to inconsistency and imprecision. Different control groups and varying ages in the population may affect the consistency. The required threshold of participants was not reached with 172 participants.

### Extended ADLs in interventions with verticalization support

Interventions with verticalization support compared to usual care in extended ADLs were found in two studies [73, 74]. The quality of evidence was judged to be low due to imprecisions and limitations in the design and implementation of the available studies. The threshold of 400 participants is not reached and the results are imprecise. Both studies showed no significant between-group differences when using a standing frame as verticalization support.

### Balance skills in neurodevelopmental interventions

The Berg Balance Scale (BBS) was used to assess balance skills in two RCTs [68, 71] using neurodevelopmental interventions compared to usual care and a conventional Bobath approach***.*** The quality of evidence was judged to be very low due to imprecisions, inconsistency of the results, and a high risk of bias in both studies due to serious methodological limitations. The optimal information size (OIS) was not reached and differences in population had been detected.

### Walking capacity in robotic-assisted gait training

Three studies [61, 75, 76] assessed walking capacity using the FAC comparing robotic-assisted gait training (RAGT) to conventional therapy and overground gait training. The quality of evidence was judged to be low. We had serious concerns about the inconsistency of the results based on the different reporting and directions of the effects. Regarding the OIS and the given effect sizes, we had serious concerns regarding imprecision.

### Motor function in robotic-assisted gait training

Three studies [61, 75, 76] compared RAGT to conventional therapy and overground gait training for assessing motor function in the lower limb with the Fugl-Mayer-Assessment of the lower extremity (FMA-LE). The quality of evidence was judged to be low due to concerns about inconsistency and imprecision. Serious concerns were raised regarding the OIS and the missing effect sizes as well as different directions of effect and slight differences in the intervention and control groups.

### Dexterity in highly intensive active interventions (without electric support)

Two studies investigating highly intensive active interventions compared to restriction and usual care for the dexterity of participants with severe stroke, measured using the Action Research Arm Test (ARAT), were judged low-quality evidence. This is due to some concerns about the risk of bias and the number of participants and the authors’ overall assessment, inconsistency in the results and their direction had been detected as well as not researching the threshold of 400 participants and imprecise results [52, 53].

The following outcomes were each examined in one study, and therefore no reliable conclusions can be drawn regarding their effects: spasticity in a staged rehabilitation intervention [67], neglect in digital training with virtual reality [59], and bimanual mirror therapy [62], cognitive function in robotic verticalization [81], sensorimotor function in thermal stimulation [69], walking capacity in BWS overground gait training [55], walking capacity in BWS overground gait training [70], spatiotemporal gait parameters in underwater gait training [46], walking speed in RAGT [77], balance in four-channel FES [66] and in strength training off the non-hemiplegic lower extremity [54], upper extremity function in multijoint mirror therapy [60], independence in daily and social activities in leg cycle ergometer [79], functional mobility in the use of an Oswestry standing [73], muscle strength while using an elastic dynamic sling [63] and pusher syndrome in whole-body tilt apparatus [82].

## Discussion

The goal of this systematic review was to summarise the most recent research on physical therapy interventions and their dosage requirements for patients with severe stroke who are treated in hospitals or inpatient rehabilitation facilities. Thirty studies [46–79] were included, with 54 outcomes and various types of interventions. Despite being an update of a recent systematic review, the overall evidence remains insufficient. However, the evidence is not robust enough to determine the effect of physical therapy interventions for patients with severe stroke. Due to limitations in the design, inconsistencies in results, and subjective interpretations, much of the evidence has been rated as low quality. There were not enough individual studies to obtain trustworthy evidence for the outcomes considered.

### Robotic gait interventions

The review included six studies using robotic, BWS, treadmill, or overground training to undertake a form of gait training, such as robotic-assisted body weight supported (BWS) treadmill gait training and underwater gait training [46, 47, 57, 60, 66, 67]. Unexpectedly, there was poor-quality evidence for all gait therapy outcomes. A systematic review of the current guidelines showed that robotic gait interventions are now recommended [83]. Eight out of 11 guidelines included supporting RAGT, which was shown to improve walking speed, step length, and balance. Previously, in a meta-regression study, Moucheboeuf et al. (2020, [84]) showed no correlation between stroke severity and age, time since stroke, rehabilitation intensity, or treatment success. Stroke severity was measured using the FAC. In contrast, other studies have shown that the severity of paresis influences the ability to predict the recovery of walking ability [85]. However, severity was measured by the presence of hemiparesis or hemiplegia. The Cochrane Review on treadmill training and BWS for walking after stroke [86] rated walking speed with moderate evidence quality, while our rating was low. They included 26 studies, compared to 2 in our review, and found that patients able to walk at the start improved more than those who could not. These findings align with our results, despite differing stroke severity levels in the studies. The differences in outcomes may caused by the different search approaches used. The current Cochrane Review on Electromechanical-assisted training for walking after stroke [87] still highlights recent findings. In contrast to our findings, they showed high-quality evidence that the use of electromechanical devices increased the likelihood of walking independently at the end of the intervention phase for survivors of stroke. Studies with a focus on patients with severe stroke were included instead of being intervention-specific. Nevertheless, the authors of the guideline review recommend the use of the RAGT for people who would not otherwise perform gait training [83]. However, RAGT should not be used in place of conventional therapy [83].

### Usual care

It is possible to hypothesize that the intervention or control group can be as effective only as the underlying standard therapies are if standard therapy (usual care) is used. In most of the studies included the interventions were carried out in addition to standard care. According to the TiDieR checklist, many of the studies lack a description of their interventions. Regardless of the term “standard therapy”, “standard care”, etc., refer to the standard of care at the local institution. Usual care and control groups are still insufficiently reported in intervention studies. For example, an intervention study may be based on very good usual care, and intervention in the study, however poor, may achieve good results. The reverse may also be true. A recent systematic review [88] reignited the discussion on control intervention groups and highlighted the importance of the therapies on which study interventions are based. Although usual care may be referred to by various terms, e.g., rehabilitative therapy [57], standard intervention [71], or conventional therapy [61], precise details regarding the frequency, intensity, and methods of patient treatment are rarely recorded. Using tools like the TidieR checklist [39], facilitate accurate therapy descriptions [89, 90], but this issue exists beyond neurorehabilitation [91]. It is important to remember that each nation's standard of treatment is shaped by its healthcare system. In Germany, outpatient stroke therapy can involve specialized methods like Bobath or proprioceptive neuromuscular facilitation  (PNF), which allow for higher payments [92]. Although Bobath is not guideline-recommended, it is widely used in the UK, with 67% of clinicians employing it for people with stroke [93]. Its use is evident in our findings [53, 61, 68]. Studies on the effectiveness of the Bobath vary from ineffective [92] to inconclusive [94] to effective [95]. Clinicians seek evidence-based, practical research that reflects real-world rehabilitation [96], underscoring the need for more transparent reporting in future studies, particularly regarding comparison interventions.

### Therapy dimensions

The frequency and dosage of therapeutic interventions like Bobath or RAGT are often unclear. For example, there is no consensus on the number, duration, timing, or appropriate patient profile for RAGT [83]. A Cochrane review indicates that treadmill gait training may be more beneficial in the first three months post-stroke than in the chronic phase, but the precise dosage remains uncertain [86]. The review also found no significant increase in walking speed for dependent stroke survivors at treatment onset (95% CI [− 0.06 to 0.03]; *P* = 0.52) [86]. A Cochrane review indicated that increased therapy duration generally improves outcomes, particularly for lower extremity functional impairments and ADLs [97].

One study approached this issue by varying the intervention start time [49]. However, the functional mobility outcome of early mobilization after stroke showed moderate evidence that early activation had no advantage over the control group. These interventions both took place in the acute phase after stroke. In a large multicentre study, 75% of all patients were mobilized within 18 h [49], but earlier mobilization was linked to higher mortality when the mRS score was evaluated (adjusted odds ratio (OR) 0.73, 95% CI [0.59 to 0.90]; *p* = 0.004). A recent systematic review supports starting mobilization 24 h post-stroke [98]. Although there was some evidence that patients with severe stroke and intracerebral hemorrhage would have worse outcomes with very early mobilization, these differences were not statistically significant (*p* > 0.05). Other RCTs [99, 100] with patients with mild to moderate strokes also indicated that more intervention did not necessarily lead to better outcomes (daily amount per person IG: 31 min (16.5–50.5 min); CG: 10 min (0–18 min)). These findings suggest that early mobilization within 24 h may be disadvantageous, and factors like intensity and duration, which depend on recovery phases, also affect outcomes [97]. Dromerick et al. [101] demonstrated positive effects in the subacute phase post-stroke, with a treatment window of two and three months and a daily intensity of 2 h. Of the included studies, only three [50–52, 61, 76] provided more therapy, and one [66] met Dromerick et al. [101] 600 min/week threshold. A systematic review reported a 240% increase in usual rehabilitation aimed at reducing ADL limitations [102], though it did not account for stroke severity. The interventions in weekly duration and total therapy amount are shown in Table 2.

### Limitations

A limitation of this review was restricting the language to German and English. To address this, we searched multiple databases using a broad strategy to identify RCTs for severe stroke treatment. Our strict assessment of the high risk of bias using the RoB 2 tool ensured consistency but may have contributed to heterogeneous evaluations among different authors.

Our given cut-off score for stroke severity may have excluded studies using different measures to define severe stroke [18, 103]. However, broad inclusion criteria resulted in a diverse range of studies with populations that were difficult to compare. The BI and mBI were most commonly used (twelve times in all studies) to define stroke severity. The heterogeneity in defining severe stroke may change with the adoption of Stroke Recovery and Rehabilitation Roundtable Taskforce recommendations [5], which suggest using the NIHSS score. Yet, no studies before 2018 reported this assessment. This could lead to the large heterogeneity of the included studies. Other reasons for this heterogeneity were also a high rated risk of bias, generally small sample size of included studies, and large and heterogeneous volume of outcome data. We did not include outpatient or home-based settings because we wanted to focus on the early health care of patients with severe stroke.

### Future research

Future research should: (a) address, how to assess stroke severity [5] and especially during baseline assessments [5]; (b) consider detailed reporting of interventions and control intervention as well as usual care [104, 105] including the amount of dosage according to the reporting guidelines and (c) studies should include follow-ups to assess long-term outcomes.

The interventions identified in this review are present in clinical practice, but certainty of their effectiveness is lacking for daily application. (d) A structured recording [42], would help accurately describe both the intervention and standard care, improving study comparability, certainty, and theory–practice transfer.

Investigating whether optimal, timely, and targeted therapy can reduce long-term costs is essential.

## Conclusion

This systematic review revealed mostly low- and moderate-quality evidence related to physical therapy interventions for patients with severe stroke. Although this is an update of an existing systematic review, there is still insufficient and little evidence to support the effectiveness of physical therapies in an inpatient setting. Compared to those of patients who had mild or moderate stroke, the results of the interventions in these studies had varying quality of evidence.

The included interventions reflected daily clinical practice. Until now, this certainty of evidence has been hindered by heterogeneous study populations and control groups that are difficult to compare. This has prevented us from drawing any practical conclusions. More research is needed. Certainty can be gained when there are more comparable interventions through a better description of the interventions, through a comparable control group, and through a clear description of the severity of stroke that has been studied. Better transparency will allow for better comparability between studies and their respective outcomes. The analysis of the therapy dimension of an intervention can be a key component in explaining study outcomes for patients. Here, in particular, it is interesting to take a closer look at which intervention is effective for which degree of severity of stroke and time post-stroke so that it does not turn into a watering can principle.

There is a need for additional high-quality studies in the early to late subacute phase that systematically articulate intervention doses from a multidimensional perspective in motor stroke recovery. This requires the implementation of the recommendation of stroke recovery and rehabilitation roundtables for the use of the NIHSS score as an assessment of stroke severity [5, 106].

## Supplementary Information

Supplementary material 1: S1. Search strategy example. S2. Reasons for excluded studies. S3. Funding sources of the included studies. S4. Outcomes according to ICF domains, S5. Grade Judgement

## Abbreviations

ADL: Activities of daily living
ARAT: Action Research Arm Test
BBS: Berg Balance Scale
BI: Barthel Index
BWS: Body weight support
CENTRAL: Cochrane Central Register of Controlled Trials
CI: Confidence interval
CINHAL: Cumulative Index to Nursing and Allied Health Literature
cRCT: Cluster randomized controlled trial
DALYs: Disability-adjusted life years
DORIS: Web of Science, Database of Research in Stroke
EMBASE: Excerpta Medica Database
FAC: Functional Ambulatory Categories
FES: Functional electrical stimulation
FIM: Functional Independence Measure
FMA: Fugl-Meyer-Assessment
FMA-UL: Fugl-Meyer-Assessment Upper Limb
FMA-LE: Fugl-Meyer-Assessment Lower Extremity
GRADE: Grading of Recommendations, Assessment, Development and Evaluation
ICF: International Classification of Functioning, Disability and Health
ICTRP: International Trials Registry Platform
IQR: Interquartile range
MAL: Motor Activity Log
MAS: Modified Ashworth Scale
MEDLINE: Medical Literature Analysis and Retrieval System Online
mRS: Modified Rankin Scale
NIHSS: National Institute of Health Stroke Scale
NMES: Neuromuscular electrical stimulation therapy
OIS: Optimal information size
OR: Odds ratio
OT: Occupational therapist
PEDro: Physiotherapy Evidence Database
PNF: Proprioceptive neuromuscular facilitation
PRR: Proportion recovery rule
PT: Physiotherapist
RAGT: Robotic-assisted gait therapy
RCT: Randomised controlled trial
RMA: Rivermead Motor Assessment
RoB2: Risk of Bias Tool 2
SLT: Speech-language therapist
TiDieR: Template for Intervention Description and Replication
UK: United Kingdom
