# Supplementary material for: Effects of physical therapy modalities for motor function, functional recovery, and post-stroke complications in patients with severe stroke: a systematic review update
Source: Syst Rev. 2024 Oct 28;13:270. doi: 10.1186/s13643-024-02676-0 (PMC11520505; doi:10.1186/s13643-024-02676-0)
Supplement: Supplementary file 1 — Supplementary material 1: S1. Search strategy example. S2. Reasons for excluded studies. S3. Funding sources of the included studies. S4. Outcomes according to ICF domains, S5. Grade Judgement [file 13643_2024_2676_MOESM1_ESM.docx]

**Supplementary material**

**Supplement S1 – Search strategy example**

**Search Strategy MEDLINE via Ovid according to the search strategy McGlinchey et al. (2020)**

1. exp Stroke/

2. severe stroke.mp.

3. stroke severit*.mp.

4. stroke disabilit*.mp.

5. exp Physical Therapy Modalities/

6. exp Occupational Therapy/

7. exp Nursing Care/

8. physical rehabilitation.mp.

9. exp Stroke Rehabilitation/

10. exp Patient Positioning/

11. exp Posture/

12. exp Exercise/

13. exp Exercise Therapy/

14. passive exercise.mp.

15. exp "Range of Motion, Articular"/

16. manual technique.mp.

17. active exercise.mp.

18. Resistance Training/

19. exp Muscle Stretching Exercises/

20. exp Electric Stimulation/

21. exp Electric Stimulation Therapy/

22. exp Wheelchairs/

23. seat?.mp.

24. exp "Equipment and Supplies"/

25. exp Teaching/

26. exp Education/

27. exp Motor Skills/

28. exp Movement/

29. motor function.mp.

30. motor recovery.mp.

31. exp "Recovery of Function"/

32. exp "Activities of Daily Living"/

33. functional independence.mp.

34. physical independence.mp.

35. complicatio*.mp.

36. exp Pain/

37. exp Contracture/

38. exp Pressure Ulcer/

39. exp Respiratory Tract Infections/

40. exp Urinary Tract Infections

41. Muscle Spasticity/

42. Venous Thrombosis/

43. exp Pulmonary Embolism/

44. exp Accidental Falls/

45. exp Fatigue/

46. exp Depression/

47. 1 or 2 or 3 or 4

48. 5 or 6 or 7 or 8 or 9 or 10 or 11 or 12 or 13 or 14 or 15 or 16 or 17 or 18 or 19 or 20 or 21 or 22 or

23 or 24 or 25 or 26

49. 27 or 28 or 29 or 30

50. 31 or 32 or 33 or 34

51. 35 or 36 or 37 or 38 or 39 or 40 or 41 or 42 or 43 or 44 or 45 or 46

52. 47 and 48 and 49

53. 47 and 48 and 50

54. 47 and 48 and 51

55. limit 52 to ("all adult (19 plus years)" and randomized controlled trial)

56. limit 53 to ("all adult (19 plus years)" and randomized controlled trial)

57. limit 54 to ("all adult (19 plus years)" and randomized controlled trial)

**Search Strategy CENTRAL**

#1 MeSH descriptor: [Stroke] explode all trees

#2 (severe stroke):ti,ab,kw

#3 (stroke disability):ti,ab,kw

#4 (stroke severity):ti,ab,kw

#5 MeSH descriptor: [Physical Therapy Modalities] explode all trees

#6 MeSH descriptor: [Occupational Therapy] explode all trees

#7 MeSH descriptor: [Nursing Care] explode all trees

#8 (physical rehabilitation):ti,ab,kw

#9 MeSH descriptor: [Stroke Rehabilitation] explode all trees

#10 MeSH descriptor: [Patient Positioning] explode all trees

#11 MeSH descriptor: [Posture] explode all trees

#12 MeSH descriptor: [Exercise] explode all trees

#13 MeSH descriptor: [Exercise Therapy] explode all trees

#14 (passive erxercise):ti,ab,kw

#15 MeSH descriptor: [Range of Motion, Articular] explode all trees

#16 (manual techniques):ti,ab,kw

#17 (active exercise):ti,ab,kw

#18 MeSH descriptor: [Resistance Training] explode all trees

#19 MeSH descriptor: [Muscle Stretching Exercises] explode all trees

#20 MeSH descriptor: [Electric Stimulation] explode all trees

#21 MeSH descriptor: [Electric Stimulation Therapy] explode all trees

#22 MeSH descriptor: [Wheelchairs] explode all trees

#23 (seat?):ti,ab,kw

#24 MeSH descriptor: [Equipment and Supplies] explode all trees

#25 MeSH descriptor: [Teaching] explode all trees

#26 MeSH descriptor: [Education] explode all trees

#27 MeSH descriptor: [Motor Skills] explode all trees

#28 MeSH descriptor: [Movement] explode all trees

#29 (motor function):ti,ab,kw

#30 (motor recovery):ti,ab,kw

#31 MeSH descriptor: [Recovery of Function] explode all trees

#32 MeSH descriptor: [Activities of Daily Living] explode all trees

#33 (functional independence):ti,ab,kw

#34 (physical independence):ti,ab,kw

#35 (complicatio*):ti,ab,kw

#36 MeSH descriptor: [Pain] explode all trees

#37 MeSH descriptor: [Contracture] explode all trees

#38 MeSH descriptor: [Pressure Ulcer] explode all trees

#39 MeSH descriptor: [Respiratory Tract Infections] explode all trees

#40 MeSH descriptor: [Urinary Tract Infections] explode all trees

#41 MeSH descriptor: [Muscle Spasticity] explode all trees

#42 MeSH descriptor: [Venous Thrombosis] explode all trees

#43 MeSH descriptor: [Pulmonary Embolism] explode all trees

#44 MeSH descriptor: [Accidental Falls] explode all trees

#45 MeSH descriptor: [Fatigue] explode all trees

#46 MeSH descriptor: [Depression] explode all trees

#47 #1 OR #2 OR #3 OR #4

#48 #5 OR #6 OR #7 OR #8 OR #9 OR #10 OR #11 OR #12 OR #13 OR #14 OR #15 OR #16 OR #17 OR #18 OR #19 OR #20 OR #21 OR #22 OR #23 OR #24 OR #25 OR #26

#49 #27 OR #28 OR #29 OR #30

#50 #31 OR #32 OR #33 OR #34

#51 #35 OR #36 OR #37 OR #38 OR #39 OR #40 OR #41 OR #42 OR #43 OR #44 OR #45 OR #46

#52 #47 AND #48 AND #49

#53 #47 AND #48 AND #50

#54 #47 AND #48 AND #51

#55 #52 with Publication Year from 2021 to 2023, with Cochrane Library publication date Between Apr 2021 and Mar 2023, in Trials

#56 #53 with Publication Year from 2021 to 2023, with Cochrane Library publication date Between Apr 2021 and Mar 2023, in Trials

#57 #54 with Publication Year from 2021 to 2023, with Cochrane Library publication date Between Apr 2021 and Mar 2023, in Trials

1. **Search strategy for CINHAL**(MH "Stroke+") OR TI severe stroke OR AB severe stroke OR TI stroke disability OR AB stroke disability OR TI stroke severity OR AB stroke severity
2. TX Physical Therapy Modalities OR (MH "Occupational Therapy+") OR MH nursing care OR TI physical rehabilitation OR AB physical rehabilitation
3. (MH "Rehabilitation+") AND TX stroke rehabilitation
4. (MH "Patient Positioning+")
5. (MH "Posture+")
6. (MH "Exercise+")
7. (MH "Therapeutic Exercise+")
8. TI passive exercise OR AB passive exercise
9. (MM "Range of Motion")
10. TI manual techniques OR AB manual techniques OR TI active exercise OR AB active exercise
11. (MM "Resistance Training")
12. (MH "Stretching")
13. (MH "Electric Stimulation+")
14. (MH "Wheelchairs+")
15. TI seat* OR AB seat*
16. (MH "Equipment and Supplies+")
17. (MH "Teaching+")
18. (MH "Education+")
19. (S2 OR S3 OR S4 OR S5 OR S6 OR S7 OR S8 OR S9 OR S10 OR S11 OR S12 OR S13 OR S14 OR S15 OR S16 OR S17 OR S18)
20. (MH "Motor Skills+")
21. (MH "Movement+")
22. TI motor function OR AB motor function
23. TI motor recovery OR AB motor recovery
24. (S20 OR S21 OR S22 OR S23)
25. (MH "Recovery+")
26. (MH "Activities of Daily Living+")
27. TI functional independence OR AB functional independence
28. TI physical independence OR AB physical independence
29. (S25 OR S26 OR S27 OR S28)
30. TI complicatio* OR AB complicatio*
31. (MH "Pain+")
32. (MH "Contracture+")
33. (MH "Pressure Ulcer+")
34. (MH "Respiratory Tract Infections+")
35. (MH "Urinary Tract Infections+")
36. (MM "Muscle Spasticity")
37. (MH "Venous Thrombosis+")
38. (MH "Pulmonary Embolism")
39. (MH "Accidental Falls")
40. (MH "Fatigue+")
41. (MH "Depression+")
42. (S30 OR S31 OR S32 OR S33 OR S34 OR S35 OR S36 OR S37 OR S38 OR S39 OR S40 OR S41)
43. S1 AND S19 AND S24 135 04/21-03/23, CT, RCT, engl
44. S1 AND S19 AND S29 68 04/21-03/23, CT, RCT, engl
45. S1 AND S19 AND S42 40 04/21-03/23, CT, RCT, engl  **Limiters** - Published Date: 20210401-20230331; Publication Type: Clinical Trial, Randomized Controlled Trial

**Search strategy for Web of Science**

1: ((((((TS=(stroke)) OR TI=(severe stroke)) OR AB=(severe stroke)) OR AB=(stroke severit*)) AND TI=(stroke severit*)) OR TI=(stroke disabili*)) OR AB=(stroke disabili*)

2: ((((((((((((((((((((((((((((TS=(Physical Therapy Modalities)) OR TS=(Occupational Therapy)) OR TS=(Nursing Care))) OR TI=(physical rehabilitation)) OR AB=(physical rehabilitation)) OR TS=(stroke rehabilitation)) OR TS=(patient positioning)) OR TS=(posture)) OR TS=(exercise)) OR TS=(exercise therapy))) OR TI=(passive exercise)) OR AB=(passive exercise)) OR TS=(Range of Motion, Articular)) OR TI=(manual technoque)) OR AB=(manual technoque)) OR AB=(active exercise)) OR TI=(active exercise)) OR TS=(restistance training)) OR TS=(muscle stretching exercise)) OR TS=(electric stimulation)) OR TS=(electric stimulation therapy)) OR TS=(wheelchairs)) OR TI=(seat?)) OR AB=( seat?)) OR TS=(equipment and supplies)) OR TS=(teaching)) OR TS=(education)

3: (((((TS=(motor skills)) OR TS=(Movement)) OR TI=(motor function)) OR AB=(motor function)) OR AB=(motor recovery)) OR AB=( motor recovery)

4: (((((TS=(recovery of function)) OR TS=(activities of daily living)) OR TI=(functional independence)) OR AB=(functional independence)) OR AB=(physical independence)) OR TI=(physical independence)

5: ((((((((((((TI=(complicatio*)) OR AB=(complicatio*)) OR TS=(pain)) OR TS=(contracture)) OR TS=(pressure ulcer)) OR TS=(respiratory tract infections)) OR TS=(urinary tract infections)) OR TS=(muscle spasticity)) OR TS=(venous thrombosis)) OR TS=(pulmonary embolism)) OR TS=(accidental falls)) OR TS=(fatigue)) OR TS=(depression)

6: #1 AND #2 AND #3 Timespan: 2021-04-01 to 2023-03-31

7: #1 AND #2 AND #4 Timespan: 2021-04-01 to 2023-03-31

8: #1 AND #2 AND #5 Timespan: 2021-04-01 to 2023-03-31

9: (#1 AND #2 AND #4) AND ALL=(trial* OR RCT) Timespan: 2021-04-01 to 2023-03-31

10: (#1 AND #2 AND #5) AND ALL=(trial* OR RCT) Timespan: 2021-04-01 to 2023-03-31

11: (#1 AND #2 AND #3) AND ALL=(trial* OR RCT) Timespan: 2021-04-01 to 2023-03-31

**Search strategy for Embase via Epistemonikos**

(advanced_title_en:((advanced_title_en:(stroke) OR advanced_abstract_en:(stroke)) OR (advanced_title_en:(severe stroke) OR advanced_abstract_en:(severe stroke)) OR (advanced_title_en:(stroke disability) OR advanced_abstract_en:(stroke disability)) OR (advanced_title_en:(stroke severity) OR advanced_abstract_en:(stroke severity))) OR advanced_abstract_en:((advanced_title_en:(stroke) OR advanced_abstract_en:(stroke)) OR (advanced_title_en:(severe stroke) OR advanced_abstract_en:(severe stroke)) OR (advanced_title_en:(stroke disability) OR advanced_abstract_en:(stroke disability)) OR (advanced_title_en:(stroke severity) OR advanced_abstract_en:(stroke severity)))) AND (advanced_title_en:((advanced_title_en:(Physical Therapy Modalities) OR advanced_abstract_en:(Physical Therapy Modalities)) OR (advanced_title_en:(Occupational Therapy) OR advanced_abstract_en:(Occupational Therapy)) OR (advanced_title_en:(Nursing Care) OR advanced_abstract_en:(Nursing Care)) OR (advanced_title_en:(physical rehabilitation) OR advanced_abstract_en:(physical rehabilitation)) OR (advanced_title_en:(stroke rehabilitation) OR advanced_abstract_en:(stroke rehabilitation)) OR (advanced_title_en:(patient positioning) OR advanced_abstract_en:(patient positioning)) OR (advanced_title_en:(posture) OR advanced_abstract_en:(posture)) OR (advanced_title_en:(exercise) OR advanced_abstract_en:(exercise)) OR (advanced_title_en:(exercise therapy) OR advanced_abstract_en:(exercise therapy)) OR (advanced_title_en:(passive exercise) OR advanced_abstract_en:(passive exercise)) OR (advanced_title_en:(range of motion) OR advanced_abstract_en:(range of motion)) OR (advanced_title_en:(manual techniques) OR advanced_abstract_en:(manual techniques)) OR (advanced_title_en:(active exercise) OR advanced_abstract_en:(active exercise)) OR (advanced_title_en:(restistance training) OR advanced_abstract_en:(restistance training)) OR (advanced_title_en:(Muscle Stretching Exercises) OR advanced_abstract_en:(Muscle Stretching Exercises)) OR (advanced_title_en:(Electric Stimulation) OR advanced_abstract_en:(Electric Stimulation)) OR (advanced_title_en:(Wheelchairs) OR advanced_abstract_en:(Wheelchairs)) OR (advanced_title_en:(seat?) OR advanced_abstract_en:(seat?)) OR (advanced_title_en:(Equipment AND Supplies) OR advanced_abstract_en:(Equipment AND Supplies)) OR (advanced_title_en:(Teaching) OR advanced_abstract_en:(Teaching)) OR (advanced_title_en:(Eduction) OR advanced_abstract_en:(Eduction))) OR advanced_abstract_en:((advanced_title_en:(Physical Therapy Modalities) OR advanced_abstract_en:(Physical Therapy Modalities)) OR (advanced_title_en:(Occupational Therapy) OR advanced_abstract_en:(Occupational Therapy)) OR (advanced_title_en:(Nursing Care) OR advanced_abstract_en:(Nursing Care)) OR (advanced_title_en:(physical rehabilitation) OR advanced_abstract_en:(physical rehabilitation)) OR (advanced_title_en:(stroke rehabilitation) OR advanced_abstract_en:(stroke rehabilitation)) OR (advanced_title_en:(patient positioning) OR advanced_abstract_en:(patient positioning)) OR (advanced_title_en:(posture) OR advanced_abstract_en:(posture)) OR (advanced_title_en:(exercise) OR advanced_abstract_en:(exercise)) OR (advanced_title_en:(exercise therapy) OR advanced_abstract_en:(exercise therapy)) OR (advanced_title_en:(passive exercise) OR advanced_abstract_en:(passive exercise)) OR (advanced_title_en:(range of motion) OR advanced_abstract_en:(range of motion)) OR (advanced_title_en:(manual techniques) OR advanced_abstract_en:(manual techniques)) OR (advanced_title_en:(active exercise) OR advanced_abstract_en:(active exercise)) OR (advanced_title_en:(restistance training) OR advanced_abstract_en:(restistance training)) OR (advanced_title_en:(Muscle Stretching Exercises) OR advanced_abstract_en:(Muscle Stretching Exercises)) OR (advanced_title_en:(Electric Stimulation) OR advanced_abstract_en:(Electric Stimulation)) OR (advanced_title_en:(Wheelchairs) OR advanced_abstract_en:(Wheelchairs)) OR (advanced_title_en:(seat?) OR advanced_abstract_en:(seat?)) OR (advanced_title_en:(Equipment AND Supplies) OR advanced_abstract_en:(Equipment AND Supplies)) OR (advanced_title_en:(Teaching) OR advanced_abstract_en:(Teaching)) OR (advanced_title_en:(Eduction) OR advanced_abstract_en:(Eduction)))) AND (advanced_title_en:((advanced_title_en:(Motor Skills) OR advanced_abstract_en:(Motor Skills)) OR (advanced_title_en:(Movement) OR advanced_abstract_en:(Movement)) OR (advanced_title_en:(motor function) OR advanced_abstract_en:(motor function)) OR (advanced_title_en:(motor recovery) OR advanced_abstract_en:(motor recovery))) OR advanced_abstract_en:((advanced_title_en:(Motor Skills) OR advanced_abstract_en:(Motor Skills)) OR (advanced_title_en:(Movement) OR advanced_abstract_en:(Movement)) OR (advanced_title_en:(motor function) OR advanced_abstract_en:(motor function)) OR (advanced_title_en:(motor recovery) OR advanced_abstract_en:(motor recovery)))) [Filters: protocol=no, classification=primary-study, min_year=2021, max_year=2023]

**Search Strategy PEDro**

Abstract & tile: severe stroke

Subdisciplin: Nuerology

Method: clinical trail

Published science: 20201

New records added since: 2021

**Search strategy for DORIS**

Abstract & Title: severe stroke

Subdiscipline: Neurology

Method: clinical trial

Published since: 2021

**Supplement S2 – Reasons for excluded studies**

| Study | Exclusion reason |
| --- | --- |
| Adomavičiene A, Grigonyte A, Juocevičius A. Occupational therapy efficacy on upper-extremity functional outcomes after stroke. European Stroke Journal. Januar 2019;4:150. | wrong publication type |
| Ahn JY, Kim H, Park CB. Effects of Whole-Body Vibration on Upper Extremity Function and Grip Strength in Patients with Subacute Stroke: A Randomised Single-Blind Controlled Trial. Occupational therapy international. Januar 2019;2019:5820952. | stroke severity not defined |
| Alanbay E, Aras B, Kesikburun S, Kizilirmak S, Yasar E, Tan AK. Effectiveness of Suprascapular Nerve Pulsed Radiofrequency Treatment for Hemiplegic Shoulder Pain: A Randomized-Controlled Trial. Pain physician. Januar 2020;23(3):245–52. | Not severe |
| Alex and er J. Saeboglove therapy for severe upper limb disability and severe hand impairment after stroke (SUSHI). Januar 2019; Verfügbar unter: http://clinicaltrials.gov/ct2/show/NCT04007315 | ongoing |
| Alingh JF, Groen BE, Kamphuis JF, Geurts ACH, Weerdesteyn V. Task-specific training for improving propulsion symmetry and gait speed in people in the chronic phase after stroke: a proof-of-concept study. J Neuroeng Rehabil. 2021;18(1):69. | Wrong setting |
| Alisar DC, Ozen S, Sozay S. Effects of Bihemispheric Transcranial Direct Current Stimulation on Upper Extremity Function in Stroke Patients: A randomized Double-Blind Sham-Controlled Study. Journal of Stroke & Cerebrovascular Diseases. Januar 2020;29(1):N.PAG-N.PAG. | Not severe |
| Alwhaibi R, Mahmoud N, Basheer M, Zakaria H, Elzanaty M, Ragab W, u. a. Impact of somatosensory training on neural and functional recovery of lower extremity in patients with chronic stroke: a single blind controlled randomized trial. International journal of environmental research and public health. Januar 2021;18(2):1‐10. | Wrong setting |
| Ambrosini E, Peri E, Nava C, Longoni L, Monticone M, Pedrocchi A, u. a. A multimodal training with visual biofeedback in subacute stroke survivors: a randomized controlled trial. European Journal of Physical and Rehabilitation Medicine [Internet]. Februar 2020;56(1). Verfügbar unter: https://www.minervamedica.it/index2.php?show=R33Y2020N01A0024 | stroke severity not defined |
| Ambrosini E, Gasperini G, Zajc J, Immick N, Augsten A, Rossini M, u. a. A Robotic System with EMG-Triggered Functional Eletrical Stimulation for Restoring Arm Functions in Stroke Survivors. Neurorehabil Neural Repair. 2021;35(4):334–45. | stroke severity not defined |
| Ancona E, Quarenghi A, Simonini M, Saggini R, Mazzoleni S, De Tanti A, u. a. Effect of verticalization with Erigo® in the acute rehabilitation of severe acquired brain injury. Neurological sciences. Januar 2019;40(10):2073‐2080. | Not severe |
| Annino G, Alashram AR, Alghwiri AA, Romagnoli C, Messina G, Tancredi V, u. a. Effect of segmental muscle vibration on upper extremity functional ability poststroke: A randomized controlled trial. Medicine. Februar 2019;98(7):e14444–e14444. | stroke severity not defined |
| Antoniotti P, Veronelli L, Caronni A, Monti A, Aristidou E, Montesano M, u. a. No evidence of effectiveness of mirror therapy early after stroke: an assessor-blinded randomized controlled trial. Clinical rehabilitation. Januar 2019;33(5):885‐893. |  |
| Aprile I, Germanotta M, Cruciani A, Pecchioli C, Loreti S, Papadopoulou D, u. a. Poststroke shoulder pain in subacute patients and its correlation with upper limb recovery after robotic or conventional treatment: a secondary analysis of a multicenter randomized controlled trial. International journal of stroke [Internet]. Januar 2020; Verfügbar unter: https://www.cochranelibrary.com/central/doi/10.1002/central/CN-02138914/full | Not severe |
| Aprile I, Germanotta M, Cruciani A, Loreti S, Pecchioli C, Cecchi F, u. a. Upper Limb Robotic Rehabilitation After Stroke: A Multicenter, Randomized Clinical Trial. Journal of neurologic physical therapy : JNPT. Januar 2020;44(1):3–14. | stroke severity not defined |
| Araki S, Kawada M, Miyazaki T, Nakai Y, Takeshita Y, Matsuzawa Y, u. a. Effect of Functional Electrical Stimulation of the Gluteus Medius during Gait in Patients following a Stroke. BioMed Research International [Internet]. Januar 2020;2020. Verfügbar unter: https://www.embase.com/search/results?subaction=viewrecord&id=L2010225641&from=export | stroke severity not defined |
| Arnao V, Riolo M, Carduccio F, Tuttolomondo A, D’Amelio M, Brighina F, u. a. Effects of transcranial random noise stimulation combined with Graded Repetitive Arm Supplementary Program (GRASP) on motor rehabilitation of the upper limb in sub-acute ischemic stroke patients: a randomized pilot study. Journal of Neural Transmission. Januar 2019;126(12):1701–6. | Not severe |
| Arreola V, Ortega O, Álvarez-Berdugo D, Rofes L, Tomsen N, Cabib C, u. a. Effect of Transcutaneous Electrical Stimulation in Chronic Poststroke Patients with Oropharyngeal Dysphagia: 1-Year Results of a Randomized Controlled Trial. Neurorehabil Neural Repair. 2021;35(9):778–89. | Not severe |
| Arya KN, P, ian S, Sharma A, Kumar V, Kashyap VK. Interlimb coupling in poststroke rehabilitation: a pilot randomized controlled trial. Topics in Stroke Rehabilitation. Januar 2020;27(4):272–89. | Not severe |
| Arya KN, P, ian S, Kumar V. Effect of activity-based mirror therapy on lower limb motor-recovery and gait in stroke: A randomised controlled trial. Neuropsychological rehabilitation. Januar 2019;29(8):1193–210. | Not severe |
| Avelino PR, Nascimento LR, Ada L, de Menezes KKP, Teixeira-Salmela LF. Using a cane for one month does not improve walking or social participation in chronic stroke: An attention-controlled randomized trial. Clin Rehabil. 2021;35(11):1590–8. | Wrong setting |
| Aycock DM, Clark PC, Hayat MJ, Salazar LF, Eriksen MP. Stroke Counseling Intervention for Young Adult African Americans: A Randomized Controlled Trial. Nurs Res. 2023;72(2):83–92 | Wrong intervention |
| Bessa NPOS, Lima Filho BFD, Medeiros CSPD, Ribeiro TS, Campos TF, Cavalcanti FADC. Effects of exergames training on postural balance in patients who had a chronic stroke: Study protocol for a randomised controlled trial. BMJ Open [Internet]. Januar 2020;10(11). Verfügbar unter: https://www.embase.com/search/results?subaction=viewrecord&id=L633332685&from=export | Not severe, outpatient |
| Bilek F, Deniz G, Ercan Z, Cetisli Korkmaz N, Alkan G. The effect of additional neuromuscular electrical stimulation applied to erector spinae muscles on functional capacity, balance and mobility in post-stroke patients. NeuroRehabilitation. Januar 2020;47(2):181–9. | Not severe |
| Boasquevisque DS, Servinsckins L, de Paiva JPQ, Dos Santos DG, Soares P, Pires DS, u. a. Contralesional Cathodal Transcranial Direct Current Stimulation Does Not Enhance Upper Limb Function in Subacute Stroke: A Pilot Randomized Clinical Trial. Neural Plast. 2021;2021:8858394. | stroke severity not defined |
| Bolognini N, Russo C, Souza Carneiro MI, Nicotra A, Olgiati E, Sp, u. a. Bi-hemispheric transcranial direct current stimulation for upper-limb hemiparesis in acute stroke: a randomized, double-blind, sham-controlled trial. European Journal of Neurology. Januar 2020;27(12):2473–82. | Not severe |
| Bornheim S, Croisier JL, Maquet P, Kaux JF. Transcranial direct current stimulation associated with physical-therapy in acute stroke patients - A randomized, triple blind, sham-controlled study. Brain Stimulation. Januar 2020;13(2):329–36. | Not severe |
| Bovonsunthonchai S, Aung N, Hiengkaew V, Tretriluxana J. A randomized controlled trial of motor imagery combined with structured progressive circuit class therapy on gait in stroke survivors. Scientific reports. Januar 2020;10(1):6945. | Not severe |
| Bradley L, Hart BB, Mandana S, Flowers K, Riches M, Sanderson P. Electromyographic biofeedback for gait training after stroke. Clin Rehabil. Februar 1998;12(1):11–22. | Wrong setting |
| Broderick P, Horgan F, Blake C, Ehrensberger M, Simpson D, Monaghan K. Mirror therapy and treadmill training for patients with chronic stroke: a pilot randomized controlled trial. Topics in stroke rehabilitation. Januar 2019;26(3):163–72. | Not severe |
| Bunketorp-Käll L, Pekna M, Pekny M, Blomstr, Christian, Nilsson M. Effects of horse-riding therapy and rhythm and music-based therapy on functional mobility in late phase after stroke. NeuroRehabilitation. August 2019;45(4):483–92. | Not severe |
| Busk H, Skou ST, Lyckhage LF, Arens CH, Asgari N, Wienecke T. Neuromuscular Electric Stimulation in Addition to Exercise Therapy in Patients with Lower Extremity Paresis Due to Acute Ischemic Stroke. A proof-of-concept randomised controlled trial. J Stroke Cerebrovasc Dis. 2021;30(10):106050. | Not severe |
| C A, an S, Li̇vaneli̇oğlu A. Efficacy of Modified Constraint-Induced Movement Therapy for Lower Extremity in Patients with Stroke: Strength and Quality of Life Outcomes. Turkish Journal of Physiotherapy Rehabilitation. Januar 2019;30(1):23–32. | stroke severity not defined |
| Calabro RS, Accorinti M, Porcari B, Carioti L, Ciatto L, Billeri L, u. a. Does hand robotic rehabilitation improve motor function by rebalancing interhemispheric connectivity after chronic stroke? Encouraging data from a randomised-clinical-trial. Clinical neurophysiology : official journal of the International Federation of Clinical Neurophysiology. Januar 2019;130(5):767–80. | stroke severity not defined |
| Carpinella I, Lencioni T, Bowman T, Bertoni R, Turolla A, Ferrarin M, u. a. Effects of robot therapy on upper body kinematics and arm function in persons post stroke: a pilot randomized controlled trial. Journal of neuroengineering and rehabilitation. Januar 2020;17(1):10. | Not severe |
| Carrico C, Annichiarico N, Powell ES, Westgate PM, Sawaki L. Chronicity of Stroke Does Not Affect Outcomes of Somatosensory Stimulation Paired With Task-Oriented Motor Training: A Secondary Analysis of a Randomized Controlled Trial. Archives of Rehabilitation Research and Clinical Translation [Internet]. Januar 2019;1(1). Verfügbar unter: https://www.embase.com/search/results?subaction=viewrecord&id=L2001993186&from=export | stroke severity not defined |
| Cattagni T, Geiger M, Supiot A, de Mazancourt P, Pradon D, Zory R, u. a. A single session of anodal transcranial direct current stimulation applied over the affected primary motor cortex does not alter gait parameters in chronic stroke survivors. Neurophysiologie clinique = Clinical neurophysiology. September 2019;49(4):283–93. | stroke severity not defined |
| Cavalcanti Garcia L, Carmona Alcântara C, Lopes Santos G, Almeida Monção JV, Russo TL. Cryotherapy Reduces Muscle Spasticity But Does Not Affect Proprioception in Ischemic Stroke: A Randomized Sham-Controlled Crossover Study. American Journal of Physical Medicine & Rehabilitation. Januar 2019;98(1):51–7. | stroke severity not defined |
| Cha JH, Kim NH, Cha YJ. Effect of proprioceptive stimulation induced by footplate during center of pressure movement tracking training on the balance abilities of patients with chronic hemiplegic stroke: a randomized, controlled, pilot study. Topics in stroke rehabilitation. Januar 2020;27(1):38–43. | stroke severity not defined |
| Chaiyawat P, Kulkantrakorn K. Effectiveness of home rehabilitation program for ischemic stroke upon disability and quality of life: A randomized controlled trial. Clinical Neurology and Neurosurgery. September 2012;114(7):866–70. | Not severe |
| Chaiyawat P, Kulkantrakorn K. Randomized controlled trial of home rehabilitation for patients with ischemic stroke: impact upon disability and elderly depression. Psychogeriatrics. September 2012;12(3):193–9. | Not severe |
| Chatterjee K, Stockley RC, Lane S, Watkins C, Cottrell K, Ankers B, u. a. PULSE-I - Is rePetitive Upper Limb SEnsory stimulation early after stroke feasible and acceptable? A stratified single-blinded randomised controlled feasibility study. Trials. Januar 2019;20(1):N.PAG-N.PAG. | Not severe |
| Chen CC, Tang YC, Hsu MJ, Lo SK, Lin JH. Effects of the hybrid of neuromuscular electrical stimulation and noxious thermal stimulation on upper extremity motor recovery in patients with stroke: a randomized controlled trial. Topics in Stroke Rehabilitation. Januar 2019;26(1):66–72. | Wrong setting |
| Chen L, Xiong S, Liu Y, Lin M, Zhu L, Zhong R, u. a. Comparison of Motor Relearning Program versus Bobath Approach for Prevention of Poststroke Apathy: A Randomized Controlled Trial. Journal of Stroke & Cerebrovascular Diseases. März 2019;28(3):655–64. | Not severe |
| Chew E, Teo WP, Tang N, Ang KK, Ng YS, Zhou JH, u. a. Using Transcranial Direct Current Stimulation to Augment the Effect of Motor Imagery-Assisted Brain-Computer Interface Training in Chronic Stroke Patients—Cortical Reorganization Considerations. Frontiers in Neurology [Internet]. Januar 2020;11. Verfügbar unter: https://www.embase.com/search/results?subaction=viewrecord&id=L632797593&from=export | stroke severity not defined |
| Chilvers M, Cluff T, Kirton A, Hill M, Dukelow S. Protocol to assess the combined effect of robotic rehabilitation and transcranial direct current stimulation on proprioception in chronic stroke: A pilot trial. European Stroke Journal. Januar 2019;4:815. | wrong publication type |
| Chinembiri B, Ming Z, Kai S, Xiu Fang Z, Wei C. The fourier M2 robotic machine combined with occupational therapy on post-stroke upper limb function and independence-related quality of life: A randomized clinical trial. Topics in Stroke Rehabilitation. Januar 2021;28(1):1–18. | Not severe |
| Chiu EC, Chi FC, Chen PT. Investigation of the home-reablement program on rehabilitation outcomes for people with stroke: A pilot study. Medicine (Baltimore). 2021;100(26):e26515. | Wrong setting |
| Cho J, Lee E, Lee S. Effectiveness of mid-thoracic spine mobilization versus therapeutic exercise in patients with subacute stroke: A randomized clinical trial. Technology & Health Care. März 2019;27(2):149–58. | stroke severity not defined |
| Cho KH, Park SJ. Effects of joint mobilization and stretching on the range of motion for ankle joint and spatiotemporal gait variables in stroke patients. Journal of Stroke & Cerebrovascular Diseases. August 2020;29(8):N.PAG-N.PAG. | stroke severity not defined |
| Choi HS, Shin WS, Bang DH. Mirror therapy using gesture recognition for upper limb function, neck discomfort, and quality of life after chronic stroke: A single-blind randomized controlled trial. Medical Science Monitor. Januar 2019;25:3271–8. | stroke severity not defined |
| Choi JB, Jung YJ, Park JS. Comparison of 2 types of therapeutic exercise: jaw opening exercise and head lift exercise for dysphagic stroke: A pilot study. Medicine. Januar 2020;99(38):e22136. | stroke severity not defined |
| Choi Yoon-Hee, Kim Kyoung, Lee Sang-Yong, Cha Yong-Jun. Lower limb muscle activities and gain in balancing ability following two types of stair gait intervention in adult post-chronic stroke patients: A preliminary, randomized-controlled study. Turkish Journal of Physical Medicine & Rehabilitation (2587-1250). Januar 2020;66(1):17–23. | stroke severity not defined |
| Choi YH, Kim JD, Lee JH, Cha YJ. Walking and balance ability gain from two types of gait intervention in adult patients with chronic hemiplegic stroke: A pilot study. Assistive Technology. Juni 2019;31(2):112–5. | stroke severity not defined |
| Choi YH, Kim NH, Son SM, Cha YJ. Effects of Trunk Stabilization Exercise While Wearing a Pelvic Compression Belt on Walking and Balancing Abilities in Patients With Stroke: An Assessor Blinded, Preliminary, Randomized, Controlled Study. American journal of physical medicine & rehabilitation. Januar 2020;99(11):1048–55. | stroke severity not defined |
| Chu K, Bu X, Sun Z, Wang Y, Feng W, Xiao L, u. a. Feasibility of a Nurse-Trained, Family Member-Delivered Rehabilitation Model for Disabled Stroke Patients in Rural Chongqing, China. Journal of stroke and cerebrovascular diseases : the official journal of National Stroke Association. Januar 2020;29(12):105382. | Not severe |
| Cinone N, Letizia S, Santoro L, Facciorusso S, Armiento R, Picelli A, u. a. Combined effects of isokinetic training and botulinum toxin type a on spastic equinus foot in patients with chronic stroke: A pilot, single-blind, randomized controlled trial. Toxins [Internet]. Januar 2019;11(4). Verfügbar unter: https://www.embase.com/search/results?subaction=viewrecord&id=L2001879327&from=export | Wrong intervention |
| Clark DJ, Rose DK, Butera KA, Hoisington B, DeMark L, Chatterjee SA, u. a. Rehabilitation with accurate adaptability walking tasks or steady state walking: A randomized clinical trial in adults post-stroke. Clin Rehabil. 2021;35(8):1196–206. | stroke severity not defined |
| Collett J, Fleming MK, Meester D, Al-Yahya E, Wade DT, Dennis A, u. a. Dual-task walking and automaticity after Stroke: Insights from a secondary analysis and imaging sub-study of a randomised controlled trial. Clin Rehabil. 2021;35(11):1599–610. | Not severe |
| Conforto AB, Luccas R, Menezes IS, Machado AG, Mello EA, Assis PS, u. a. Peripheral nerve stimulation to enhance upper limb motor function in stroke. Stroke [Internet]. Januar 2019;50. Verfügbar unter: https://www.embase.com/search/results?subaction=viewrecord&id=L628147233&from=export | Wrong publication type |
| Conroy SS, Wittenberg GF, Krebs HI, Zhan M, Bever CT, Whitall J. Robot-Assisted Arm Training in Chronic Stroke: Addition of Transition-to-Task Practice. Neurorehabilitation and Neural Repair. Januar 2019;33(9):751–61. | stroke severity not defined |
| Cordo P. Treatment of Chronic Stroke With AMES + EMG Biofeedback (AMES). Januar 2019; Verfügbar unter: http://clinicaltrials.gov/ct2/show/NCT01116544 | Publication date |
| Criekinge TV, Hallemans A, Herssens N, Lafosse C, Claes D, Hertogh WD, u. a. SWEAT2 Study: Effectiveness of Trunk Training on Gait and Trunk Kinematics After Stroke: A Randomized Controlled Trial. Physical Therapy. September 2020;100(9):1568–81. | Not severe |
| da Rosa Pinheiro DR, Cabeleira MEP, da Campo LA, Correa PS, Blauth AHEG, Cechetti F, u. a. Effects of aerobic cycling training on mobility and functionality of acute stroke subjects: A randomized clinical trial. NeuroRehabilitation. Januar 2021;48(1):39–47. | stroke severity not defined |
| Da Silva Rodrigues JC, Luvizutto GJ, Da Costa RDM, Prudente RA, Da Silva TR, De Souza JT, u. a. Influence of an exercise program on cardiac remodeling and functional capacity in patients with stroke (CRONuS trial): Study protocol for a randomized controlled trial. Trials [Internet]. Januar 2019;20(1). Verfügbar unter: https://www.embase.com/search/results?subaction=viewrecord&id=L627902211&from=export | Not severe, study protocol |
| Daly JJ, McCabe JP, Holcomb J, Monkiewicz M, Gansen J, Pundik S. Long-Dose Intensive Therapy Is Necessary for Strong, Clinically Significant, Upper Limb Functional Gains and Retained Gains in Severe/Moderate Chronic Stroke. Neurorehabilitation and Neural Repair. Januar 2019;33(7):523–37. | stroke severity not defined |
| Dawson J, Liu CY, Francisco GE, Cramer SC, Wolf SL, Dixit A, u. a. Vagus nerve stimulation paired with rehabilitation for upper limb motor function after ischaemic stroke (VNS-REHAB): a randomised, blinded, pivotal, device trial. Lancet. 2021;397(10284):1545–53. | stroke severity not defined |
| De Bruyn N, Saenen L, Thijs L, Van Gils A, Ceulemans E, Essers B, u. a. Sensorimotor vs. Motor Upper Limb Therapy for Patients With Motor and Somatosensory Deficits: A Randomized Controlled Trial in the Early Rehabilitation Phase After Stroke. Frontiers in Neurology [Internet]. Januar 2020;11. Verfügbar unter: https://www.embase.com/search/results?subaction=viewrecord&id=L633690376&from=export | stroke severity not defined |
| de Sousa DG, Harvey LA, Dorsch S, Varettas B, Jamieson S, Murphy A, u. a. Two weeks of intensive sit-to-stand training in addition to usual care improves sit-to-stand ability in people who are unable to stand up independently after stroke: a randomised trial. Journal of Physiotherapy (Elsevier). Juli 2019;65(3):152–8. | Not severe |
| De Souza JA, Corrêa JCF, Agnol LD, Dos Santos FR, Gomes MRP, Corrêa FI. Effects of transcranial direct current stimulation on the rehabilitation of painful shoulder following a stroke: Protocol for a randomized, controlled, double-blind, clinical trial. Trials [Internet]. Januar 2019;20(1). Verfügbar unter: https://www.embase.com/search/results?subaction=viewrecord&id=L626772228&from=export | Wrong intervention |
| Debreceni-Nagy A, Horvath J, Bajuszne Kovacs N, Fulop P, Jenei Z. The effect of low-intensity aerobic training on cognitive functions of severely deconditioned subacute and chronic stroke patients: a randomized, controlled pilot study. International Journal of Rehabilitation Research. Januar 2019;42(3):275–9. | Not severe |
| Dehem S, Gilliaux M, Stoquart G, Detrembleur C, Jacquemin G, Palumbo S, u. a. Effectiveness of upper-limb robotic-assisted therapy in the early rehabilitation phase after stroke: A single-blind, randomised, controlled trial. Annals of Physical and Rehabilitation Medicine. Januar 2019;62(5):313–20. | stroke severity not defined |
| Ding L, Wang X, Chen S, Wang H, Tian J, Rong J, u. a. Camera-based mirror visual input for priming promotes motor recovery, daily function, and brain network segregation in subacute stroke patients. Neurorehabilitation and Neural Repair. Januar 2019;33(4):307–18. | Not severe |
| Dolganov MV, Karpova MI. [Virtual reality in upper extremity dysfunction: specific features of usage in acute stroke]. Voprosy kurortologii, fizioterapii, i lechebnoi fizicheskoi kultury. Januar 2019;96(5):19–28. | Wrong language |
| Dong XL, Sun X, Sun WM, Yuan Q, Yu GH, Shuai L, u. a. A randomized controlled trial to explore the efficacy and safety of transcranial direct current stimulation on patients with post-stroke fatigue. Medicine (Baltimore). 2021;100(41):e27504. | Not severe |
| Douglass-Kirk P, Grierson M, Ward NS, Brander F, Kelly K, Chegwidden W, u. a. Real-time auditory feedback may reduce abnormal movements in patients with chronic stroke. Disabil Rehabil. 2023;45(4):613–9. | Wrong setting |
| Dromerick AW, Geed S, Barth J, Brady K, Giannetti ML, Mitchell A, u. a. Critical Period After Stroke Study (CPASS): A phase II clinical trial testing an optimal time for motor recovery after stroke in humans. Proc Natl Acad Sci U S A [Internet]. 2021 [zitiert 1. Januar 9 n. Chr.];118(39). Verfügbar unter: https://pubmed.ncbi.nlm.nih.gov/34544853/ | Not severe |
| Du J, Wang S, Cheng Y, Xu J, Li X, Gan Y, u. a. Effects of Neuromuscular Electrical Stimulation Combined with Repetitive Transcranial Magnetic Stimulation on Upper Limb Motor Function Rehabilitation in Stroke Patients with Hemiplegia. Comput Math Methods Med. 2022;2022:9455428. | stroke severity not defined |
| Dur, Matthew J., Boerger TF, Nguyen JN, Alqahtani SZ, Wright MT, u. a. Two weeks of ischemic conditioning improves walking speed and reduces neuromuscular fatigability in chronic stroke survivors. Journal of applied physiology (Bethesda, Md : 1985). März 2019;126(3):755–63. | stroke severity not defined |
| Edwards D, Cortes M, Rykman-Peltz A, Chang J, Elder J, Thickbroom G, u. a. Clinical improvement with intensive robot-assisted arm training in chronic stroke is unchanged by supplementary tDCS. Restorative neurology and neuroscience. Januar 2019;37(2):167‐180. | stroke severity not defined |
| Ehrensberger M, Simpson D, Broderick P, Blake C, Horgan F, Hickey P, u. a. Unilateral Strength Training and Mirror Therapy in Patients With chronic Stroke: A Pilot Randomized Trial. American Journal of Physical Medicine & Rehabilitation. August 2019;98(8):657–65. | Wrong setting |
| El-Nashar H, Elwishy A, Helmy H, El-Rwainy R. Do core stability exercises improve upper limb function in chronic stroke patients? Egyptian Journal of Neurology, Psychiatry and Neurosurgery. Januar 2019;55(1):1–9. | stroke severity not defined |
| Epple C, Maurer-Burkhard B, Lichti MC, Steiner T. Vojta therapy improves postural control in very early stroke rehabilitation: a randomised controlled pilot trial. Neurological Research and Practice. Dezember 2020;2(1):23. | Not severe |
| Esquenazi A, Brashear A, Deltombe T, Rudzinska-Bar M, Krawczyk M, Skoromets A, u. a. The Effect of Repeated abobotulinumtoxinA (Dysport®) Injections on Walking Velocity in Persons with Spastic Hemiparesis Caused by Stroke or Traumatic Brain Injury. PM R. 2021;13(5):488–95. | stroke severity not defined |
| Ersoy C, Iyigun G. Boxing training in patients with stroke causes improvement of upper extremity, balance, and cognitive functions but should it be applied as virtual or real? Topics in Stroke Rehabilitation. Januar 2021;28(2):112–26. | Not severe |
| Fan Z, Li B, Liao L, Chen Y, Gao Q. Effects of Trunk Control Training on Dynamic Sitting Balance and Trunk Function in Hemiplegia Patients after Acute Stroke. Sichuan da xue xue bao Yi xue ban [Journal of Sichuan University Medical science edition]. Januar 2020;51(6):847‐852. | Wrong language |
| Fishbein P, Hutzler Y, Ratmansky M, Treger I, Dunsky A. A Preliminary Study of Dual-Task Training Using Virtual Reality: Influence on Walking and Balance in Chronic Poststroke Survivors. Journal of Stroke & Cerebrovascular Diseases. November 2019;28(11):N.PAG-N.PAG. | stroke severity not defined |
| Fletcher-Smith JC, Walker DM, Allatt K, Sprigg N, James M, Ratib S, u. a. The ESCAPS study: a feasibility randomized controlled trial of early electrical stimulation to the wrist extensors and flexors to prevent post-stroke complications of pain and contractures in the paretic arm. Clinical Rehabilitation. Dezember 2019;33(12):1919–30. | Not severe |
| Fong KN, Yang NY, Chan MK, Chan DY, Lau AF, Chan DY, u. a. Combined effects of sensory cueing and limb activation on unilateral neglect in subacute left hemiplegic stroke patients: a randomized controlled pilot study. Clin Rehabil. Juli 2013;27(7):628–37. | No severity measure |
| Foong R, Tang N, Chew E, Chua KSG, Ang KK, Quek C, u. a. Assessment of the Efficacy of EEG-Based MI-BCI with Visual Feedback and EEG Correlates of Mental Fatigue for Upper-Limb Stroke Rehabilitation. IEEE Transactions on Biomedical Engineering. Januar 2020;67(3):786–95. | stroke severity not defined |
| Fu L, Wang F, Ma Z, Zhang J, Xiong W, Wang L. Effect of Acupuncture and Rehabilitation Therapy on the Recovery of Neurological Function and Prognosis of Stroke Patients. Comput Math Methods Med. 2022;2022:4581248. | Wrong intervention |
| Fuentes MA, Borrego A, Noe E, Llorens R. Clinical effectiveness of transcranial direct current stimulation and virtual reality on chronic individuals post-stroke with severe hemiparesis. European Journal of Neurology. Januar 2019;26:182. | Wrong publication date |
| Fuentes MA, Borrego A, Alcanz M, Ciscar R, Lopez R, Colomer C, u. a. Combined effects of transcranial direct current stimulation and virtual reality promotes clinically meaningful improvements on severely impaired upper limb function in individuals with chronic stroke. Brain Injury. Januar 2019;33:177. | Wrong publication date |
| Fujita K, Kobayashi Y, Miaki H, Hori H, Tsushima Y, Sakai R, u. a. Pedaling improves gait ability of hemiparetic patients with stiff-knee gait: fall prevention during gait. Journal of Stroke & Cerebrovascular Diseases. September 2020;29(9):N.PAG-N.PAG. | stroke severity not defined |
| Gambassi BB, Coelho-Junior HJ, Paixão Dos Santos C, de Oliveira Gonçalves I, Mostarda CT, Marzetti E, u. a. Dynamic Resistance Training Improves Cardiac Autonomic Modulation and Oxidative Stress Parameters in Chronic Stroke Survivors: A Randomized Controlled Trial. Oxidative medicine and cellular longevity. Januar 2019;2019:5382843. | stroke severity not defined |
| Gamez AB, Hern, ez Morante JJ, Martinez Gil JL, Esparza F, Martinez CM. The effect of surface electromyography biofeedback on the activity of extensor and dorsiflexor muscles in elderly adults: a randomized trial. Scientific reports. Januar 2019;9(1):13153. | Not severe |
| Geiger M, Roche N, Vlachos E, Cattagni T, Zory R. Acute effects of bi-hemispheric transcranial direct current stimulation on the neuromuscular function of patients with chronic stroke: a randomized controlled study. Clinical biomechanics (Bristol, Avon). Januar 2019;70:1‐7. | Wrong setting |
| Glize B, Cook A, Benard A, Sagnier S, Olindo S, Poli M, u. a. Early multidisciplinary prevention program of post-stroke shoulder pain: A randomized clinical trial. Clin Rehabil. 2022;36(8):1042–51. | Not severe |
| Gomes CLA, Cacho RO, Nobrega VTB, Galvão F, de Araújo DS, Medeiros AL de S, u. a. Effects of attentional focus on upper extremity motor performance in post stroke patients: A randomized pilot study. Medicine. März 2021;100(9):e24656. | stroke severity not defined |
| Gong Y, Long XM, Xu Y, Cai XY, Ye M. Effects of repetitive transcranial magnetic stimulation combined with transcranial direct current stimulation on motor function and cortex excitability in subacute stroke patients: A randomized controlled trial. Clin Rehabil. 2021;35(5):718–27. | Wrong intervention |
| Gonzalez-Hoelling S, Bertran-Noguer C, Reig-Garcia G, Suñer-Soler R. Effects of a music-based rhythmic auditory stimulation on gait and balance in subacute stroke. International Journal of Environmental Research and Public Health. Januar 2021;18(4):1–14. | Not severe |
| Gueye T, Dedkova M, Rogalewicz V, Grunerova-Lippertova M, Angerova Y. Early post-stroke rehabilitation for upper limb motor function using virtual reality and exoskeleton: equally efficient in older patients. Neurologia i neurochirurgia polska. Januar 2021;55(1):91–6. | No fulltext available |
| Guler MA, Erhan B, Yilmaz Yalcinkaya E. Caregiver burden in stroke inpatients: a randomized study comparing robot-assisted gait training and conventional therapy. Acta Neurol Belg. 2021;121(3):729–36. | stroke severity not defined |
| Guillouet E, Cogne M, Saverot E, Roche N, Pradat-Diehl P, Weill-Chounlamountry A, u. a. Impact of Combined Transcranial Direct Current Stimulation and Speech-language Therapy on Spontaneous Speech in Aphasia: A Randomized Controlled Double-blind Study. Journal of the International Neuropsychological Society : JINS. Januar 2020;26(1):7–18. | stroke severity not defined |
| Hagglund P, Hagg M, Levring Jaghagen E, Larsson B, Wester P. Oral neuromuscular training in patients with dysphagia after stroke: a prospective, randomized, open-label study with blinded evaluators. BMC neurology. Januar 2020;20(1):405. | stroke severity not defined |
| Handlery R, Regan EW, Stewart JC, Pellegrini C, Monroe C, Hainline G, u. a. Predictors of Daily Steps at 1-Year Poststroke: A Secondary Analysis of a Randomized Controlled Trial. Stroke. 2021;52(5):1768–77. | Wrong intervention |
| Hashemi Y, Taghizadeh G, Azad A, Behzadipour S. The effects of supervised and non-supervised upper limb virtual reality exercises on upper limb sensory-motor functions in patients with idiopathic Parkinson’s disease. Hum Mov Sci. 2022;85:102977. | Wrong population |
| He J, Li C, Lin J, Shu B, Ye B, Wang J, u. a. Proprioceptive Training with Visual Feedback Improves Upper Limb Function in Stroke Patients: A Pilot Study. Neural Plast. 2022;2022:1588090. | stroke severity not defined |
| Henrique PPB, Colussi EL, De Marchi ACB. Effects of Exergame on Patients’ Balance and Upper Limb Motor Function after Stroke: A Randomized Controlled Trial. Journal of Stroke & Cerebrovascular Diseases. August 2019;28(8):2351–7. | stroke severity not defined |
| Hernández-Franco J, Orihuela-Espina F, Palafox L, Palencia C, Camberos-Angulo C, Quijada-Cruz MDLR, u. a. Remote central effects of botulinum toxin type A as adjuvant to intense occupational therapy in the early stage of stroke: A Type II fMRI randomised controlled trial. Toxicon. Januar 2021;190:S33. | Wrong intervention |
| Högg S, Holzgraefe M, Drüge C, Hauschild F, Herrmann C, Obermann M, u. a. High-intensity arm resistance training does not lead to better outcomes than low-intensity resistance training in patients after subacute stroke: A randomized controlled trial. Journal of rehabilitation medicine. Juni 2020;52(6):jrm00067. | Not severe |
| Hokazono A, Etoh S, Jonoshita Y, Kawahira K, Shimodozono M. Combination therapy with repetitive facilitative exercise program and botulinum toxin type A to improve motor function for the upper-limb spastic paresis in chronic stroke: A randomized controlled trial. Journal of Hand Therapy [Internet]. Januar 2021; Verfügbar unter: https://www.embase.com/search/results?subaction=viewrecord&id=L2011597003&from=export | Wrong intervention |
| Horsley S, Lannin NA, Hayward KS, Herbert RD. Additional early active repetitive motor training did not prevent contracture in adults receiving task-specific upper limb training after stroke: a randomised trial. Journal of Physiotherapy (Elsevier). April 2019;65(2):88–94. | Not severe |
| Hsieh HC. Use of a Gaming Platform for Balance Training After a Stroke: A Randomized Trial. Archives of Physical Medicine & Rehabilitation. April 2019;100(4):591–7. | stroke severity not defined |
| Hsieh YW, Lin YH, Zhu JD, Wu CY, Lin YP, Chen CC. Treatment Effects of Upper Limb Action Observation Therapy and Mirror Therapy on Rehabilitation Outcomes after Subacute Stroke: A Pilot Study. Behavioural Neurology [Internet]. Januar 2020;2020. Verfügbar unter: https://www.embase.com/search/results?subaction=viewrecord&id=L2004628603&from=export | Not severe |
| Hsu CC, Fu TC, Huang SC, Chen CP, Wang JS. Increased serum brain-derived neurotrophic factor with high-intensity interval training in stroke patients: A randomized controlled trial. Ann Phys Rehabil Med. 2021;64(4):101385. | Not severe |
| Hung JW, Chen YW, Chen YJ, Pong YP, Wu WC, Chang KC, u. a. The Effects of Distributed vs. Condensed Schedule for Robot-Assisted Training with Botulinum Toxin A Injection for Spastic Upper Limbs in Chronic Post-Stroke Subjects. Toxins (Basel) [Internet]. 2021 [zitiert 1. Januar 8 n. Chr.];13(8). Verfügbar unter: https://pubmed.ncbi.nlm.nih.gov/34437410/ | stroke severity not defined |
| Huang M, Miller T, Ying M, Pang M. Whole-body vibration modulates leg muscle reflex and blood perfusion among people with chronic stroke: a randomized controlled crossover trial. Scientific reports. Januar 2020;10(1):1473. | Wrong setting |
| Hung NT, Paul V, Prakash P, Kovach T, Tacy G, Tomic G, u. a. Wearable myoelectric interface enables high-dose, home-based training in severely impaired chronic stroke survivors. Ann Clin Transl Neurol. 2021;8(9):1895–905. | Wrong setting |
| Huang S, Liu P, Chen Y, Gao B, Li Y, Chen C, u. a. Effectiveness of Contralaterally Controlled Functional Electrical Stimulation versus Neuromuscular Electrical Stimulation on Upper Limb Motor Functional Recovery in Subacute Stroke Patients: A Randomized Controlled Trial. Neural Plast. 2021;2021:1987662. | Not severe |
| Huang Y, Nam C, Li W, Rong W, Xie Y, Liu Y, u. a. A comparison of the rehabilitation effectiveness of neuromuscular electrical stimulation robotic hand training and pure robotic hand training after stroke: A randomized controlled trial. Biomedical Signal Processing and Control [Internet]. Januar 2020;56. Verfügbar unter: https://www.embase.com/search/results?subaction=viewrecord&id=L2003556853&from=export | Wrong setting |
| Huang Y, Chen P, Tso H, Yang Y, Ho T, Leong C. Effects of kinesio taping on hemiplegic hand in patients with upper limb post-stroke spasticity: a randomized controlled pilot study. European journal of physical and rehabilitation medicine. Januar 2019;55(5):551‐557. | Not severe |
| Hung C shan, Hsieh Y wei, Wu C yi, Lin K chung, Lin J chi, Yeh L min, u. a. Comparative Assessment of Two Robot-Assisted Therapies for the Upper Extremity in PeopleWith Chronic Stroke. American Journal of Occupational Therapy. Februar 2019;73(1):1–9. | stroke severity not defined |
| Hwang N, Kim H, Shim J, Park J. Tongue stretching exercises improve tongue motility and oromotor function in patients with dysphagia after stroke: a preliminary randomized controlled trial. Archives of oral biology. Januar 2019;108:104521. | stroke severity not defined |
| In Jae Park, Ji-Ho Park, Hyun Yong Seong, Hyun You J (Sung), So Jung Kim, Ji Hong Min, u. a. Comparative Effects of Different Assistance Force During Robot-Assisted Gait Training on Locomotor Functions in Patients With Subacute Stroke: An Assessor-Blind, Randomized Controlled Trial. American Journal of Physical Medicine & Rehabilitation. Januar 2019;98(1):58–64. | stroke severity not defined |
| Iwamoto Y, Imura T, Suzukawa T, Fukuyama H, Ishii T, Taki S, u. a. Combination of Exoskeletal Upper Limb Robot and Occupational Therapy Improve Activities of Daily Living Function in Acute Stroke Patients. Journal of Stroke & Cerebrovascular Diseases. Juli 2019;28(7):2018–25. | Not severe |
| Jan S, Arsh A, Darain H, Gul S. A randomized control trial comparing the effects of motor relearning programme and mirror therapy for improving upper limb motor functions in stroke patients. JPMA The Journal of the Pakistan Medical Association. Januar 2019;69(9):1242–5. | stroke severity not defined |
| Jayaraman A, O’Brien MK, Madhavan S, Mummidisetty CK, Roth HR, Hohl K, u. a. Stride management assist exoskeleton vs functional gait training in stroke: A randomized trial. Neurology. Januar 2019;92(3):132–132. | stroke severity not defined, Wrong setting |
| Je Shik Nam, Tae Im Yi, Hyun Im Moon. Effects of adjuvant mental practice using inverse video of the unaffected upper limb in subacute stroke: a pilot randomized controlled study. International Journal of Rehabilitation Research. Dezember 2019;42(4):337–43. | Not severe |
| Jiang YF, Zhang D, Zhang J, Hai H, Zhao YY, Ma YW. A Randomized Controlled Trial of Repetitive Peripheral Magnetic Stimulation applied in Early Subacute Stroke: Effects on Severe Upper-limb Impairment. Clin Rehabil. 2022;36(5):693–702. | Wrong intervention |
| Jin M, Zhang Z, Bai Z, Fong KNK. Timing-dependent interaction effects of tDCS with mirror therapy on upper extremity motor recovery in patients with chronic stroke: A randomized controlled pilot study. Journal of the Neurological Sciences [Internet]. Januar 2019;405. Verfügbar unter: https://www.embase.com/search/results?subaction=viewrecord&id=L2002776626&from=export | Wrong setting |
| Jongbloed L, Stacey S, Brighton C. Stroke Rehabilitation: Sensorimotor Integrative Treatment Versus Functional Treatment. The American Journal of Occupational Therapy. 1. Juni 1989;43(6):391–7. | Not severe |
| Junata M, Cheng KC, Man HS, Lai CW, Soo YO, Tong RK. Kinect-based rapid movement training to improve balance recovery for stroke fall prevention: a randomized controlled trial. J Neuroeng Rehabil. 2021;18(1):150. | Not severe |
| Jung KM, Choi JD. The Effects of Active Shoulder Exercise with a Sling Suspension System on Shoulder Subluxation, Proprioception, and Upper Extremity Function in Patients with Acute Stroke. Medical science monitor : international medical journal of experimental and clinical research. Januar 2019;25:4849–55. | stroke severity not defined |
| Kang CJ, Chun MH, Lee J, Lee JY. Effects of robot (SUBAR)-assisted gait training in patients with chronic stroke: Randomized controlled trial. Medicine (Baltimore). 2021;100(48):e27974. | Not severe |
| Kang JH, Kim MW, Park KH, Choi YA. The effects of additional electrical stimulation combined with repetitive transcranial magnetic stimulation and motor imagery on upper extremity motor recovery in the subacute period after stroke: A preliminary study. Medicine (Baltimore). 2021;100(35):e27170. | Wrong intervention |
| Kang YS, Oh GB, Cho KH. Walking Training with a Weight Support Feedback Cane Improves Lower Limb Muscle Activity and Gait Ability in Patients with Chronic Stroke: A Randomized Controlled Trial. Med Sci Monit. 2021;27:e931565. | stroke severity not defined |
| Kattenstroth JC, Kalisch T, Sczesny-Kaiser M, Greulich W, Tegenthoff M, Dinse HR. Daily repetitive sensory stimulation of the paretic hand for the treatment of sensorimotor deficits in patients with subacute stroke: RESET, a randomized, sham-controlled trial. BMC Neurology. Dezember 2018;18(1):2. | stroke severity not defined |
| Kerimov K, Coskun Benlidayi I, Ozdemir C, Gunasti O. The Effects of Upper Extremity Isokinetic Strengthening in Post-Stroke Hemiplegia: A Randomized Controlled Trial. J Stroke Cerebrovasc Dis. 2021;30(6):105729. | stroke severity not defined |
| Kerr A, Clark A, Pomeroy V. Neuromechanical Differences Between Successful and Failed Sit-to-Stand Movements and Response to Rehabilitation Early After Stroke. Neurorehabilitation and neural repair. Januar 2019;33(5):395‐403. | stroke severity not defined |
| Khallaf ME. Effect of Task-Specific Training on Trunk Control and Balance in Patients with Subacute Stroke. NEUROLOGY RESEARCH INTERNATIONAL. November 2020;2020. | Wrong setting |
| Kim BR, Kang TW. The effects of proprioceptive neuromuscular facilitation lower-leg taping and treadmill training on mobility in patients with stroke. International Journal of Rehabilitation Research. Dezember 2018;41(4):343–8. | stroke severity not defined |
| Kim HY, Shin JH, Yang SP, Shin MA, Lee SH. Robot-assisted gait training for balance and lower extremity function in patients with infratentorial stroke: A single-blinded randomized controlled trial. Journal of NeuroEngineering and Rehabilitation [Internet]. Januar 2019;16(1). Verfügbar unter: https://www.embase.com/search/results?subaction=viewrecord&id=L628714759&from=export | stroke severity not defined |
| Kim H, Park G, Shin JH, You JH. Neuroplastic effects of end-effector robotic gait training for hemiparetic stroke: a randomised controlled trial. Scientific reports. Januar 2020;10(1):12461. | stroke severity not defined |
| Kim H, Park J. Efficacy of modified chin tuck against resistance exercise using hand-free device for dysphagia in stroke survivors: a randomised controlled trial. Journal of oral rehabilitation. Januar 2019;46(11):1042‐1046. | stroke severity not defined |
| Kim J, Lee M, Yim J. A New Approach to Transcranial Direct Current Stimulation in Improving Cognitive Motor Learning and Hand Function with the Nintendo Switch in Stroke Survivors. Medical science monitor. Januar 2019;25:9555‐9562. | stroke severity not defined |
| Kim J, Kim DY, Chun MH, Kim SW, Jeon HR, Hwang CH, u. a. Effects of robot-(Morning Walk(®)) assisted gait training for patients after stroke: a randomized controlled trial. Clinical rehabilitation. März 2019;33(3):516–23. | Not severe |
| Kim MS, Kim SH, Noh SE, Bang HJ, Lee KM. Robotic-Assisted Shoulder Rehabilitation Therapy Effectively Improved Poststroke Hemiplegic Shoulder Pain: A Randomized Controlled Trial. Archives of Physical Medicine & Rehabilitation. Juni 2019;100(6):1015–22. | No subanalysis  Not severe |
| Kim SH, Park JH. The Effect of Occupation-Based Bilateral Upper Extremity Training in a Medical Setting for Stroke Patients: A Single-Blinded, Pilot Randomized Controlled Trial. Journal of Stroke & Cerebrovascular Diseases. Dezember 2019;28(12):N.PAG-N.PAG. |  |
| Kim SH. Effects of Dual Transcranial Direct Current Stimulation and Modified Constraint-Induced Movement Therapy to Improve Upper-Limb Function after Stroke: A Double-Blinded, Pilot Randomized Controlled Trial. J Stroke Cerebrovasc Dis. 2021;30(9):105928. | stroke severity not defined |
| Kirac-Unal Z, Gencay-Can A, Karaca-Umay E, Cakci FA. The effect of task-oriented electromyography-triggered electrical stimulation of the paretic wrist extensors on upper limb motor function early after stroke: a pilot randomized controlled trial. International Journal of Rehabilitation Research. März 2019;42(1):74–81. | stroke severity not defined |
| Kitatani R, Koganemaru S, Maeda A, Mikami Y, Matsuhashi M, Mima T, u. a. Gait-synchronized oscillatory brain stimulation modulates common neural drives to ankle muscles in patients after stroke: A pilot study. Neuroscience Research. Januar 2020;156:256–64. | Not severe |
| Klassen TD, Dukelow SP, Bayley MT, Benavente O, Hill MD, Krassioukov A, u. a. Determining optimal poststroke exercise: Study protocol for a randomized controlled trial investigating therapeutic intensity and dose on functional recovery during stroke inpatient rehabilitation. International Journal of Stroke. Januar 2019;14(1):80–6. | Not severe |
| Klassen TD, Dukelow SP, Bayley MT, Benavente O, Hill MD, Krassioukov A, u. a. Higher Doses Improve Walking Recovery during Stroke Inpatient Rehabilitation. Stroke. Januar 2020;2639–48. | Wrong study design, protocol |
| Koch S, Tiozzo E, Simonetto M, Loewenstein D, Wright CB, Dong C, u. a. Randomized Trial of Combined Aerobic, Resistance, and Cognitive Training to Improve Recovery From Stroke: Feasibility and Safety. Journal of the American Heart Association. Januar 2020;9(10):e015377. | Not severe |
| Kotov S, Isakova E, Lijdvoy V, Petrushanskaya K, Pismennaya E, Romanova M, u. a. Robotic recovery of walking function in patients in the early recovery period of stroke. Zhurnal nevrologii i psikhiatrii imeni SS Korsakova. Januar 2020;120(8):73‐80. | Wrong language |
| Kotov S, Isakova E, Sheregeshev V. Possibility of treatment of emotional and behavioral disorders in patients with stroke during rehabilitation. Zhurnal nevrologii i psikhiatrii imeni SS Korsakova. Januar 2019;119(4):26‐31. | Wrong language |
| Krakauer JW, Kitago T, Goldsmith J, Ahmad O, Roy P, Stein J, u. a. Comparing a Novel Neuroanimation Experience to Conventional Therapy for High-Dose Intensive Upper-Limb Training in Subacute Stroke: The SMARTS2 Randomized Trial. Neurorehabil Neural Repair. 2021;35(5):393–405. | Not severe |
| Kuzu Ö, Adiguzel E, Kesikburun S, Yaşar E, Yılmaz B. The Effect of Sham Controlled Continuous Theta Burst Stimulation and Low Frequency | Wrong intervention |
| Laffont I, Froger J, Jourdan C, Bakhti K, van Dokkum LEH, Gouaich A, u. a. Rehabilitation of the upper arm early after stroke: Video games versus conventional rehabilitation. A randomized controlled trial. Annals of Physical and Rehabilitation Medicine. Januar 2020;63(3):173–80. | stroke severity not defined |
| Lapointe T, Trudeau F, Sia YT, Houle J. Postexercise hypotensive response in stroke patients following acute moderate or high intensity cycling session. J Sports Med Phys Fitness. 2022;62(7):974–80. | stroke severity not defined |
| Lee D, Lee G. Effect of afferent electrical stimulation with mirror therapy on motor function, balance, and gait in chronic stroke survivors: a randomized controlled trial. European journal of physical and rehabilitation medicine. Januar 2019;55(4):442–9. | stroke severity not defined |
| Lee G. Whole-Body Vibration in Horizontal Direction for Stroke Rehabilitation: A Randomized Controlled Trial. Medical science monitor : international medical journal of experimental and clinical research. März 2019;25:1621–8. | stroke severity not defined |
| Lee HJ, Lee SH, Seo K, Lee M, Chang WH, Choi BO, u. a. Training for walking efficiency with a wearable hip-assist robot in patients with stroke a pilot randomized controlled trial. Stroke. Januar 2019;50(12):3545–52. | stroke severity not defined |
| Lee J, Jeon J, Lee D, Hong J, Yu J, Kim J. Effect of trunk stabilization exercise on abdominal muscle thickness, balance and gait abilities of patients with hemiplegic stroke: A randomized controlled trial. NeuroRehabilitation. Januar 2020;47(4):435–42. | stroke severity not defined |
| Lee K. Speed-interactive pedaling training using smartphone virtual reality application for stroke patients: Single-blinded, randomized clinical trial. Brain Sciences [Internet]. Januar 2019;9(11). Verfügbar unter: https://www.embase.com/search/results?subaction=viewrecord&id=L2002891728&from=export | stroke severity not defined |
| Lee K, Park D, Lee G. Progressive Respiratory Muscle Training for Improving Trunk Stability in Chronic Stroke Survivors: A Pilot Randomized Controlled Trial. Journal of stroke and cerebrovascular diseases : the official journal of National Stroke Association. Januar 2019;28(5):1200–11. | Not severe |
| Lee P, Huang J, Tseng H, Yang Y, Lin S. Effects of Trunk Exercise on Unstable Surfaces in Persons with Stroke: a Randomized Controlled Trial. International journal of environmental research and public health [Internet]. Januar 2020;17(23). Verfügbar unter: https://www.cochranelibrary.com/central/doi/10.1002/central/CN-02210793/full | stroke severity not defined |
| Lee SH, Park G, Cho DY, Kim HY, Lee J-Y, Kim S, u. a. Comparisons between end-effector and exoskeleton rehabilitation robots regarding upper extremity function among chronic stroke patients with moderate-to-severe upper limb impairment. Scientific Reports. Januar 2020;10:1806. | stroke severity not defined |
| Lerma Castaño PR, Rodríguez Laiseca YA, Montealegre Suárez DP, Castrillón Papamija DB, Losada Urriago GE. Effects of kinesiotaping combined with the motor relearning method on upper limb motor function in adults with hemiparesis after stroke. Journal of Bodywork & Movement Therapies. Oktober 2020;24(4):546–53. | stroke severity not defined |
| Levin MF, Hiengkaew V, Nilanont Y, Cheung D, Dai D, Shaw J, u. a. Relationship Between Clinical Measures of Upper Limb Movement Quality and Activity Poststroke. Neurorehabilitation and neural repair. Januar 2019;33(6):432–41. | Wrong design |
| Li DX, Zha FB, Long JJ, Liu F, Cao J, Wang YL. Effect of Robot Assisted Gait Training on Motor and Walking Function in Patients with Subacute Stroke: A Random Controlled Study. J Stroke Cerebrovasc Dis. 2021;30(7):105807. | Not severe |
| Liang CC, Hsieh TC, Lin CH, Wei YC, Hsiao J, Chen JC. Effectiveness of Thermal Stimulation for the Moderately to Severely Paretic Leg After Stroke: Serial Changes at One-Year Follow-Up. Archives of Physical Medicine and Rehabilitation. November 2012;93(11):1903–10. | No severity measure |
| Liao WW, Chiang WC, Lin KC, Wu CY, Liu CT, Hsieh YW, u. a. Timing-dependent effects of transcranial direct current stimulation with mirror therapy on daily function and motor control in chronic stroke: a randomized controlled pilot study. Journal of neuroengineering and rehabilitation. Januar 2020;17(1):101. | Not severe |
| Liaw MY, Hsu CH, Leong CP, Liao CY, Wang LY, Lu CH, u. a. Respiratory muscle training in stroke patients with respiratory muscle weakness, dysphagia, and dysarthria - a prospective randomized trial. Medicine. Januar 2020;99(10):e19337. | Not severe |
| Lin JH, Lin SF, Wei YY. Effect of transcranial direct current stimulation combined with neuromuscular electrical stimulation on motor recovery of upper extremity in patients with chronic stroke. Neuromodulation. Januar 2019;22(7):e372. | Wrong publication type |
| Lin R, Chiang S, Heitkemper MM, Weng S, Lin C, Yang F, u. a. Effectiveness of Early Rehabilitation Combined With Virtual Reality Training on Muscle Strength, Mood State, and Functional Status in Patients With Acute Stroke: A Randomized Controlled Trial. Worldviews on Evidence-Based Nursing. April 2020;17(2):158–67. | Not severe |
| Linder SM, Rosenfeldt AB, Davidson S, Zimmerman N, Penko A, Lee J, u. a. Forced, Not Voluntary, Aerobic Exercise Enhances Motor Recovery in Persons With Chronic Stroke. Neurorehabilitation and Neural Repair. Januar 2019;33(8):681–90. | stroke severity not defined |
| Liu M, Xu L, Li H, Chen S, Chen B. Morphological and Functional Changes of the Tibialis Anterior Muscle After Combined Mirror Visual Feedback and Electromyographic Biofeedback in Poststroke Patients: A Randomized Trial. Am J Phys Med Rehabil. 2021;100(8):766–73. | stroke severity not defined |
| Luk KY, Ouyang HX, Pang MYC. Low-Frequency rTMS over Contralesional M1 Increases Ipsilesional Cortical Excitability and Motor Function with Decreased Interhemispheric Asymmetry in Subacute Stroke: A Randomized Controlled Study. Neural Plast. 2022;2022:3815357. | Wrong intervention |
| Lura D, Venglar M, van Duijn A, Csavina K. Body weight supported treadmill vs. overground gait training for acute stroke gait rehabilitation. International journal of rehabilitation research Internationale Zeitschrift fur Rehabilitationsforschung Revue internationale de recherches de readaptation. Januar 2019;42(3):270‐274. | stroke severity not defined |
| Madhoun HY, Tan B, Feng Y, Zhou Y, Zhou C, Yu L. Task-based mirror therapy enhances the upper limb motor function in subacute stroke patients: a randomized control trial. European Journal of Physical and Rehabilitation Medicine. Januar 2020;56(3):265–71. | Not severe |
| Madhuranga PVH, Mathangasinghe Y, Anthony DJ. Improving balance with wobble board exercises in stroke patients: single-blind, randomized clinical trial. Topics in stroke rehabilitation. Januar 2019;26(8):595–601. | stroke severity not defined |
| Mandigout S, Chaparro D, Borel B, Kammoun B, Salle JY, Compagnat M, u. a. Effect of individualized coaching at home on walking capacity in subacute stroke patients: A randomized controlled trial (Ticaa’dom). Ann Phys Rehabil Med. 2021;64(4):101453. | Not severe |
| Manuli A, Maggio MG, Latella D, Cannavò A, Balletta T, De Luca R, u. a. Can robotic gait rehabilitation plus Virtual Reality affect cognitive and behavioural outcomes in patients with chronic stroke? A randomized controlled trial involving three different protocols. Journal of Stroke & Cerebrovascular Diseases. August 2020;29(8):N.PAG-N.PAG. | Not severe |
| Mao H, Li Y, Tang L, Chen Y, Ni J, Liu L, u. a. Effects of mirror neuron system-based training on rehabilitation of stroke patients. Brain and Behavior [Internet]. Januar 2020;10(8). Verfügbar unter: https://www.embase.com/search/results?subaction=viewrecord&id=L2005420067&from=export | Not severe |
| Mao YR, Zhao JL, Bian MJ, Lo WLA, Leng Y, Bian RH, u. a. Spatiotemporal, kinematic and kinetic assessment of the effects of a foot drop stimulator for home-based rehabilitation of patients with chronic stroke: a randomized clinical trial. J Neuroeng Rehabil. 2022;19(1):56. | Not severe |
| Marshall J, Caute A, Chadd K, Cruice M, Monnelly K, Wilson S, u. a. Technology-enhanced writing therapy for people with aphasia: results of a quasi-randomized waitlist controlled study. International journal of language & communication disorders. Januar 2019;54(2):203‐220. | Wrong setting, stroke severity not defined |
| Martins JC, Nadeau S, Aguiar LT, Scianni AA, Teixeira-Salmela LF, De Morais Faria CDC. Efficacy of task-specific circuit training on physical activity levels and mobility of stroke patients: A randomized controlled trial. NeuroRehabilitation. Januar 2020;47(4):451–62. | Not severe |
| Marupuru S, Bell ML, Grandner MA, Taylor-Piliae RE. The Effect of Physical Activity on Sleep Quality among Older Stroke Survivors: Secondary Analysis from a Randomized Controlled Trial. Int J Environ Res Public Health [Internet]. 2022 [zitiert 1. Januar 10 n. Chr.];19(20). Verfügbar unter: https://pubmed.ncbi.nlm.nih.gov/36293902/ | stroke severity not defined |
| Mateen FJ, Massawe E, Mworia NA, Ismail S, Rice DR, Vogel AC, u. a. Measuring Ambulation, Motor, and Behavioral Outcomes with Post-stroke Fluoxetine in Tanzania: The Phase II MAMBO Trial. Am J Trop Med Hyg. 2021;106(3):970–8. | Wrong intervention |
| Matsumoto S, Shimodozono M, Noma T. Rationale and design of the theRapeutic effects of peroneal nerve functionAl electrical stimuLation for Lower extremitY in patients with convalescent poststroke hemiplegia (RALLY) study: Study protocol for a randomised controlled study. BMJ Open [Internet]. Januar 2019;9(11). Verfügbar unter: https://www.embase.com/search/results?subaction=viewrecord&id=L629968924&from=export | stroke severity not defined  wrong study design |
| May HI, Özdolap Åž, Mengi A, Sarikaya S. The effect of mirror therapy on lower extremity motor function and ambulation in post-stroke patients: A prospective, randomized-controlled study. Turkish Journal of Physical Medicine and Rehabilitation. Januar 2020;66(2):154–60. | Not severe |
| Mazzoleni S, Tran VD, Dario P, Posteraro F. Effects of Transcranial Direct Current Stimulation (tDCS) Combined With Wrist Robot-Assisted Rehabilitation on Motor Recovery in Subacute Stroke Patients: A Randomized Controlled Trial. IEEE Transactions on Neural Systems and Rehabilitation Engineering. Januar 2019;27(7):1458–66. | stroke severity not defined |
| Mediano MFF, Mok Y, Coresh J, Kucharska-Newton A, Palta P, Lakshminarayan K, u. a. Prestroke Physical Activity and Adverse Health Outcomes After Stroke in the Atherosclerosis Risk in Communities Study. Stroke. 2021;52(6):2086–95. | Wrong setting, wrong intervention |
| Mekbib DB, Debeli DK, Zhang L, Fang S, Shao Y, Yang W, u. a. A novel fully immersive virtual reality environment for upper extremity rehabilitation in patients with stroke. Ann N Y Acad Sci. 2021;1493(1):75–89. | Not severe |
| Miller A, Pohlig RT, Wright T, Kim HE, Reisman DS. Beyond Physical Capacity: Factors Associated With Real-world Walking Activity After Stroke. Arch Phys Med Rehabil. 2021;102(10):1880-1887.e1. | stroke severity not defined |
| Milot MH, Léonard G, Corriveau H, Desrosiers J. Using the Borg rating of perceived exertion scale to grade the intensity of a functional training program of the affected upper limb after a stroke: a feasibility study. Clinical interventions in aging. Januar 2019;14:9–16. | stroke severity not defined |
| Min M, Xin C, Yuefeng C, Ping R, Jian L. Stage-oriented Comprehensive Acupuncture Treatment plus Rehabilitation Training for Apoplectic Hemiplegia. Journal of Traditional Chinese Medicine. Juni 2008;28(2):90–3. | Wrong intervention |
| Mitsutake T, Sakamoto M, Nakazono H, Horikawa E. The Effects of Combining Transcranial Direct Current Stimulation and Gait Training with Functional Electrical Stimulation on Trunk Acceleration During Walking in Patients with Subacute Stroke. Journal of stroke and cerebrovascular diseases : the official journal of National Stroke Association. April 2021;30(4):105635. | Not severe |
| Mitsutake T, Sakamoto M, Nakazono H, Horikawa E. The Effects of Combining Transcranial Direct Current Stimulation and Gait Training with Functional Electrical Stimulation on Trunk Acceleration During Walking in Patients with Subacute Stroke. J Stroke Cerebrovasc Dis. 2021;30(4):105635. | stroke severity not defined |
| Mizuta N, Hasui N, Nishi Y, Higa Y, Matsunaga A, Deguchi J, u. a. Merged swing-muscle synergies and their relation to walking characteristics in subacute post-stroke patients: An observational study. PLoS One. 2022;17(2):e0263613. | Not severe |
| Mollà-Casanova S, Llorens R, Borrego A, Salinas-Martínez B, Serra-Añó P. Validity, reliability, and sensitivity to motor impairment severity of a multi-touch app designed to assess hand mobility, coordination, and function after stroke. J Neuroeng Rehabil. 2021;18(1):70. | stroke severity not defined |
| Morice E, Moncharmont J, Jenny C, Bruyneel AV. Dancing to improve balance control, cognitive-motor functions and quality of life after stroke: a study protocol for a randomised controlled trial. BMJ open. Januar 2020;10(9):e037039‐. | stroke severity not defined |
| Morone G, Bragoni M, Iosa M, De Angelis D, Venturiero V, Coiro P, u. a. Who May Benefit From Robotic-Assisted Gait Training?: A Randomized Clinical Trial in Patients With Subacute Stroke. Neurorehabil Neural Repair. September 2011;25(7):636–44. | Not severe |
| Morone G, Iosa M, Bragoni M, De Angelis D, Venturiero V, Coiro P, u. a. Who May Have Durable Benefit From Robotic Gait Training?: A 2-Year Follow-Up Randomized Controlled Trial in Patients With Subacute Stroke. Stroke. April 2012;43(4):1140–2. | Not severe |
| Morris J, John A, Wedderburn L, Rauchhaus P, Donnan P. Dynamic Lycra® orthoses as an adjunct to arm rehabilitation after stroke: a single-blind, two-arm parallel group, randomized controlled feasibility trial. Clinical rehabilitation. Januar 2019;33(8):1331‐1343. | Not severe |
| Munari D, Serina A, DIsarò J, Modenese A, Filippetti M, G, u. a. Combined effects of backward treadmill training and botulinum toxin type A therapy on gait and balance in patients with chronic stroke: A pilot, single-blind, randomized controlled trial. NeuroRehabilitation. Januar 2020;46(4):519–28. | Wrong intervention |
| Nam YG, Lee JW, Park JW, Lee HJ, Nam KY, Park JH, u. a. Effects of Electromechanical Exoskeleton-Assisted Gait Training on Walking Ability of Stroke Patients: A Randomized Controlled Trial. Archives of physical medicine and rehabilitation. Januar 2019;100(1):26–31. | stroke severity not defined |
| Nasb M, Li Z, S.A. Youssef A, Dayoub L, Chen H. Comparison of the effects of modified constraint-induced movement therapy and intensive conventional therapy with a botulinum-a toxin injection on upper limb motor function recovery in patients with stroke. Libyan Journal of Medicine [Internet]. Januar 2019;14(1). Verfügbar unter: https://www.embase.com/search/results?subaction=viewrecord&id=L627418693&from=export | Wrong intervention |
| NCT03817086. Physical and Mental Practice for Bimanual Coordination Rehabilitation. https://clinicaltrials.gov/show/NCT03817086 [Internet]. Januar 2019; Verfügbar unter: https://www.cochranelibrary.com/central/doi/10.1002/central/CN-01796072/full | ongoing |
| NCT04011202. Virtual Reality, Mood, and Sedentary Behaviour After Stroke. https://clinicaltrials.gov/show/NCT04011202 [Internet]. Januar 2019; Verfügbar unter: https://www.cochranelibrary.com/central/doi/10.1002/central/CN-01953109/full | ongoing |
| NCT04027985. Functional Outcome of Hemiplegic Upper Extremity in Patients With Subacute Stroke After Kinesiotaping and Rehabilitation. https://clinicaltrials.gov/show/NCT04027985 [Internet]. Januar 2019; Verfügbar unter: https://www.cochranelibrary.com/central/doi/10.1002/central/CN-01965681/full | ongoing |
| NCT04051658. Hemodynamic Response and Motor Functions Following Transcranial Direct Current Stimulation in Acute Stroke. https://clinicaltrials.gov/show/NCT04051658 [Internet]. Januar 2019; Verfügbar unter: https://www.cochranelibrary.com/central/doi/10.1002/central/CN-01966319/full | ongoing |
| NCT04086004. Dual Task Balance Training With Additional Motor Imagery Practice in Stroke. https://clinicaltrials.gov/show/NCT04086004 [Internet]. Januar 2019; Verfügbar unter: https://www.cochranelibrary.com/central/doi/10.1002/central/CN-01984204/full | ongoing |
| NCT04120467. Dance as a Means to Improve Functions and Quality of Life After a Stroke. https://clinicaltrials.gov/show/NCT04120467 [Internet]. Januar 2019; Verfügbar unter: https://www.cochranelibrary.com/central/doi/10.1002/central/CN-01992687/full | ongoing |
| NCT04144556. Nintendo Wii® and Physical Therapy in Stroke Patients (NW-SP). https://clinicaltrials.gov/show/NCT04144556 [Internet]. Januar 2019; Verfügbar unter: https://www.cochranelibrary.com/central/doi/10.1002/central/CN-02001503/full | ongoing |
| NCT04296032. Effects of Virtual Reality Game on Upper Extremity Function for Stroke. https://clinicaltrials.gov/show/NCT04296032 [Internet]. Januar 2020; Verfügbar unter: https://www.cochranelibrary.com/central/doi/10.1002/central/CN-02088776/full | ongoing |
| NCT04306120. Effects of Thermal Stimulation on Motor Recovery and Neuromuscular Property of Lower Extremity in Stroke. https://clinicaltrials.gov/show/NCT04306120 [Internet]. Januar 2020; Verfügbar unter: https://www.cochranelibrary.com/central/doi/10.1002/central/CN-02089013/full | ongoing |
| NCT04323501. Post-stroke Recovery (PSR_e2020). https://clinicaltrials.gov/show/NCT04323501 [Internet]. Januar 2020; Verfügbar unter: https://www.cochranelibrary.com/central/doi/10.1002/central/CN-02091097/full | ongoing |
| NCT04378946. Error Augmentation Motor Learning Training Approach in Stroke Patients. https://clinicaltrials.gov/show/NCT04378946 [Internet]. Januar 2020; Verfügbar unter: https://www.cochranelibrary.com/central/doi/10.1002/central/CN-02103797/full | ongoing |
| NCT04504214. Tendon Vibrations Effect on Upper Limb Motor Recovery After Recent Stroke. https://clinicaltrials.gov/show/NCT04504214 [Internet]. Januar 2020; Verfügbar unter: https://www.cochranelibrary.com/central/doi/10.1002/central/CN-02145919/full | ongoing |
| NCT04557839. Balance Training on Vestibular Function and Proprioception Feedback in Stroke Patient. https://clinicaltrials.gov/show/NCT04557839 [Internet]. Januar 2020; Verfügbar unter: https://www.cochranelibrary.com/central/doi/10.1002/central/CN-02181278/full | ongoing |
| NCT04577287. The Effects of Anodal and Cathodal tDCS Combined With Conventional Physical Therapy in Patients With Acute Stroke. https://clinicaltrials.gov/show/NCT04577287 [Internet]. Januar 2020; Verfügbar unter: https://www.cochranelibrary.com/central/doi/10.1002/central/CN-02181792/full | ongoing |
| NCT04668573. Turning Dysfunction After Stroke: assessment and Intervention. https://clinicaltrials.gov/show/NCT04668573 [Internet]. Januar 2020; Verfügbar unter: https://www.cochranelibrary.com/central/doi/10.1002/central/CN-02200562/full | ongoing |
| NCT04669431. Xbox Kinect Training on Upper Limb Motor Function in Stroke Patients. https://clinicaltrials.gov/show/NCT04669431 [Internet]. Januar 2020; Verfügbar unter: https://www.cochranelibrary.com/central/doi/10.1002/central/CN-02206466/full | ongoing |
| NCT04777253. Rehabilitation of Arm Function Using a Biofeedback Method After Stroke. https://clinicaltrials.gov/show/NCT04777253 [Internet]. Januar 2021; Verfügbar unter: https://www.cochranelibrary.com/central/doi/10.1002/central/CN-02249437/full | ongoing |
| NCT04778475. Impact of More Frequent PT Services. https://clinicaltrials.gov/show/NCT04778475 [Internet]. Januar 2021; Verfügbar unter: https://www.cochranelibrary.com/central/doi/10.1002/central/CN-02249459/full | ongoing |
| Noh HJ, Lee SH, Bang DH. Three-Dimensional Balance Training Using Visual Feedback on Balance and Walking Ability in Subacute Stroke Patients: A Single-Blinded Randomized Controlled Pilot Trial. Journal of Stroke & Cerebrovascular Diseases. April 2019;28(4):994–1000. | stroke severity not defined |
| Ogino T, Kanata Y, Uegaki R, Yamaguchi T, Morisaki K, Nakano S, u. a. Effects of gait exercise assist robot (GEAR) on subjects with chronic stroke: A randomized controlled pilot trial. Journal of Stroke & Cerebrovascular Diseases. August 2020;29(8):N.PAG-N.PAG. | stroke severity not defined |
| Ögün M, Kurul R, Yaşar M, Turkoglu S, Avci Ş, Yildiz N. Effect of Leap Motion-based 3D Immersive Virtual Reality Usage on Upper Extremity Function in Ischemic Stroke Patients. Arquivos de neuro-psiquiatria. Januar 2019;77(10):681‐688. | Stroke severity not defined |
| Oh DH, Park JS, Kim HJ, Chang MY, Hwang NK. The effect of neuromuscular electrical stimulation with different electrode positions on swallowing in stroke patients with oropharyngeal dysphagia: A randomized trial. Journal of back and musculoskeletal rehabilitation. Januar 2020;33(4):637–44. | stroke severity not defined |
| Oh YB, Kim GW, Han KS, Won YH, Park SH, Seo JH, u. a. Efficacy of Virtual Reality Combined With Real Instrument Training for Patients With Stroke: A Randomized Controlled Trial. Archives of Physical Medicine & Rehabilitation. August 2019;100(8):1400–8. | stroke severity not defined |
| Ooi HK, Chai SC, Kadar M. Effects of pressure garment on spasticity and function of the arm in the early stages after stroke: a randomized controlled trial. Clinical rehabilitation. Januar 2020;34(4):515–23. | stroke severity not defined |
| Osteresch R, Fach A, Frielitz FS, Meyer S, Schmucker J, Rühle S, u. a. Long-Term Effects of an Intensive Prevention Program After Acute Myocardial Infarction. Am J Cardiol. 2021;154:7–13. | Wrong population |
| Ozen S, Senlikci HB, Guzel S, Yemisci OU. Computer Game Assisted Task Specific Exercises in the Treatment of Motor and Cognitive Function and Quality of Life in Stroke: A Randomized Control Study. J Stroke Cerebrovasc Dis. 2021;30(9):105991. | stroke severity not defined |
| Pagilla V, Kumar V, Joshua A, Chakrapani M, Misri ZK, Mithra P. A top-down versus bottom-up approach to lower-extremity motor recovery and balance following acute stroke: A pilot randomized clinical trial. Critical Reviews in Physical and Rehabilitation Medicine. Januar 2019;31(2):135–46. | not severe |
| Pain LAM, Baker R, Sohail QZ, Hebert D, Zabjek K, Richardson D, u. a. The three-dimensional shoulder pain alignment (3D-SPA) mobilization improves pain-free shoulder range, functional reach and sleep following stroke: a pilot randomized control trial. Disability & Rehabilitation. Oktober 2020;42(21):3072–83. | stroke severity not defined |
| Pak NW, Lee JH. Effects of visual feedback training and visual targets on muscle activation, balancing, and walking ability in adults after hemiplegic stroke: a preliminary, randomized, controlled study. International Journal of Rehabilitation Research. März 2020;43(1):76–81. | stroke severity not defined |
| Park C, Oh-Park M, Bialek A, Friel K, Edwards D, You JSH. Abnormal synergistic gait mitigation in acute stroke using an innovative ankle-knee-hip interlimb humanoid robot: a preliminary randomized controlled trial. Sci Rep. 2021;11(1):22823. | stroke severity not defined |
| Park D, Lee JH, Kang TW, Cynn H seock. Effects of a 4-Week Self-Ankle Mobilization with Movement Intervention on Ankle Passive Range of Motion, Balance, Gait, and Activities of Daily Living in Patients with Chronic Stroke: A Randomized Controlled Study. Journal of Stroke & Cerebrovascular Diseases. Dezember 2018;27(12):3451–9. | Not severe |
| Park D, Lee JH, Kang TW, Cynn HS. Four-week training involving ankle mobilization with movement versus static muscle stretching in patients with chronic stroke: a randomized controlled trial. Topics in Stroke Rehabilitation. März 2019;26(2):81–6. | stroke severity not defined |
| Park HS, Oh DH, Yoon T, Park JS. Effect of effortful swallowing training on tongue strength and oropharyngeal swallowing function in stroke patients with dysphagia: a double-blind, randomized controlled trial. International journal of language & communication disorders. Januar 2019;54(3):479–84. | stroke severity not defined |
| Park H, Lee H, Lee S, Lee W. Land-based and aquatic trunk exercise program improve trunk control, balance and activities of daily living ability in stroke: a randomized clinical trial. European journal of physical and rehabilitation medicine. Januar 2019;55(6):687‐694. | Not severe |
| Park JS, An DH, Kam KY, Yoon T, Kim T, Chang MY. Effects of resistive jaw opening exercise in stroke patients with dysphagia: A double- blind, randomized controlled study. Journal of back and musculoskeletal rehabilitation. Januar 2020;33(3):507–13. | stroke severity not defined |
| Park JH, Park G, Kim HY, Lee JY, Ham Y, Hwang D, u. a. A comparison of the effects and usability of two exoskeletal robots with and without robotic actuation for upper extremity rehabilitation among patients with stroke: a single-blinded randomised controlled pilot study. Journal of neuroengineering and rehabilitation. Oktober 2020;17(1):137. | stroke severity not defined |
| Park JH. The effects of robot-assisted left-hand training on hemispatial neglect in older patients with chronic stroke: A pilot and randomized controlled trial. Medicine. Januar 2021;100(9):e24781. | stroke severity not defined |
| Park J, Lee G, Jung Y. Effects of game-based chin tuck against resistance exercise vs head-lift exercise in patients with dysphagia after stroke: an assessor-blind, randomized controlled trial. Journal of rehabilitation medicine. Januar 2019;51(10):749‐754. | stroke severity not defined |
| Park M, Ko MH, Oh SW, Lee JY, Ham Y, Yi H, u. a. Effects of virtual reality-based planar motion exercises on upper extremity function, range of motion, and health-related quality of life: A multicenter, single-blinded, randomized, controlled pilot study. Journal of NeuroEngineering and Rehabilitation [Internet]. Januar 2019;16(1). Verfügbar unter: https://www.embase.com/search/results?subaction=viewrecord&id=L629717608&from=export | Not severe |
| Park MO, Lee SH. Effect of a dual-task program with different cognitive tasks applied to stroke patients: A pilot randomized controlled trial. NeuroRehabilitation. Januar 2019;44(2):239–49. | Not severe |
| Park SC, Ryu JN, Oh SJ, Cha YJ. Cross training effects of non-paralytic dorsiflexion muscle strengthening exercise on paralytic dorsiflexor muscle activity, gait ability, and balancing ability in patients with chronic stroke: A randomized, controlled, pilot trial. Journal of Musculoskeletal Neuronal Interactions. Januar 2021;21(1):51–8. | Not severe |
| Patel K, Murugan S, Limbasiya RD. A Study on the Effects of Kinesio Taping on Planter Flexor Spasticity, Balance and Functionality in Chronic Stroke Condition. Indian Journal of Physiotherapy & Occupational Therapy. Oktober 2019;13(4):62–7. | Wrong publication |
| Pérez-de la Cruz S. Comparison between Three Therapeutic Options for the Treatment of Balance and Gait in Stroke: a Randomized Controlled Trial. International journal of environmental research and public health [Internet]. Januar 2021;18(2). Verfügbar unter: https://www.cochranelibrary.com/central/doi/10.1002/central/CN-02247444/full | stroke severity not defined, Wrong setting |
| Peters S, Klassen T, Schneeberg A, Dukelow S, Bayley M, Hill M, u. a. Step Number and Aerobic Minute Exercise Prescription and Progression in Stroke: A Roadmap. Neurorehabil Neural Repair. 2022;36(2):97–102. | Not severe |
| Pilla A, Trigili E, McKinney Z, Fanciullacci C, Malasoma C, Posteraro F, u. a. Robotic Rehabilitation and Multimodal Instrumented Assessment of Post-stroke Elbow Motor Functions—A Randomized Controlled Trial Protocol. Frontiers in Neurology [Internet]. Januar 2020;11. Verfügbar unter: https://www.embase.com/search/results?subaction=viewrecord&id=L633312857&from=export | stroke severity not defined |
| Plummer P PhD P, Zukowski LA PhD, Feld JA PhD P, Najafi B PhD. Cognitive-motor dual-task gait training within 3 years after stroke: A randomized controlled trial. Physiother Theory Pract. 2022;38(10):1329–44. | Not severe |
| Ranzani R, Lambercy O, Metzger JC, Califfi A, Regazzi S, Dinacci D, u. a. Neurocognitive robot-assisted rehabilitation of hand function: a randomized control trial on motor recovery in subacute stroke. Journal of neuroengineering and rehabilitation. Januar 2020;17(1):115. | Not severe |
| Ravindran A, Rieke JD, Zapata JDA, White KD, Matarasso A, Yusufali MM, u. a. Four methods of brain pattern analyses of fMRI signals associated with wrist extension versus wrist flexion studied for potential use in future motor learning BCI. PLoS One. 2021;16(8):e0254338. | Wrong population |
| RBR-2hth8p. Motion capture assessment in upper limb neurofunctional rehabilitation in patients with chronic stroke. http://www.who.int/trialsearch/Trial2.aspx?TrialID=RBR-2hth8p [Internet]. Januar 2019; Verfügbar unter: https://www.cochranelibrary.com/central/doi/10.1002/central/CN-02071069/full | stroke severity not defined  wrong language, wrong publication type |
| Renner CIE, Brendel C, Hummelsheim H. Bilateral Arm Training vs Unilateral Arm Training for Severely Affected Patients With Stroke: Exploratory Single-Blinded Randomized Controlled Trial. Archives of physical medicine and rehabilitation. Januar 2020;101(7):1120–30. | stroke severity not defined |
| Rocha LSO, Gama GCB, Rocha RSB, Rocha LB, Dias CP, Santos LLS, u. a. Constraint Induced Movement Therapy Increases Functionality and Quality of Life after Stroke. J Stroke Cerebrovasc Dis. 2021;30(6):105774. | stroke severity not defined |
| Rogers JM, Duckworth J, Middleton S, y, Steenbergen B, Wilson PH. Elements virtual rehabilitation improves motor, cognitive, and functional outcomes in adult stroke: evidence from a randomized controlled pilot study. Journal of neuroengineering and rehabilitation. Januar 2019;16(1):56. | Not severe |
| Rojek A, Mika A, Oleksy Ł, Stolarczyk A, Kielnar R. Effects of Exoskeleton Gait Training on Balance, Load Distribution, and Functional Status in Stroke: A Randomized Controlled Trial. Frontiers in Neurology [Internet]. Januar 2020;10. Verfügbar unter: https://www.embase.com/search/results?subaction=viewrecord&id=L630727708&from=export | Not severe |
| Sackley CM, Walker MF, Burton CR, Watkins CL, Mant J, Roalfe AK, u. a. An occupational therapy intervention for residents with stroke related disabilities in UK care homes (OTCH): cluster randomised controlled trial. BMJ. 5. Februar 2015;350(feb05 23):h468–h468. | Wrong setting |
| Sánchez-Sánchez M, Belda-Lois JM, Mena-del Horno S, Viosca-Herrero E, Gisbert-Morant B, Igual-Camacho C, u. a. Functional principal component analysis as a new methodology for the analysis of the impact of two rehabilitation protocols in functional recovery after stroke. J NeuroEngineering Rehabil. 2014;11(1):134. | Wrong setting |
| Sandberg K. Effects of early exercise in patients with moderate to severe stroke. Januar 2020; Verfügbar unter: http://clinicaltrials.gov/ct2/show/NCT04241952 | Not severe |
| Sandberg K, Kleist M, Wijkman M, Enthoven P. Effects of in-bed cycle exercise in patients with acute stroke: a randomized controlled trial. Archives of Rehabilitation Research and Clinical Translation. Januar 2020;2(4). | Not severe |
| Sade I, Çekmece Ç, İnanir M, Selçuk B, Dursun N, Dursun E. The Effect of Whole Body Vibration Treatment on Balance and Gait in Patients with Stroke. Archives of Neuropsychiatry / Noropsikiatri Arsivi. Dezember 2020;57(4):308–11. | stroke severity not defined |
| Salazar AP, Cimolin V, Schifino GP, Rech KD, Marchese RR, Pagnussat AS. Bi-cephalic transcranial direct current stimulation combined with functional electrical stimulation for upper-limb stroke rehabilitation: A double-blind randomized controlled trial. Annals of physical and rehabilitation medicine. Januar 2020;63(1):4–11. | stroke severity not defined |
| Saleh MSM, Rehab NI, Aly SMA. Effect of aquatic versus land motor dual task training on balance and gait of patients with chronic stroke: A randomized controlled trial. NeuroRehabilitation. April 2019;44(4):485–92. | stroke severity not defined |
| Salehi Dehno N, Kamali F, Shariat A, Jaberzadeh S. Unilateral Strength Training of the Less Affected Hand Improves Cortical Excitability and Clinical Outcomes in Patients With Subacute Stroke: A Randomized Controlled Trial. Archives of Physical Medicine and Rehabilitation [Internet]. Januar 2021; Verfügbar unter: https://www.embase.com/search/results?subaction=viewrecord&id=L2010985696&from=export | No subanalysis |
| Schinkel-Ivy A, Huntley AH, Aqui A, Mansfield A. Does Perturbation-Based Balance Training Improve Control of Reactive Stepping in Individuals with Chronic Stroke? Journal of Stroke & Cerebrovascular Diseases. April 2019;28(4):935–43. | Not severe |
| Sengar S, Raghav D, Verma M, Alghadir AH, Iqbal A. Efficacy of dual-task training with two different priorities instructional sets on gait parameters in patients with chronic stroke. Neuropsychiatric Disease and Treatment. Januar 2019;15:2959–69. | Wrong setting |
| Sentandreu-Mañó T, Tomás JM, Ricardo Salom Terrádez J. A randomised clinical trial comparing 35 Hz versus 50 Hz frequency stimulation effects on hand motor recovery in older adults after stroke. Sci Rep. 2021;11(1):9131. | Not severe |
| Seo NJ, Woodbury ML, Bonilha L, Ramakrishnan V, Kautz SA, Downey RJ, u. a. TheraBracelet Stimulation During Task-Practice Therapy to Improve Upper Extremity Function After Stroke: A Pilot Randomized Controlled Study. Physical Therapy. März 2019;99(3):319–28. | stroke severity not defined |
| Serra MC, Hafer-Macko CE, Robbins R, O’Connor JC, Ryan AS. Randomization to Treadmill Training Improves Physical and Metabolic Health in Association With Declines in Oxidative Stress in Stroke. Arch Phys Med Rehabil. 2022;103(11):2077–84. | Wrong setting |
| Serrezuela RR, Quezada MT, Zayas MH, ez, Pedron AM, Hermosilla DM, u. a. Robotic therapy for the hemiplegic shoulder pain: a pilot study. Journal of neuroengineering and rehabilitation. Januar 2020;17(1):54. | stroke severity not defined |
| Sheehy L, Taillon-Hobson A, Sveistrup H, Bilodeau M, Yang C, Finestone H. Sitting Balance Exercise Performed Using Virtual Reality Training on a Stroke Rehabilitation Inpatient Service: A Randomized Controlled Study. PM and R. Januar 2020;12(8):754–65. | stroke severity not defined |
| Shibata T, Urata A, Kawahara K, Furuya K, Ishikuro K, Hattori N, u. a. Therapeutic Effects of Diagonal-Transcranial Direct Current Stimulation on Functional Recovery in Acute Stroke: A Pilot Study. Journal of Stroke and Cerebrovascular Diseases [Internet]. Januar 2020;29(10). Verfügbar unter: https://www.embase.com/search/results?subaction=viewrecord&id=L2006992401&from=export | stroke severity not defined |
| Shorter AL, Richardson JK, Finucane SB, Joshi V, Gordon K, Rouse EJ. Characterization and clinical implications of ankle impedance during walking in chronic stroke. Sci Rep. 2021;11(1):16726. | Wrong intervention |
| Simonelli M, Ruoppolo G, Iosa M, Morone G, Fusco A, Grasso MG, u. a. A stimulus for eating. The use of neuromuscular transcutaneous electrical stimulation in patients affected by severe dysphagia after subacute stroke: a pilot randomized controlled trial. Neurorehabilitation. Januar 2019;44(1):103–10. | stroke severity not defined |
| Simpson D, Ehrensberger M, Horgan F, Blake C, Roberts D, Broderick P, u. a. Unilateral dorsiflexor strengthening with mirror therapy to improve motor function after stroke: A pilot randomized study. Physiotherapy Research International. Oktober 2019;24(4):N.PAG-N.PAG. | stroke severity not defined |
| Sivaramakrishnan A, Madhavan S. Combining transcranial direct current stimulation with aerobic exercise to optimize cortical priming in stroke. Appl Physiol Nutr Metab. 2021;46(5):426–35. | Wrong intervention |
| Stephanie Hyeyoung Lee, Won-Seok Kim, Jihong Park, Junsik Kim, Nam-Jong Paik, Lee SH, u. a. Effects of anodal transcranial direct current stimulation over the contralesional hemisphere on motor recovery in subacute stroke patients with severe upper extremity hemiparesis: Study protocol for a randomized controlled trial. Medicine. April 2020;99(14):1–6. | stroke severity not defined |
| Stoykov ME, King E, David FJ, Vatinno A, a, Fogg L, u. a. Bilateral motor priming for post stroke upper extremity hemiparesis: A randomized pilot study. Restorative neurology and neuroscience. Januar 2020;38(1):11–22. | Wrong setting |
| Straudi S, Baroni A, Mele S, Craighero L, Manfredini F, Lamberti N, u. a. Effects of a Robot-Assisted Arm Training Plus Hand Functional Electrical Stimulation on Recovery After Stroke: A Randomized Clinical Trial. Archives of Physical Medicine & Rehabilitation. Februar 2020;101(2):309–16. | stroke severity not defined |
| Sui YF, Tong LQ, Zhang XY, Song ZH, Guo TC. Effects of paired associated stimulation with different stimulation position on motor cortex excitability and upper limb motor function in patients with cerebral infarction. J Clin Neurosci. 2021;90:363–9. | Wrong intervention |
| Sukumaran S, Sivadasan S, Sakunthala PT, T, on V, Sarma SP. „Sequential multimodality stimulation“ for post-stroke-hemineglect: Feasibility and outcome in a pilot randomized controlled trial. Journal of clinical neuroscience : official journal of the Neurosurgical Society of Australasia. Januar 2020;71:108–12. | Not severe |
| Swank C, Trammell M, Callender L, Bennett M, Patterson K, Gillespie J, u. a. The impact of a patient-directed activity program on functional outcomes and activity participation after stroke during inpatient rehabilitation-a randomized controlled trial. Clinical rehabilitation. Januar 2020;34(4):504–14. | Not severe |
| Tamburella F, Moreno JC, Herrera Valenzuela DS, Pisotta I, a, Iosa M, u. a. Influences of the biofeedback content on robotic post-stroke gait rehabilitation: electromyographic vs joint torque biofeedback. Journal of neuroengineering and rehabilitation. Januar 2019;16(1):95. | Wrong setting |
| Tanaka N, Matsushita S, Sonoda Y, Maruta Y, Fujitaka Y, Sato M, u. a. Effect of Stride Management Assist Gait Training for Poststroke Hemiplegia: A Single Center, Open-Label, Randomized Controlled Trial. Journal of Stroke & Cerebrovascular Diseases. Februar 2019;28(2):477–86. | stroke severity not defined |
| Tavares Aguiar L, Nadeau S, Rodrigues Britto R, Fuscaldi Teixeira-Salmel L, Caetano Martins J, Ribeiro Samora GA, u. a. Effects of aerobic training on physical activity in people with stroke: A randomized controlled trial. NeuroRehabilitation. März 2020;46(3):391–401. | stroke severity not defined, outpatient |
| Tirupatamma NL, Kameshwari G, Kumari VS, Madhavi K. To Know the Effectiveness of Rocker Board Training Programe on Trunk Balance and Gait in Subjects with Stroke. Indian Journal of Physiotherapy & Occupational Therapy. April 2019;13(2):236–41. | stroke severity not defined |
| Tomida K, Sonoda S, Hirano S, Suzuki A, Tanino G, Kawakami K, u. a. Randomized Controlled Trial of Gait Training Using Gait Exercise Assist Robot (GEAR) in Stroke Patients with Hemiplegia. Journal of Stroke & Cerebrovascular Diseases. September 2019;28(9):2421–8. | Not severe |
| Tramontano M, Morone G, Palomba A, De Angelis S, Mercuro A, Caltagirone C, u. a. Effectiveness of a sensor-based technology in upper limb motor recovery in post-acute stroke neurorehabilitation: a randomized controlled trial. Journal of biological regulators and homeostatic agents. Januar 2020;34(5):165‐174. Technology in Medicine. | stroke severity not defined |
| Tsubasa Mitsutake, Maiko Sakamoto, Etsuo Horikawa. The effects of electromyography-triggered neuromuscular electrical stimulation plus tilt sensor functional electrical stimulation training on gait performance in patients with subacute stroke: a randomized controlled pilot trial. International Journal of Rehabilitation Research. Dezember 2019;42(4):358–64. | stroke severity not defined |
| Umay E, Yilmaz V, Gundogdu I, Ozturk E, Gurcay E, Karaahmet O, u. a. What Happens to Swallowing Muscles after Stroke?: a Prospective Randomized Controlled Electrophysiological Study. Neurology India. Januar 2019;67(6):1459‐1466. | stroke severity not defined |
| Unal A, Altug F, Tikac G, Cavlak U. Effectiveness of matrix-rhythm therapy on increased muscle tone, balance and gait parameters in stroke survivors: a single-blinded, randomized, controlled clinical trial. Acta Neurol Belg. 2021;121(3):689–99. | Wrong setting |
| Vanroy C, Vanl, ewijck Y, Cras P, Truijen S, Vissers D, u. a. Does a cycling program combined with education and followed by coaching promote physical activity in subacute stroke patients? A randomized controlled trial. Disability & Rehabilitation. Februar 2019;41(4):413–21. | Not severe |
| Vaz L, Froes K, Almeida J, Filho JO, Pinto EB, Dias C. Inspiratory Muscle Training On The Activities Of Daily Living And Quality Of Live After Stroke. Archives of Physical Medicine and Rehabilitation. Januar 2019;100(12):e185. | Wrong publication type, no ful-text available |
| Vaz LO, Almeida JC, Froes KSDSO, Dias C, Pinto EB, Oliveira-Filho J. Effects of inspiratory muscle training on walking capacity of individuals after stroke: A double-blind randomized trial. Clin Rehabil. 2021;35(9):1247–56. | Not severe |
| Viktorisson A, Andersson EM, Lundström E, Sunnerhagen KS. Levels of physical activity before and after stroke in relation to early cognitive function. Sci Rep. 2021;11(1):9078. | No subgroups |
| Volpe BT, Lynch D, Rykman-Berland A, Ferraro M, Galgano M, Hogan N, u. a. Intensive Sensorimotor Arm Training Mediated by Therapist or Robot Improves Hemiparesis in Patients With Chronic Stroke. Neurorehabil Neural Repair. Mai 2008;22(3):305–10. | Wrong setting |
| Wall A, Borg J, Vreede K, Palmcrantz S. A randomized controlled study incorporating an electromechanical gait machine, the Hybrid Assistive Limb, in gait training of patients with severe limitations in walking in the subacute phase after stroke. PLoS ONE. Januar 2020;15(2):e0229707. | stroke severity not defined |
| Wall A, Borg J, Palmcrantz S. Self-perceived functioning and disability after randomized conventional and electromechanically-assisted gait training in subacute stroke: A 6 months follow-up. NeuroRehabilitation. Januar 2019;45(4):501–11. | Follow-up |
| Waddell KJ, Patel MS, Clark K, Harrington TO, Greysen SR. Leveraging insights from behavioral economics to improve mobility for adults with stroke: Design and rationale of the BE Mobile clinical trial. Contemp Clin Trials. 2021;107:106483. | Wrong setting |
| Wang HY, Zhu CH, Liu DS, Wang Y, Zhang JB, Wang SP, u. a. Rehabilitation training improves cognitive disorder after cerebrovascular accident by improving BDNF Bcl-2 and Bax expressions in regulating the JMK pathway. Eur Rev Med Pharmacol Sci. 2021;25(10):3807–21. | stroke severity not defined |
| Wang J, Zhang Y, Chen Y, Li M, Yang H, Chen J, u. a. Effectiveness of Rehabilitation Nursing versus Usual Therapist-Led Treatment in Patients with Acute Ischemic Stroke: A Randomized Non-Inferiority Trial. Clin Interv Aging. 2021;16:1173–84. | Not severe |
| Watanabe H, Marushima A, Kadone H, Ueno T, Shimizu Y, Kubota S, u. a. Effects of Gait Treatment With a Single-Leg Hybrid Assistive Limb System After Acute Stroke: A Non-randomized Clinical Trial. Frontiers in Neuroscience [Internet]. Januar 2020;13. Verfügbar unter: https://www.embase.com/search/results?subaction=viewrecord&id=L630798907&from=export | Not severe, wrong study design |
| Wei J, Zhu X, Xia L, Zhao Y, Yang G, Han Q, u. a. Intermittent pneumatic compression combined with rehabilitation training improves motor function deficits in patients with acute cerebral infarction. Acta Neurol Belg. 2021;121(6):1561–6. | Not severe |
| Wei YY, Koh CL, Hsu MJ, Lo SK, Chen CH, Lin JH. Effects of Transcranial Direct Current Stimulation Combined With Neuromuscular Electrical Stimulation on Upper Extremity Motor Function in Patients With Stroke. Am J Phys Med Rehabil. 2022;101(2):145–51. | Not severe |
| Widmer M, Held JPO, Wittmann F, Valladares B, Lambercy O, Sturzenegger C, u. a. Reward During Arm Training Improves Impairment and Activity After Stroke: A Randomized Controlled Trial. Neurorehabil Neural Repair. 2022;36(2):140–50. | Not severe |
| Wu WX, Zhou CY, Wang ZW, Chen GQ, Chen XL, Jin HM, u. a. Effect of Early and Intensive Rehabilitation after Ischemic Stroke on Functional Recovery of the Lower Limbs: A Pilot, Randomized Trial. Journal of Stroke & Cerebrovascular Diseases. Mai 2020;29(5):N.PAG-N.PAG. | Not severe |
| Xie H, Liang H, Chien JH. Different types of plantar vibration affect gait characteristics differently while walking on different inclines. PeerJ. 2023;11:e14619. | Wrong population |
| Yang X hui, Liu B, Ouyang B si. Effect of acupuncture combined with rehabilitative training on neural functional recovery of stroke patients during recovery phase: a randomized controlled trial. World Journal of Acupuncture - Moxibustion. Dezember 2014;24(4):17–23. | Wrong intervention |
| Yao X, Cui L, Wang J, Feng W, Bao Y, Xie Q. Effects of transcranial direct current stimulation with virtual reality on upper limb function in patients with ischemic stroke: a randomized controlled trial. Journal of neuroengineering and rehabilitation. Januar 2020;17(1):73. | Not severe |
| Yen HC, Chen WS, Jeng JS, Luh JJ, Lee YY, Pan GS. Standard early rehabilitation and lower limb transcutaneous nerve or neuromuscular electrical stimulation in acute stroke patients: a randomized controlled pilot study. Clinical Rehabilitation. August 2019;33(8):1344–54. | Not severe |
| Yeon-Gyo NAM, Jin Woo PARK, Ho Jun LEE, Ki Yeun NAM, Myong Ryol CHOI, Chang Seon YU, u. a. Further Effects of Electromechanically Assisted Gait Trainer (exowalk®) in Patients with Chronic Stroke: A Randomized Controlled Trial. Journal of Rehabilitation Medicine (Stiftelsen Rehabiliteringsinformation). September 2020;52(9):1–7. | stroke severity not defined |
| Yue S, Jiang X, Wong T. Effects of a nurse‐led acupressure programme for stroke patients in China. Journal of Clinical Nursing. April 2013;22(7–8):1182–8. | Wrong intervention |
| Yamamoto S, Motojima N, Kobayashi Y, Osada Y, Tanaka S, Daryabor A. Ankle-foot orthosis with an oil damper versus nonarticulated ankle-foot orthosis in the gait of patients with subacute stroke: a randomized controlled trial. J Neuroeng Rehabil. 2022;19(1):50. | stroke severity not defined |
| Yan LL, Gong E, Gu W, Turner EL, Gallis JA, Zhou Y, u. a. Effectiveness of a primary care-based integrated mobile health intervention for stroke management in rural China (SINEMA): A cluster-randomized controlled trial. PLoS Med. 2021;18(4):e1003582. | Wrong setting |
| Yeh IL, Holst-Wolf J, Elangovan N, Cuppone AV, Lakshminarayan K, Cappello L, u. a. Effects of a robot-aided somatosensory training on proprioception and motor function in stroke survivors. J Neuroeng Rehabil. 2021;18(1):77. | stroke severity not defined |
| Yoon HS, Cha YJ, You JSH. The effects of dynamic core-postural chain stabilization on respiratory function, fatigue and activities of daily living in subacute stroke patients: A randomized control trial. NeuroRehabilitation. Januar 2020;47(4):471–7. | stroke severity not defined |
| Yoon H, Cha Y, You J. Effects of dynamic core-postural chain stabilization on diaphragm movement, abdominal muscle thickness, and postural control in patients with subacute stroke: a randomized control trial. NeuroRehabilitation. Januar 2020;46(3):381‐389. | Not severe |
| Young HJ, van Wijck F. Upper-limb therapy for stroke survivors with severely-limited arm function: analysis of participants’ function and goal attainment following an augmented intervention. Physiotherapy. Januar 2020;107:e203. | Wrong publication type |
| Yu D, Yang Z, Lei L, Chaoming N, Ming W. Robot-Assisted Gait Training Plan for Patients in Poststroke Recovery Period: A Single Blind Randomized Controlled Trial. Biomed Res Int. 2021;2021:5820304. | stroke severity not defined |
| Yun N, Joo MC, Kim SC, Kim MS. Robot-assisted gait training effectively improved lateropulsion in subacute stroke patients: a single-blinded randomized controlled trial. European journal of physical and rehabilitation medicine. Januar 2018;54(6):827–36. | Not severe |
| Zhang H yan, Li P fang. Observation on efficacy of acupuncture combined with rehabilitation training for post-stroke balance disorders. World Journal of Acupuncture - Moxibustion. Juni 2014;24(2):25–9. | Wrong intervention |
| Zhao J, Chau JPC, Chan AWK, Meng Q, Choi KC, Xiang X, u. a. Tailored Sitting Tai Chi Program for Subacute Stroke Survivors: A Randomized Controlled Trial. Stroke. 2022;53(7):2192–203. | Wrong intervention |
| Zheng Y, Liu G, Yu L, Wang Y, Fang Y, Shen Y, u. a. Effects of a 3D-printed orthosis compared to a low-temperature thermoplastic plate orthosis on wrist flexor spasticity in chronic hemiparetic stroke patients: a randomized controlled trial. Clinical rehabilitation. Januar 2020;34(2):194–204. | stroke severity not defined |
| Zheng Y, Mao M, Cao Y, Lu X. Contralaterally controlled functional electrical stimulation improves wrist dorsiflexion and upper limb function in patients with early-phase stroke: A randomized controlled trial. Journal of rehabilitation medicine. Januar 2019;51(2):103–8. | stroke severity not defined |
| Zhuang JY, Ding L, Shu BB, Chen D, Jia J. Associated Mirror Therapy Enhances Motor Recovery of the Upper Extremity and Daily Function after Stroke: A Randomized Control Study. Neural Plast. 2021;2021:7266263. | Not severe |
| Acute stroke rehabilitation for gait training with cyborg type robot Hybrid Assistive Limb: a pilot study. Journal of the neurological sciences. Januar 2019;404:11‐15. | Not severe |
| Constraint-induced movement therapy in the rehabilitation of hemineglect after a stroke. Revista de neurologia. Januar 2020;70(4):119‐126. | Wrong language |

**Supplement S3 – Funding sources of the included studies**

| **study** | **Funding**  **(Y/N/NI)** | **Source of funding** |
| --- | --- | --- |
| AVERT Trail Collaboration Group, 2015, Bernhardt et al., 2020, Cain et al., 2022 | Y | National Health and Medical Research Council (NHMRC) of Australia (grant numbers 386201 and 1041401),Chest Heart and Stroke Scotland (Res08/A114), Northern Ireland Chest Heart and Stroke, Singapore Health (SHF/FG401P/2008), the UK Stroke Association (TSA2009/09), and the UK National Institute of Health Research (HTA Project 12/01/16). NHMRC fellowship funding was provided to AGT (1042600), HD (336102), and JB (1058635,1154904 and National Heart Foundation (G04M1571)), JB also received fellowship funding from the Australia Research Council (0991086) and the National Heart Foundation. A.Thrift received NHMRC fellowship funding (1042600). H. Dewey received NHMRC fellowship funding (336102) C. Reid received NHMRC fellowship funding (1136372). V. Srikanth received NHMRC fellowship funding (1061453). |
| Di Lauro et al., 2003 | N |  |
| Bai et al., 2014 | Y | National Key Technologies Program ‘The Research of Three-Stage Rehabilitation Program after Acute Cerebral Vascular Disease’ in the 10th 5-year-plan (Grant No. 2001BA703B21) |
| Katz-Leurer et al., 20003 | Y | partiality supported by the Chief Scienti.c Ministry of Health Jerusalem, Israel |
| Kwakkel et al., 1999*, 2002a, 2002 b | Y | grant from the Netherlands Heart Foundation (reference 93.134). |
| Rahayu et al., 2020* | N |  |
| An et al., 2020 | Y | Korea Health Technology R&D Project through the Korea Health Industry Development Institute (KHIDI), funded by the Ministry of Health & Welfare, Republic of Korea (grant no.: HI15C1529) |
| Bagley et al., 2005 | Y | Northern and Yorkshire NHS Executive research funding. |
| Brunelli et al., 2019* | NI |  |
| Calabró et al., 2015* | N |  |
| Chang et al., 2012* | Y | Korea Science and Engineering Foundation (#M10644000022-06N4400-02210) and Samsung Biomedical Research Institute (#SBRI C-A7-407-1), and Insung Research Foundation, Republic of Korea. |
| Chen et al., 2011* | Y | Tzu Chi General Hospital [grant number TCRD 99-20 Research Plan]. |
| Cui et al., 2022 | Y | Sichuan Province Pharmaceutical Administration (Grant No. 2014B064), the Key R&D Program of Sichuan Province (No.2020YFS0415). |
| Francesschini et al., 2009 | NI |  |
| Lee et al., 2020* | Y | supported by the 2019 Inje University research grant. |
| Lincoln et al., 1999 | Y | National Health Service (NHS) Executive, NHS Research and Development Programme on Cardiovascular Disease and Stroke |
| Louie et al., 2021 | Y | Heart and Stroke Foundation of Canada Grant in Aid (G-15-0009030) and Canadian Institutes of Health Research Foundation Grant (FDN 143340) |
| Logan et al., 2022 | Y | National Institute for Health Research (NIHR) (ICA-CDRF- 2015-01-044). |
| Ochi et al., 2015* | Y | Grant-in-Aid for Scientific Research (A), Japan Society for the  Promotion of Science KAKENHI Grant Number 15200044 (Partially) |
| Rosewilliam et al., 2012 | Y | Funded by Action Medical Research and Barnwood House Trust (AP0993) |
| Shao et al., 2023 | NI |  |
| Tang et al., 2014* | Y | National Natural Science Foundation of China (No. 30973167), China Postdoctoral Science Foundation (No. 2011M501301, No. 2012T50711), Postdoctoral Science Foundation of Hunan Province, and Postdoctoral Science Foundation of Central South University |
| Thimabut et al., 2022 | ???? | Chulalongkorn University for providing the doctoral  scholarship under H.M. the King Bhumibhol Adulyadej’s 72^nd^ Birthday Anniversary Scholarship and Overseas Research Experience Scholarship for Graduate Student of Chulalongkorn University (CU5974857030) |
| Zheng et al., 2018* | Y | National Science Foundation (grant number 81772447) and the Guangzhou Science and Technology Program key projects (grant number 201704020140) |
| Choi et al., 2021* | Y | Korea Health Technology R&D Project through the Korea Health Industry Development Institute (KHIDI), funded by the Ministry of Health & Welfare, Republic of Korea (grant number: HI18C2383) |
| Guo et al., 2018* | NI |  |
| Kim et al., 2022 | N |  |
| Kim et al., 2020 | Y | National Research Foundation of Korea (grant number  2017R1D1A1B03027947) |
| Rodrigues et al., 2017* | Y | Coordination of Improvement of Higher Level Personnel–CAPES aswell as the Cardinal Hill Rehabilitation Hospital Endowed Chair in Stroke and Spinal Cord Injury Rehabilitation (0705129700). |
| Sim and Kwon, 2022 | N |  |

Abbreviations: Y-yes, N- no, NI-no information

**Supplement S4 - Outcomes according to ICF domains**

| **Body function** | **Activity** | | **Participation** |
| --- | --- | --- | --- |
| Biomarker  BDNF Biomarker  Bone representationX-Ray  Strength  Medical Research Council Scale  Dynamometer  Cardiorespiratory capacity  Peak VO²  Tonus  Modified Ashworth scale  Cognition  Raven´s Coloured Progressive Matrices  Mini-Mental-State-Exam  Montreal Cognitive Asessment  Neglect  Line bisection test  Motor-Free Visual Perception Test - Vertical  Alert test  Behavioral Inattention Test  Continence  International Consultation on Incontinence Questionnaire-Short Form  Overactive Bladder Symptom Score  Urodynamic outcome  Emotion  Hospital anxiety and depression scale  Sensorimotor Function  Fugl-Meyer Assessment  Fugl-Meyer Assessment upper extremity  head-tracking sensor data  National Institute of Health Stroke Scale  Scandinavian Stroke Scale  Motricity Index  Pusher Syndrome  Burke Lateropulsion Scale  Pain  Visual Analogue Scale  Fatigue  Visual Analogue Scale  Range of Motion  Goniometer | Activities of daily living  Barthel Index  (Korean) Modified Barthel Index  Nottingham extended Activities of daily living Scale  Frenchay activities Index  Sickness Impact Profile  Edmands ADL Index  Functional independence/mobility  Modified Rankin Scale  Rivermead Mobility Index  Functional Independence Measure  Neglect  Catherine Borgego Scale  Brain activity  (resting state) fMRI  Balance  Balancia 2.0 program  Berg Balance Scale  Brunel Balance Assessment  Postural Assessment Scale for Stroke  Motor-Assessment-Scale  Trunk-Control-Test  Gait endurance  GAITRite system  6-minute Walk Test  Walking capacity  Functional ambulatory category  Walking speed  5 Meter Walk Test  Arm/upper limb function  Motor Activity Log  Action research Arm Test  Nine hole peg test | | General Health  Nottingham health profile Part 1  Stroke and Aphasia Quality of Life Scale-39  EuroQol – 5 Dimension – 5 Level  Medical Outcomes Short-Form 36  Depression  Patient Health Questionnaire  Stroke Aphasia Depression  Questionnaire-10 |
| Abbreviations | | | |
| BBA | | Brunel Balance Assessment | |
| BBS | | Berg Balance Scale | |
| BDNF Biomarker | | Brain-derived neurotrophic factor - Biomarker | |
| BI | | Barthel Index | |
| CBS | | Catherine Borgego Scale | |
| EQ-5D 5L | | EuroQol – 5 Dimension – 5 Level | |
| FAC | | Functional Ambulation Classification | |
| FMA | | Fugl-Meyer Assessment | |
| FMA UE | | Fugl-Meyer Assessment upper extremity | |
| fMRI | | Functional magnetic resonance images | |
| HTSD | | head-tracking sensor data | |
| ICIQ-SF | | International Consultation on Incontinence Questionnaire-Short Form | |
| K-mBi | | Korean Modified Barthel Index | |
| LBT | | Line bisection Test | |
| mBI | | Modified Barthel Index | |
| MAL | | Motor Activity Log | |
| MoCA | | Montreal Cognitive Asessment | |
| MVPT-V | | Motor-Free Visual Perception Test Vertical | |
| PASS | | Postural Assessment Scale for Stroke | |
| PHQ-9 | | Patient Health Questionnaire | |
| OBASS | | Overactive Bladder Symptom Score | |
| RMI | | Rivermead Mobility Index | |
| rs fMRI | | Resting state functional magnetic resonance imaging | |
| SADQ-10 | | Stroke Aphasia Depression Questionnaire-10 | |
| SF-36 | | Medical Outcomes Short-Form 36 | |
| 6MWT | | 6-minute Walk Test | |
|  | | Balancia 2.0 program  GAITRite system  Urodynamic outcome | |

**Supplement S5– Grade judgement**

| **Judging the Quality of Evidence according to the GRADE Handbook** (1,2) | | |
| --- | --- | --- |
| **Grade Domain** | **Judgment**  The restriction is not enough for grading after discussion with the team | **Concerns about certainty domains** |
| ***Outcome: Functional impairment in global early mobilization without electrical supportive devices within 24 hours*** | | |
| Limitations in the design and implementation of available studies suggest a high likelihood of bias** | Two studies (3,4) referred to the outcome independency with various scales.  One out of three trials had a low risk of bias and had the largest study population (291 participants, (4)). trial with 60 participants showed a high risk of bias (3).  Due to the number of participants in the study with a low risk of bias compared to the study with a high risk of bias with 60 participants, the authors' overall assessment is rated as borderline severe. | Borderline serious, do not downgrade |
| Inconsistency of results | The study population across the studies did not vary in age. The mean age of the patients across all trials was approximately 68 years. All studies used global mobilization as an intervention. One study (4) started intervention after 24h of stroke onset with sitting and standing out-of-bed activities. Another study used intensive rehabilitative treatment mobilization exercises and rehabilitative nursing 24 hours post-stroke (3). The comparison groups varied between trials from usual care, including physical therapies (4) and not further described rehabilitative treatment (3).  The outcomes were assessed using different scales like Barthel Index(BI, (3)) and modified Rankin Scale (mRS (4)).  We judged the evidence to have no serious indirectness concerning participants, intervention, and outcomes but noted some variability in the intervention and outcome measure.  The direction and magnitude of the effect varied across different trials. One study showed no changes between groups (3). The other study favors the control intervention (4).  Inconsistency in the results and their direction had been found. The largest trial with a low risk of bias showed a small Confidence Interval (CI). Therefore, we judge the evidence to have serious inconsistency. | Serious, downgrade one level |
| Indirectness of evidence | The study's patients, intervention and comparators all provide direct evidence to the clinical question at hand. All interventions included any kind of rehabilitative intervention. The types of outcomes were assessed with different scales in different trials. We judge the evidence to have no serious indirectness but noted some variability in the intervention and outcome measure. | Not serious |
| Imprecision | The total number of participants 351 is below the required optimal information size (IOS). One study (3) showed no statistical significance between groups after intervention (p>0.7 at 180 days post-intervention, nothing was reported after intervention). In the early mobilisation trials, subgroup analysis for severe stroke patients favours usual care more than early mobilisation with an Odds Ratio of 0·35 (0·11–1·18) (4).  The threshold of 400 participants is not reached and the results are imprecise. We are also taking into consideration the risk of bias and the number of participants in the single studies. We, therefore, judge the evidence as not serious. | Not serious |
| Likelihood of publication bias | Our literature search was comprehensive, there were no unpublished studies in the registries, and the studies reported both positive and negative outcomes. Therefore, we had no serious concerns. | Not serious |
| Overall judgment | There is moderate quality of evidence for non-superiority of early mobilization within 24 hours compared to usual care. | ⊕⊕⊕🌕Moderate |
| ***Outcome:*** ***Basic ADL for neurodevelopmental interventions without electrical supportive devices*** | | |
| Limitations in the design and implementation of available studies suggest a high likelihood of bias** | Two studies (6,7) referred to the outcome of basic Activity of daily living (ADL) with the Barthel Index.  The trial (7) with the smallest number of participants (n=67) showed a high risk of bias. The second trial included 282 participants and had come concerns regarding methodology.  After discussion with the team, the restriction is not sufficient for downgrading. | Borderline serious, do not downgrade |
| Inconsistency of results | The direction and magnitude of the effect in basic ADL across the trials were conflicting. The smaller study with a high risk of bias showed a positive direction for the intervention group. The larger (6) trial could not show a between-group difference.  We grade the evidence inconstant and recommend downgrading one level due to different directions of effects and the number of participants.  The study population across the studies varied in age and stroke type. The mean age of the small trail is 59 years (7), and the large trail had a mean age of 73 years ranging from 64-91 years (6)The information about the type of stroke is given in trial with differentiation between infecting and other sources. Whereas the information in the other trial is defined with the ASPECT score (7). There are no systematic differences between different clinical settings. Differences in countries, Indonesia (7)), the and United Kingdom (6), had been detected.  One trial preferred Bobath as the neurodevelopmental intervention (6). It was very structured but not individually tailored. Besides Bobath, the second trial also used PNF, Rodd, Carr and Shepard, and CIMT (7). The intervention was judged as consistent but noted some variability. Both trials used usual care or routine physiotherapy, not further described. Both trials used Barthel Index to assess basic ADLs.  There are no serious concerns about inconsistency. | Serious, downgrade one level |
| Indirectness of evidence | The patients, intervention and comparators in the study all provide direct evidence at hand to the clinical question at hand. All interventions included any kind of rehabilitative intervention. The types of outcomes were assessed with different scales in different trials. We judge the evidence to have no serious indirectness but noted some variability in the intervention and outcome measure. | Not serious |
| Imprecision | With an overall number of 349 participants across trials, the required optimal OIS had not been reached.  In the large trial (6) no differences had been found between intervention and control group (post-intervention p=0.66, 3 months postintervention p=0.51, and 6 months postintervention p=0.65). The small trail showed wide CI post-stroke for control group (46.41 (37.77-55.04)) and intervention group (67 (58.99-75.94)) and a statistical significance between means (p=0.008). However, wide confidence intervals and appreciable benefits, and no difference between groups found Therefore, we had serious concerns regarding imprecision. | Serious, downgrade one level |
| Likelihood of publication bias | Our literature search was comprehensive, there were no unpublished studies in the registries, and the studies reported both positive and negative outcomes. Therefore, we had no serious concerns. | Not serious |
| Overall judgment | There is low quality of evidence for missing effects in neurodevelopmental interventions without electrical supportive devices compared to usual care in basic ADL in severe stroke patients. | ⊕⊕🌕🌕Low |
| ***Outcome:*** ***Basic ADL in interventions with neuromuscular electrical stimulation*** ***(NMES)*** | | |
| Limitations in the design and implementation of available studies suggest a high likelihood of bias** | Two studies (8,9) reported basic ADLs on the Barthel Index.  One trial (n=82) showed a high risk of Bias due to some concerns in the randomization process and high risk of bias in the selection of the reported results (8). The other trail trial) showed some concerns overall (9). Therefore, we judged the trials to have serious methodological limitations. | Borderline serious, do not downgrade |
| Inconsistency of results | The direction and magnitude of effect did not vary across the different trials. One trial (8) showed a small trend for the intervention group. In the second trial (9) differences between groups regarding the intervention group were found. We had no concerns according to the direction of the effect.  The study population varied in age with a mean age of 63 years (8) compared to 74 years (9). They also showed differences in the time post-stroke including participants in the early subacute (6 weeks (9)) and chronic (8 months post-stroke, (8)) phase. The setting for both trials are the same taking place in different countries like Great Britain (9) and China (8). Systematic differences could be detected.  Both trials used an NMES for the intervention group. The intervention was applied to various parts of the body of the participants. Surface electrodes had been used to stimulate the wrist and finger extensors (9) or for urinary incontinence (8). The control interventions were standardized upper limb therapy (9) and sham NMES (8). This may affect the consistency and was judged as serious. | Serious, downgrade one level |
| Indirectness of evidence | The patients, intervention and comparators in the study all provide direct evidence at hand to the clinical question at hand. All interventions included any kind of rehabilitative intervention. The types of outcomes were assessed with different scales in different trials. We judge the evidence to have no serious indirectness but noted some variability in the intervention and outcome measure. | Not serious |
| Imprecision | The required OIS was not reached due to the number of participants 172. One study detected a between-group difference (5.1 (2.8,7.2)) at week 10 (8). The other study showed a difference between groups for participants who are still alive (p=0.03).  We had borderline concerns regarding imprecision. | Serious, downgrade one level |
| Likelihood of publication bias | Our literature search was comprehensive, there were no unpublished studies in the registries, and the studies reported both positive and negative outcomes. Therefore, we had no serious concerns. | Not serious |
| Overall judgment | There is low quality of evidence that NMES might improve basic ADL compared to standard care and sham NMES in severe stroke patients. | ⊕⊕🌕🌕 Low |
| ***Outcome: Extended ADL in interventions with verticalization support*** | | |
| Limitations in the design and implementation of available studies suggest high a likelihood of bias** | Two studies (10,11) referred to the outcome of extended ADLs with the Edmans ADL Index for Stroke.  A trial with 140 participants and a high overall risk and one with 45 participants showed some concerns about the risk of bias (11). Due to the number of participants, the authors' overall assessment is rated as serious. | Serious, downgrade one level |
| Inconsistency of results | The direction and magnitude of the effect did not vary across trials. Both studies showed positive but no significant changes for the intervention group.  The study population across the studies did not vary in age. All studies used a standing frame as intervention. One study combined standing with sit-to-stand-repetition plus 15 min of usual physiotherapy (11). The comparison groups varied between trials from usual care (11) or physiotherapy with an available tilt table if required (10). The outcomes were assessed using the same scales in different trials.  There had been no inconsistency detected in the results and their direction. Therefore, we had no serious concerns about the inconsistency. | Not serious |
| Indirectness of evidence | The patients, intervention and comparators in the study all provide direct evidence at hand to the clinical question at hand. All interventions included any kind of rehabilitative intervention. The types of outcomes were assessed with the same scales in different trials. We judge the evidence to have no serious indirectness but noted some variability in the intervention and outcome measure. | Not serious |
| Imprecision | The total number of participants with 185 is less than the required IOS. One study showed no between-group difference (10). The other reported between-group differences with no significant effect at 29 weeks follow-up which could not persist over time (post-intervention: 0.41 (-1.13, 1.93); 15 weeks follow-up: 0.27 (-1.57, 2.11); 29 weeks follow-up: 0.00 (-2.12, 2.12) and 55 weeks follow-up: 0.92 [-1.50, 3.33] (11)).  The threshold of 400 participants is not reached and the results are imprecise. We, therefore, judge the evidence as serious. | Serious, downgrade one level |
| Likelihood of publication bias | Our literature search was comprehensive, there were no unpublished studies in the registries, and the studies reported both positive and negative outcomes. Therefore, we had no serious concerns. | Not serious |
| Overall judgment | There is low quality of evidence for no superiority of interventions with verticalization support compared to usual care in extended ADLs of participants with severe stroke. | ⊕⊕🌕🌕Low |
| ***Outcome:*** ***Balance skills in neurodevelopmental interventions*** | | |
| Limitations in the design and implementation of available studies suggest a high likelihood of bias** | Two studies (7,13) reported on balance ability with Berg Balance Scale.  Both showed a high risk of bias. One trial (7) had 67 participants and the second trial included 48 participants (13). Therefore, we judged the trials to have serious methodological limitations. | Serious, downgrade one level |
| Inconsistency of results | The direction and magnitude of the effect balance ability across the trials were in the same direction. The smaller study with a high risk of bias showed a positive direction for the intervention group. The larger trial also showed a positive trend for the intervention group.  The study population across the studies varied slightly in age and country. The mean age of one trail was 59 years (7) and the other trail had a mean age of 67 years (13). The information about the type of stroke was scored by the ASPECT score in one trial (7). There are no systematic differences between different clinical settings. Differences in countries, Indonesia (7), and China (13)had been detected.  Bobath with early sitting, standing and balance training (13) had been used as an intervention in one trial. It was very structured and well-tailored. The second trial also used Bobath and PNF, Rodd, Carr and Shepard as we, and(7). One trial used usual care (7), not further described. The second trial used a conventional Bobath approach (13). Both trials used the Berg Balance Scale to assess balance. Even if both interventions rely on Bobath the described intervention and control differ from one another.  We grade the evidence consistently. | Serious, downgrade one level |
| Indirectness of evidence | The patients, intervention and comparators in the study all provide direct evidence at hand to the clinical question at hand. All interventions included any kind of rehabilitative intervention. The types of outcomes were assessed with different scales in different trials. We judge the evidence to have no serious indirectness but noted some variability in the intervention and outcome measure. | Not serious |
| Imprecision | With an overall number of 115 participants across trials, the required optimal OIS had not been reached.  In one trial (13) large effects at 4 (F (1, 46) = 35.4, ŋ^2^ = 0.435, p < .001) and 8 weeks post  Post-intervention (F (1, 46) = 73.1, ŋ^2^ = 0.614, p < .001) had been detected. The other trial (7) showed wide CI post-stroke for control group (Mean (95% CI) 17.16 (12.62–21.69)) and intervention group (28.38 (21.74–35.01)). They showed a significant group difference of p= 0.016. Therefore, we had serious concerns regarding imprecision. | Serious, downgrade one level |
| Likelihood of publication bias | Our literature search was comprehensive, there were no unpublished studies in the registries, and the studies reported both positive and negative outcomes. Therefore, we had no serious concerns. | Not serious |
| Overall judgment | There is very low quality of evidence for a statistically significant, small but clinically relevant effect (14) of neurodevelopmental interventions to improve balance in two studies (7,13) compared to usual care and a conventional Bobath approach. | ⊕🌕🌕🌕 Very Low |
| ***Outcome:*** ***Walking capacity in robotic-assisted gait training*** | | |
| Limitations in the design and implementation of available studies suggest a high likelihood of bias** | Three trials (17–19) reported on walking capacity using the Functional Ambulatory Categories.  The study with 48 participants (18) showed a high risk of bias. Another study with 26 participants had some concerns overall (17) and the last study with 26 patients showed a low overall risk (19). Therefore, we judged the trials to have borderline concerns. | Borderline serious, do not downgrade |
| Inconsistency of results | The direction and magnitude of effect vary across the different trials. In favour of the interventions, a group had been found in one trial (18).  The study population across studies varied in the age of participants. The mean age of the patients across those studies ranged from 55,5±12 years (18) to 65,5±12,1 years (11). No systematic differences between different clinical settings could be detected. Studies had been conducted in different countries like Japan (17) ,Korea (18) and Canada (19). The different technological advances in the individual countries, influence study.  A robotic-assisted locomotor gait training (RAGT) on a body weight-supported treadmill (18) was used in one trial. Another one used a weight-bearing gait assistance robot (17).The third one used an Exoskeleton device (19). Therefore, we had no serious concerns about the directness of evidence concerning the interventions. Their control group differed slightly from using standard overground gait training (17), usual care based on the Bobath approach (18) and usual care with the time spent in physical upright positions, like standing or walking and counting steps during sessions (19). The outcome was assessed using the same scales in all trials.  We had serious concerns according to the inconsistency of the results based on the different reporting and directions of the effects. | Serious, downgrade one level |
| Indirectness of evidence | The patients, intervention and comparators in the study all provide direct evidence at hand to the clinical question at hand. All interventions included any kind of rehabilitative intervention. The types of outcomes were assessed with same scales in different trials. We judge the evidence to have no serious indirectness but noted some variability in the intervention and outcome measure. | Not serious |
| Imprecision | The total number of participants with 100 is less than the required OIS. One study detected significant between-group differences (p=0.02) in favour of the intervention group (17). No group differences had been detected in two other trials.  Regarding the IOS and the given effect sizes we had serious concerns regarding imprecision. | Serious, downgrade one level |
| Likelihood of publication bias | Our literature search was comprehensive, there were no unpublished studies in the registries, and the studies reported both positive and negative outcomes. Therefore, we had no serious concerns. | Not serious |
| Overall judgment | There is low quality of evidence for small a but significant effect in robotic-assisted gait training (RAGT) compared to conventional therapy and overground gait training in walking capacity in patients with severe stroke. | ⊕⊕🌕🌕 Low |
| ***Outcome:*** ***Motor function in robotic-assisted gait training*** | | |
| Limitations in the design and implementation of available studies suggesting high la ikelihood of bias** | Three trials referred to the outcome motor of function with the Fugl-Mayer-Assessment of the lower extremity (FMA-LE). The study with 48 participants (18) showed a high risk of bias. One study with 26 participants had some concerns (17) and the last study with 26 patients showed a low overall risk (19). Therefore, we judged the trials to have borderline methodological limitations. | Borderline serious, do not downgrade |
| Inconsistency of results | The direction and magnitude of effect did vary across different trials. No between-groups were shown in one trial (17). A positive effect for intervention group was found in the other trial (18).  No systematic differences for different clinical settings could be detected. Studies had been conducted in different countries like Japan (17), Canada (19)and Korea (18). Due to different technological advances in the individual countries, this could an influence on the study.  One study used robotic-assisted locomotor training on a body weight treadmill (18). Another trail triala weight-bearing gait assistance robot (17). The third one used an Exoskeleton device (19). Their control group differed slightly from using standard overground gait training (17),usual care (18) and usual care with the time spent in physical upright positions, like standing or walking and counting steps during sessions (19).The outcome was assessed using the same scales in all trials showed some concerns according to the inconsistency of the results through to the different directions of effect and slight differences in the intervention and control group. Therefore, we recommend downgrading one level. | Serious, downgrade one level |
| Indirectness of evidence | The patients, intervention and comparators in the study all provide direct evidence at hand to the clinical question at hand. All interventions included any kind of rehabilitative intervention. The types of outcomes were assessed with different scales in different trials. We judge the evidence to have no serious indirectness but noted some variability in the intervention and outcome measure. | Not serious |
| Imprecision | The total number of participants of 100 is less than the required OIS. One study showed no between-group differences (p>0.05) but a positive trend for both groups comparing pre- and post-test showed a positive trend (p<0.01) (18). Two studies did not show between-group differences (17,19).  Regarding the IOS and the missing effect sizes we had serious concerns regarding imprecision | Serious, downgrade one level |
| Likelihood of publication bias | Our literature search was comprehensive, there were no unpublished studies in the registries, and the studies reported both positive and negative outcomes. Therefore, we had no serious concerns. | Not serious |
| Overall judgment | There is low quality of evidence for no superiority of RAGT compared to conventional therapy and overground gait training in motor function of the lower limb in participants with severe strokes. | ⊕⊕🌕🌕Low |
| ***Outcome: Dexterity in highly intensive active interventions (without electric support)*** | | |
| Limitations in the design and implementation of available studies suggest high a likelihood of bias** | Two studies (6,12) referred to the outcome of motor function of the upper extremity with the Action Research Arm Test (ARAT).  A trial with 101 participants (12) and one with 282 participants showed some concerns about the risk of bias (6). Due to the number of participants, the authors' overall assessment is rated as borderline serious. | Borderline serious, do not downgrade |
| Inconsistency of results | The direction and magnitude of the effect varied across different trials. One study showed positive changes for the intervention group (12). The other study showed no effects (6).  The study population across the studies did not vary in age. All studies used global mobilization as an intervention with a high frequency. One study used additional individualized upper or lower extremity training by physical or occupational therapists for 30 min additionally to 1,5 h treatment per workday (12). One trial preferred Bobath as the neurodevelopmental intervention (2h, (6)). It was very structured but not individually tailored.  The comparison groups varied between trials from usual care or immobilization of the affected limb with an inflatable pressure splint in addition to a leg and arm rehabilitation and ADL training per day (12). The outcomes were assessed using the same scales in different trials.  Inconsistency in the results and their direction had been detected. Therefore, we had serious concerns about the inconsistency. | Serious, downgrade one level |
| Indirectness of evidence | The patients, intervention and comparators in the study all provide direct evidence at hand to the clinical question at hand. All interventions included any kind of rehabilitative intervention. The types of outcomes were assessed with different scales in different trials. We judge the evidence to have no serious indirectness but noted some variability in the intervention and outcome measure. | Not serious |
| Imprecision | The total number of participants with 383 is less than the required OIS. One study (12) showed between-group difference at week 20 with p<0.01 and week 26 p<0.001 for the intervention group arm training. The other study showed no between-group differences (post-intervention p=0.62; 6 months post-intervention p=0.55; (6)).  The threshold of 400 participants is not reached and the results are imprecise. We, therefore, judge the evidence as serious. | Serious, downgrade one level |
| Likelihood of publication bias | Our literature search was comprehensive, there were no unpublished studies in the registries, and the studies reported both positive and negative outcomes. Therefore, we had no serious concerns. | Not serious |
| Overall judgment | There is low quality of evidence for no superiority of highly intensive active interventions compared to restriction and usual care in dexterity of participants with severe stroke. | ⊕⊕🌕🌕Low |

Following outcomes only addressed once

**Spasticity**

In a study of patients who underwent a staged rehabilitation intervention compared to standard care for 90 min/day, twice/day, or 5 days/week for 3 months, the modified Ashworth scale (MAS) showed differences between groups, with lower MAS scores in the intervention group at M_6_ in the elbows, fingers and plantar flexors (p<0.05) and fingers and plantar flexors at M_3_ (p<0,05) compared to usual care and routine medication without participation in the rehabilitation intervention (20). The rehabilitation intervention consisted of different stages starting with passive movements and positioning of the affected limb by physiotherapy and engaging participants in early active exercise in bed, sitting, standing and balance training. This was followed by passive stretching of spastic muscles and strengthening antagonists while continuing with the rehabilitative treatment of walking, balance, and ADL training.

**Neglect**

Digital training with virtual reality for 30 min/day and 12 treatments over 4 weeks resulted in greater visual perception recovery, especially in terms of head rotation degree (24 participants, MD 51.17 95% CI (10.45), p=0.007) and velocity (24 participants, MD 23.73 95% CI (23.62), p=0.001), than did no digital training, as measured with head tracking sensor data and the Motor-Free Visual Perception Test (21). In another study comparing bimanual mirror therapy (BMT) with unimanual mirror therapy (UMT), the Line Bisection Test (LBT) and Star Cancelation Test (SCT) were used to assess the level of unilateral neglect (22). Both interventions were applied in five 30-min sessions over four weeks. The BMTs showed better outcomes in the SCT (p=0,02) and LBT (p=0,04) groups than in the UMT group.

**Cognitive function**

In a robotic verticalization study for 30 sessions and 30 min daily, compared with the same amount of time, physiotherapy-assisted verticalization training produced a difference between groups (p=0.03) measured by the Ravens Coloured Progressive Matrices (RCPM) (23).

**Sensorimotor function**

One study used thermal stimulation to move the paretic leg away from the hot stimulus compared to discussions for 20 min each, and at least 3 times/week showed significant differences between groups in favor of the intervention group in the FMA-lower extremity (FMA-LE) (week 4: p=0.003; week 6: p<0.001) (24). The FMA-LE was also used in a study comparing mirror therapy (MT) of the lower extremity to routine rehabilitation therapies (RRTs)(25). After 3 weeks of training for 30 min, five times a week, the FMA-LE improved significantly in both groups (MT: F = 24.845, P < 0.01; RRT: *F* = 11.628, *p* = 0.002) and showed a between-group difference with a greater effect on MT (*Z* = –4.526, *p* < 0.01).

**Walking capacity**

One study used BWS overground gait training with two 40-minute sessions, 5 days/week for 4 weeks, compared to conventional gait training for 4 weeks and showed a significant difference between groups as measured with the FAC (u=122.0, p<0.01) (26).

A study comparing slow to fast BWS overground gait training for 40 min/treatment, twice/day, and 5 days/week for 4 weeks showed a significant improvement in walking capacity (p=0.004) in the slow group, but no differences were reported between groups (27).

**Spatiotemporal gait parameters**

Underwater gait training compared to overground gait training for 12 weeks with a 30 min treatment twice a week showed a difference between groups in favor of the control group in step length difference (participants: 21, MD 9.28, 95% CI (8.08), p<0.05) (28).

**Walking speed**

A RAGT compared to a control group with physiotherapy and ground-level ambulatory training for 60 min, five sessions per week for 6 weeks did not significantly differ between groups at any of the time points of measurement: 15^th^ session, p= 0.14, 95% CI (-0.04 m/s to 0.28 m/s); and 30^th^ session, p=0.142, 95% CI (-0.07 m/s to 0.45 m/s) (29).

**Balance**

After four-channel FES was applied in 5-second gait cycles 5 times/week for 3 weeks, the hemiplegic leg, in addition to 2 h of standard care, was more strongly related to balance in the dual-channel FES group and placebo FES group, as measured with the Berg Balance Scale (BBS: 33 participants, MD 40.27, 95% CI (10.82), p ≤ 0.01) and the Postural Assessment Scale for Stroke (PASS: 33 participants, MD 29.63, 95% CI (5.92), p ≤ 0.01) (30). A study comparing the effect of strength training on the nonhemiplegic lower extremity compared to usual rehabilitation for 45 min five days a week showed a better outcome after 6 weeks in the BBS group than in the control group (adjusted mean difference: 6.83; 95% CI: 4.71-8.94) (31).

**Upper extremity function**

Compared with standard mirror therapy, multijoint mirror therapy for 30 min/treatment and 5 times/day for 4 weeks had superior effects on the quality of use of the upper extremity (participants: 21, MD 3.71 95% CI (5,72), p=0.04) for the participants in the intervention group (32).

**Independence in daily and social activities**

The use of the leg cycle ergometer for 30 min in a tailored program for 32 sessions over 8 weeks did not significantly differ between groups in terms of instrumental ADLs measured with the Frenchay Activities Index (33).

**Functional mobility**

The use of an Oswestry standing frame for 14 treatments lasting 26 min (IQR 20-30 min)/treatment, compared to 14 consecutive treatments, did not significantly differ from baseline to six weeks between the groups according to the Rivermead Motor Assessment (RMA) to assess functional mobility (p=0.03, median change; Intervention=0 (IQR 0-2); control=1 (IQR 0-2)) (34).

**Muscle Strength**

The use of an elastic dynamic sling to prevent subluxation of the affected arm, compared to the use of a Bobath sling for 8 weeks during the active time of day, did not significantly differ between groups (p=0,752) (35).

**Pusher Syndrom**

With the help of a whole-body tilt apparatus, lateral pulsion, measured with the Burke Lateropulsion Scale (BLS), was investigated for 30 min, twice a day, five days per week for three weeks compared to general postural training (36). The BLS score decreased significantly (p=0,002) in the intervention group (mean (SD) change–5.8 (1.2)) compared to the control group (mean change–4.2 (1.3)).

| **** Further guidelines for factor 1 (of 5) in a GRADE assessment: Going from assessments of risk of bias to judgments about study limitations for main outcomes:** | | | | |
| --- | --- | --- | --- | --- |
| **Risk of bias** | **Across studies** | **Interpretation** | **Considerations** | **GRADE assessment of study limitations** |
| low risk of bias | Most information is from studies at low risk of bias. | Plausible bias unlikely to seriously alter the results. | No apparent limitations. | No serious limitations, do not downgrade. |
| Unclear risk of bias | Most information is from studies at low or unclear risk of bias. | The plausible bias that raises some doubt about the results. | Potential limitations are unlikely to lower confidence in the estimate of effect. | No serious limitations, do not downgrade. |
|  |  |  | Potential limitations are likely to lower confidence in the estimate of effect. | Serious limitations, downgrade one level. |
| high risk of bias | The proportion of information from studies at high risk of bias is sufficient to affect the interpretation of results. | The plausible bias that seriously weakens confidence in the results. | Crucial limitation for one criterion, or some limitations for multiple criteria, sufficient to lower confidence in the estimate of effect. | Serious limitations, downgrade one level. |
|  |  |  | Crucial limitation for one or more criteria sufficient to substantially lower confidence in the estimate of effect. | Very serious limitations, downgrade two levels. |

Abberivations

ADL Activity of daily living

BI Barthel Index

CI Confidence Interval

FMA Fugl-Meyer-Assesment

mRS modified Rankin Scale

NMES neuromuscular electrostimulation

OIS optimal information size

RAGT robotic-assisted gait-training

**References**

1. Murad MH, Mustafa RA, Schünemann HJ, Sultan S, Santesso N. Rating the certainty in evidence in the absence of a single estimate of effect. Evid Based Med. Juni 2017;22(3):85–7.

2. Schünemann H, Brożek J, Guyatt G, Oxman A, editors. GRADE handbook for grading quality of evidence and strength of recommendations. GRADE handbook for grading quality of evidence and strength of recommendations. Updated October 2013. The GRADE Working Group, 2013. [Internet]. [zitiert 30. April 2022]. Verfügbar unter: guidelinedevelopment.org/handbook

3. Di Lauro A, Pellegrino L, Savastano G, Ferraro C, Fusco M, Balzarano F, u. a. A randomized trial on the efficacy of intensive rehabilitation in the acute phase of ischemic stroke. Journal of Neurology. 1. Oktober 2003;250(10):1206–8.

4. AVERT Trial Collaboration group. Efficacy and safety of very early mobilisation within 24 h of stroke onset (AVERT): a randomised controlled trial. The Lancet. Juli 2015;386(9988):46–55.

5. Langhorne P; Wu O; Rodgers H; Ashburn A; Bernhardt J; on behalf of the AVERT triallists’ collaboration. A very early rehabilitation trial after stroke (AVERT): a phase III, multicentre, randomised controlled trial [with consumer summary]. Health Technology Assessment (Winchester, England) 2017 Sep;21(54):1-120. 2017;

6. Lincoln NB, Parry RH, Vass CD. Randomized, Controlled Trial to Evaluate Increased Intensity of Physiotherapy Treatment of Arm Function After Stroke. Stroke. März 1999;30(3):573–9.

7. Rahayu UB, Wibowo S, Setyopranoto I, Hibatullah Romli M. Effectiveness of physiotherapy interventions in brain plasticity, balance and functional ability in stroke survivors: A randomized controlled trial. NeuroRehabilitation. 2020;47(4):463–70.

8. Guo GY, Kang YG. Effectiveness of neuromuscular electrical stimulation therapy in patients with urinary incontinence after stroke: A randomized sham controlled trial. Medicine (Baltimore). Dezember 2018;97(52):e13702.

9. Rosewilliam S, Malhotra S, Roffe C, Jones P, Pandyan AD. Can Surface Neuromuscular Electrical Stimulation of the Wrist and Hand Combined With Routine Therapy Facilitate Recovery of Arm Function in Patients With Stroke? Archives of Physical Medicine and Rehabilitation. Oktober 2012;93(10):1715-1721.e1.

10. Bagley P, Hudson M, Forster A, Smith J, Young J. A randomized trial evaluation of the Oswestry Standing Frame for patients after stroke. Clin Rehabil. Juni 2005;19(4):354–64.

11. Logan A; Freeman J; Kent B; Pooler J; Creanor S; Enki D; Vickery J; Barton A; Marsden J. Functional standing frame programme early after severe sub-acute stroke (SPIRES): a randomised controlled feasibility trial. Pilot and Feasibility Studies 2022 Mar 3;8(50):Epub. 2022;

12. Kwakkel G, Wagenaar RC, Twisk JW, Lankhorst GJ, Koetsier JC. Intensity of leg and arm training after primary middle-cerebral-artery stroke: a randomised trial. The Lancet. Juli 1999;354(9174):191–6.

13. Tang C, Ding Z, Li C, Chen C, Ding L, Zhang X, u. a. Effects of motor imagery combined with task-oriented training on the recovery of upper extremity function after stroke. Chinese Journal of Physical Medicine and Rehabilitation. 2017;36(11):832–7.

14. Stevenson TJ. Detecting change in patients with stroke using the Berg Balance Scale. Australian Journal of Physiotherapy. 2001;47(1):29–38.

15. Rahayu UB, Wibowo S, Setyopranoto I, Hibatullah Romli M. Effectiveness of physiotherapy interventions in brain plasticity, balance and functional ability in stroke survivors: A randomized controlled trial. NRE. 22. Dezember 2020;47(4):463–70.

16. Tang Q, Tan L, Li B, Huang X, Ouyang C, Zhan H, u. a. Early Sitting, Standing, and Walking in Conjunction With Contemporary Bobath Approach for Stroke Patients With Severe Motor Deficit. Topics in Stroke Rehabilitation. März 2014;21(2):120–7.

17. Ochi M, Wada F, Saeki S, Hachisuka K. Gait training in subacute non-ambulatory stroke patients using a full weight-bearing gait-assistance robot: A prospective, randomized, open, blinded-endpoint trial. Journal of the Neurological Sciences. Juni 2015;353(1–2):130–6.

18. Chang WH, Kim MS, Huh JP, Lee PKW, Kim YH. Effects of Robot-Assisted Gait Training on Cardiopulmonary Fitness in Subacute Stroke Patients: A Randomized Controlled Study. Neurorehabil Neural Repair. Mai 2012;26(4):318–24.

19. Louie DR, Mortenson WB, Durocher M, Schneeberg A, Teasell R, Yao J, u. a. Efficacy of an exoskeleton-based physical therapy program for non-ambulatory patients during subacute stroke rehabilitation: a randomized controlled trial. Journal of neuroengineering and rehabilitation. 2021;18(1):149.

20. Bai Y long, Hu Y shan, Wu Y, Zhu Y lian, Zhang B, Jiang C yu, u. a. Long-term three-stage rehabilitation intervention alleviates spasticity of the elbows, fingers, and plantar flexors and improves activities of daily living in ischemic stroke patients: a randomized, controlled trial. NeuroReport. 10. September 2014;25(13):998–1005.

21. Choi HS, Shin WS, Bang DH. Application of digital practice to improve head movement, visual perception and activities of daily living for subacute stroke patients with unilateral spatial neglect: Preliminary results of a single-blinded, randomized controlled trial. Medicine. 12. Februar 2021;100(6):e24637.

22. Sim T, Kwon J. Comparing the effectiveness of bimanual and unimanual mirror therapy in unilateral neglect after stroke: a pilot study. NeuroRehabilitation. 2022;50(1):133‐141.

23. Calabro RS, Accorinti M, Porcari B, Carioti L, Ciatto L, Billeri L, u. a. Does hand robotic rehabilitation improve motor function by rebalancing interhemispheric connectivity after chronic stroke? Encouraging data from a randomised-clinical-trial. CLINICAL NEUROPHYSIOLOGY. Mai 2019;130(5):767–80.

24. Chen JC, Lin CH, Wei YC, Hsiao J, Liang CC. Facilitation of motor and balance recovery by thermal intervention for the paretic lower limb of acute stroke: a single-blind randomized clinical trial. Clin Rehabil. September 2011;25(9):823–32.

25. Cui W, Huang L, Tian Y, Luo H, Chen S, Yang Y, u. a. Effect and mechanism of mirror therapy on lower limb rehabilitation after ischemic stroke: a fMRI study. NeuroRehabilitation. 2022;51(1):65‐77.

26. Brunelli S, Iosa M, Fusco F, Pirri C, Di Giunta C, Foti C, u. a. Early body weight-supported overground walking training in patients with stroke in subacute phase compared to conventional physiotherapy: a randomized controlled pilot study. International journal of rehabilitation research Internationale Zeitschrift fur Rehabilitationsforschung Revue internationale de recherches de readaptation. 2019;42(4):309‐315.

27. Rodrigues TA, Goroso DG, Westgate PM, Carrico C, Batistella LR, Sawaki L. Slow Versus Fast Robot-Assisted Locomotor Training After Severe Stroke: A Randomized Controlled Trial. American Journal of Physical Medicine & Rehabilitation. Oktober 2017;96(10):S165–70.

28. Kim NH, Park HY, Son JK, Moon Y, Lee JH, Cha YJ. Comparison of underwater gait training and overground gait training for improving the walking and balancing ability of patients with severe hemiplegic stroke: A randomized controlled pilot trial. Gait & Posture. Juli 2020;80:124–9.

29. Thimabut N, Yotnuengnit P, Charoenlimprasert J, Sillapachai T, Hirano S, Saitoh E, u. a. Effects of the Robot-Assisted Gait Training Device Plus Physiotherapy in Improving Ambulatory Functions in Patients With Subacute Stroke With Hemiplegia: an Assessor-Blinded, Randomized Controlled Trial. Archives of physical medicine and rehabilitation. 2022;103(5):843‐850.

30. Zheng X, Chen D, Yan T, Jin D, Zhuang Z, Tan Z, u. a. A Randomized Clinical Trial of a Functional Electrical Stimulation Mimic to Gait Promotes Motor Recovery and Brain Remodeling in Acute Stroke. Behavioural Neurology. 18. Dezember 2018;2018:1–10.

31. Shao C, Wang Y, Gou H, Xiao H, Chen T. Strength Training of the Nonhemiplegic Side Promotes Motor Function Recovery in Patients With Stroke: a Randomized Controlled Trial. Archives of physical medicine and rehabilitation. 2023;104(2):188‐194.

32. Lee SH, Prak JS, Choi JB, Yoo WG. Improving upper extremity motor function in stroke patients using a complex task with multi-joint-based mirror therapy: A randomized controlled trial. Neurology Asia. 2020;25(3):245–51.

33. Katz-Leurer M, Carmeli E, Shochina M. The effect of early aerobic training on independence six months post stroke. Clin Rehabil. November 2003;17(7):735–41.

34. Bagley P, Hudson M, Forster A, Smith J, Young J. A randomized trial evaluation of the Oswestry Standing Frame for patients after stroke. Clin Rehabil. Juni 2005;19(4):354–64.

35. Kim M, Lee S, Park E, Choi M, Kim J, Sohn M, u. a. Elastic Dynamic Sling on Subluxation of Hemiplegic Shoulder in Patients with Subacute Stroke: a Multicenter Randomized Controlled Trial. International journal of environmental research and public health [Internet]. 2022;19(16). Verfügbar unter: https://www.cochranelibrary.com/central/doi/10.1002/central/CN-02456101/full

36. An CM, Ko MH, Kim DH, Kim GW. Effect of postural training using a whole-body tilt apparatus in subacute stroke patients with lateropulsion: A single-blinded randomized controlled trial. Annals of physical and rehabilitation medicine. 2021;64(2):101393.
